# Synthesis, optical, electrochemical, and computational study of benzene/thiophene based D–π–A chromophores†

**DOI:** 10.1039/d4ra02668c

**Published:** 2024-11-05

**Authors:** Michaela Babejová, Iveta Třísková, Libuše Trnková, Hugo Semrád, Markéta Munzarová, Dominik Heger, Dana Nachtigallová, Milan Potáček

**Affiliations:** a Department of Chemistry, Faculty of Science, Masaryk University Kotlářská 2 CZ 611 37 Brno Czech Republic potacek@chemi.muni.cz; b Institute of Organic Chemistry and Biochemistry of the CAS Flemingovo nám. 2 160 00 Praha 6 Czech Republic

## Abstract

We report the design, synthesis, electrochemical, UV-vis, fluorescence, and computational study of nine π-linked donor–acceptor (D–π–A) chromophores. The series of novel compounds comprises a terphenyl, terthiophene, or 2,5-diphenyl thiophene linker, with one electron-donating group (methyl or *p-N*,*N*-diethyl) and one electron-withdrawing group (nitrone, formyl, or dicyanovinyl) at opposite ends of the molecule. The HOMO–LUMO gaps were determined *via* cyclic voltammetry and found to correspond well to DFT-calculated values. Furthermore, the influence of the π-linker character and substituent on the HOMO–LUMO gap was analysed and interpreted in terms of MO composition *via* DFT.

## Abstract 2

Synthesis of novel (D–π–A) chromophores with one electron-donating + withdrawing group at opposite ends of terphenyl, terthiophene, or 2,5-diphenylthiophene, analysed upon results of HOMO–LUMO gaps, determined *via* CV and in terms of MO *via* DFT.

## Graphical abstract

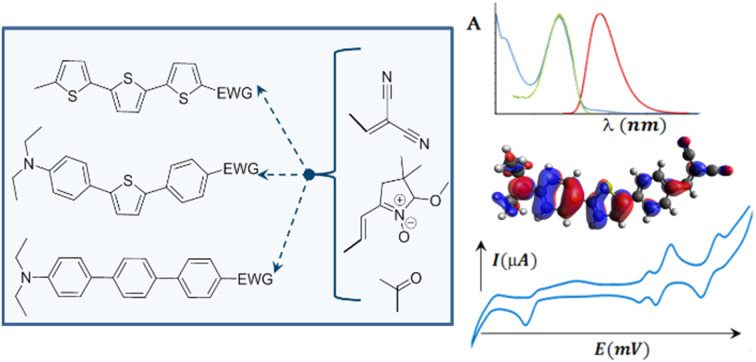

## 1. Introduction

Push-pull systems, characterized by a D–π–A arrangement with an electron donor on one end and an electron acceptor on the other, have garnered significant attention due to their potential for practical applications. These systems typically exhibit extended π-conjugation, allowing for enhanced nonlinear optical properties.^1–3^ The intramolecular charge transfer within these molecules, facilitated by the π-linkages connecting the donor and acceptor moieties, leads to intriguing photophysics in both ground and excited states.^1–3^ Such donor–acceptor molecules are valuable in various fields like solar energy conversion, molecular electronics, photovoltaics, and light-emitting diodes.^2^

Hence, there has been a rapid increase in the number of synthesized new molecular materials, potentially applicable in organic electronics and nonlinear optics in the last two to three decades. This progress has been accompanied by the characterization of these materials, mainly determining the energies of ionized electronic states. In addition to optical methods (UV photoelectron and inverse photoelectron spectroscopies – UPS, IPES)^4–7^ which may not always be accessible, cyclic voltammetry can be applied to determine reduction and oxidation potentials.^8,9^

Such organic π-systems have become extensively investigated, representing a new area of organic chemistry. They appear as active substances in organic electronics and optoelectronics,^10–12^ as conductors, and as compounds with photovoltaic properties.^13,14^ Due to the donor–acceptor interaction within a molecule, a new low-energy molecular orbital (MO) is formed, with a facile excitation of the electrons using visible light, making push–pull molecules coloured and referred to as charge-transfer chromophores.^15^

In the D–π–A, the structure of the π-bridge, responsible for the transfer of electrons between D and A, is crucial. Efficient donors and acceptors include dialkylamino or alkoxy substituents and cyano groups or dicyanoimidazole, respectively.^16–19^ The electron transfer primarily occurs through the chain of double or triple bonds. Various aromatic compounds have been tested, including 1,4-phenylene and some heterocyclic molecules. Systems with different thiophene-bound molecules are also commonly encountered. Our synthetic work aimed to create a new collection of conjugated polarized systems composed of aromatic molecules. We aimed to investigate how the type of aromatic molecule chain and their arrangement affect the delocalization of π–electrons and charge. Specifically, we synthesized and studied systems containing benzene and thiophene rings, organized in various sequences as bridges, with formyl, nitrone, or dicyanovinyl as electron acceptors. Additionally, we intended to evaluate the acceptor properties of nitrone compared to other known acceptors, given our previous experience with its successful yet challenging synthesis.

The effect of the new bridges between the selected donor and acceptor was investigated using cyclic voltammetry^8,9^ and UV-vis spectroscopy. Voltammetry is an inexpensive, fast, and practical method that uses the polarization of working electrodes to both positive and negative potential values, providing oxidation and reduction signals in a given push–pull system. The recorded redox signals reflect the HOMO and LUMO energies; the potential for the initial injection of holes (into the HOMO) and electrons (into the LUMO) is determined by the onset potential, *E*_onset,ox_ and *E*_onset,red_ for oxidation and reduction, respectively.^20,21^

The values of the HOMO–LUMO gap energies obtained experimentally were compared with the theoretical values calculated by the DFT method. The calculations aimed to qualitatively investigate the electronic structure of push–pull systems in terms of electron density distribution across prepared molecules and assess the electron–acceptor properties of the three-ring bridge structure.

## 2. Synthesis

**Scheme 1:**
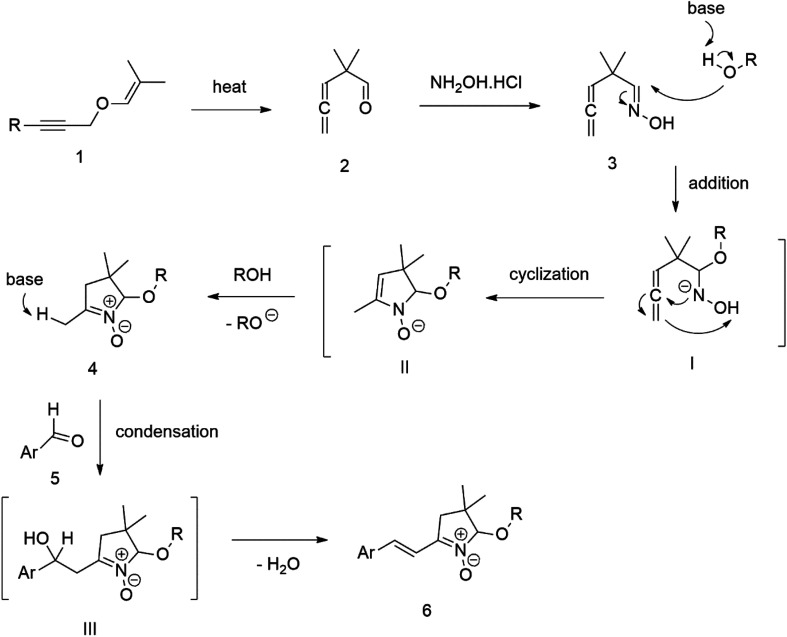


Our success and experience with the new synthesis of nitrones^22–24^ led us to apply the nitrone moiety as an electron-withdrawing group on one side of the conjugated system. Additionally, dicyanovinyl and aldehyde groups were used as acceptors in the synthesized molecules for comparison. Our first strategy to prepare the sets of conjugated molecules was based on our previous experience^22–24^ with nitrones preparation, which was expanded by their further condensation with aromatic aldehydes. The aldehydes containing a halogen atom in a proper position were then used in efficient Suzuki–Miyaura coupling^25^ because it is known as a powerful and reliable method for extending aromatic systems to prepare the desired products. We intended to apply this approach in synthesizing new push–pull delocalized systems consisting of benzene and thiophene rings. The starting synthesis was based upon thermally initiated Claisen rearrangement of propargyl vinyl ether 1 leading to an allene 2 (Scheme 1).

When reacting with hydroxylamine hydrochloride in dichloromethane, the formed allene yields corresponding oxime 3. Upon heating in the presence of a catalytic amount of KOH, the cyclization to nitrone 4 occurs. Scheme 1 shows the simplified depiction of the reaction mechanism.

During our experiments, we observed that the reaction (Scheme 1) was accompanied by exchanging the OH group for OR from the alcohol solvent. The reaction of nitrone molecule 4 (Scheme 1) with *p*-bromobenzaldehyde in methanol in the presence of KOH was successful, yielding a *p*-brominated aromatic 6 in a substantial amount. This one-pot procedure achieved the highest product yield of 85%, making it suitable for synthesizing brominated aromatics 6. Repeating the reaction with halogenated thiophene, 5-bromothiophene-2-carbaldehyde, under the same conditions again yielded excellent results.

Compound 6 was consequently chosen as a starting material for the Suzuki–Miyaura coupling procedure of aromatic boronic acids, with the aim of creating new molecules featuring an extended π-conjugated system. Unfortunately, our experiments aimed at prolonging the conjugated aromatic part of the molecule through Suzuki–Miyaura coupling *via p*-bromo-substituted Ar in molecule 6, did not yield the anticipated results. Although this coupling method is considered powerful and reliable for similar reactions, in our case, it was not found sufficiently effective for the expected further extension of the aromatic system Ar in molecule 6 (Scheme 1).

The extension was tested with both types of brominated aromatic systems, with 4-bromophenyl and 2-bromothienyl. Even after varying the catalysts used, such as (PPh_3_)_4_Pd, PdCl_2_ (dppf), and adding K_3_PO_4_, as well as experimenting with different solvents (DMF, H_2_O) and temperatures, we did not achieve the anticipated positive results.

**Scheme 2:**
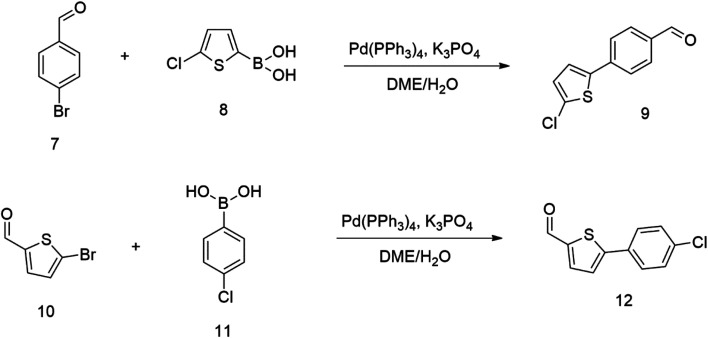


Therefore we started synthesizing the required structure from 4-chloro substituted phenyl boronic acid 11 and corresponding 5-bromo substituted aromatic aldehyde 10. This approach's initial attempts yielded the expected products with yields exceeding 70% (Scheme 2). Therefore, this successful procedure was considered as a suitable model synthesis for extending further aromatic chains.

**Scheme 3:**
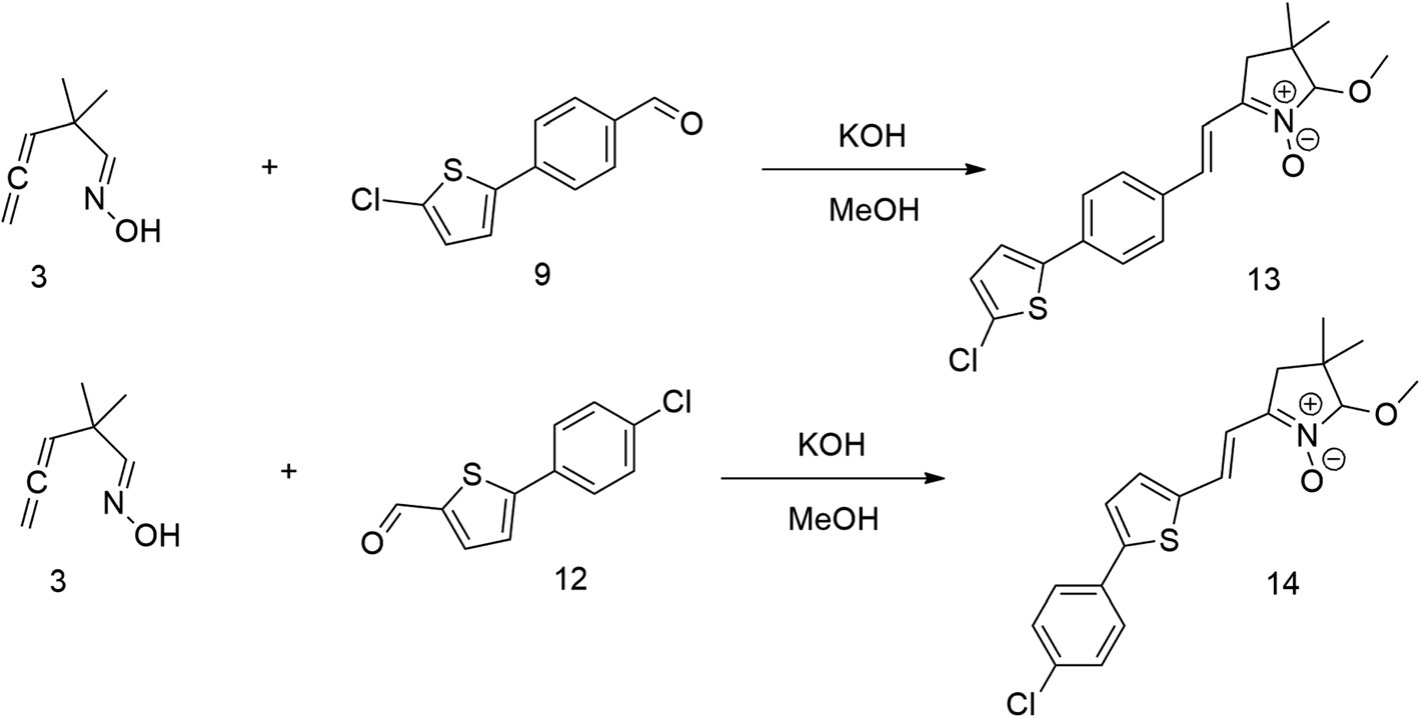


The relatively low reactive chlorine atoms in 9 and 12, which were needed for the final extension, were inactive in the reaction of 2,2-dimethylpenta-3,4-dienaloxime 3 and the present aldehyde group, simultaneously forming nitrones conjugated with aromatic systems and creating compounds 13 and 14 (Scheme 3). This reaction appeared to be suitable for further prolongation of the aromatic system.

However, the previously mentioned low reactivity of the chlorine atom in 13 or 14 proved problematic in the subsequent Suzuki coupling reaction, as it hindered the reaction with the extended aromatic system. This issue was initially addressed by substituting chlorine for bromine atom. Nonetheless, a change in strategy, specifically, returning to introducing acceptors in the final synthetic step, was ultimately found to be more effective and desirable.

**Scheme 4:**
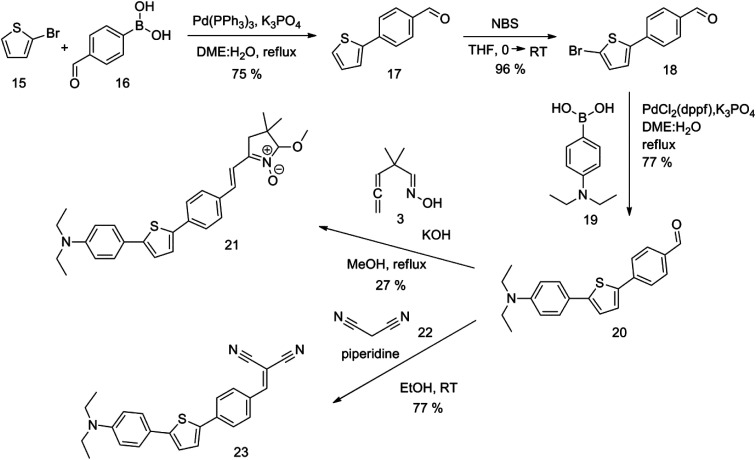


The new strategy was thus initiated, focusing on the prior synthesis of a chain composed of two benzene rings with a thiophene ring in the middle (compound 20). Although the synthesis of compound 20 is documented in the literature,^26^ and the syntheses of intermediates 17 and 18 are superficially described within the context of drug synthesis, we began by reacting aromatic boronic acid 16, containing a formyl group, with 2-bromothiophene 15 (Scheme 4). This reaction produced a joined aromatic molecule with a reactive aldehyde group (compound 17). In the subsequent step to obtain compound 18, we applied NBS at low temperatures, resulting in the required brominated aromatic ring with an aldehyde group. This product served as the starting material for further Suzuki coupling, leading to the preparation of systems with three conjugated aromatic molecules. Using this approach, compound 18 was reacted with 4-*N*,*N*-diethylphenylboronic acid 19 to form a chain of three aromatic rings (compound 20). Further new molecules were synthesized either by reacting compound 20 with oxime 3, which introduced a nitrone skeleton to form compound 21, or by reacting with malononitrile to prepare a new compound 23 with a dicyanovinyl substitution (Scheme 4).

**Scheme 5:**
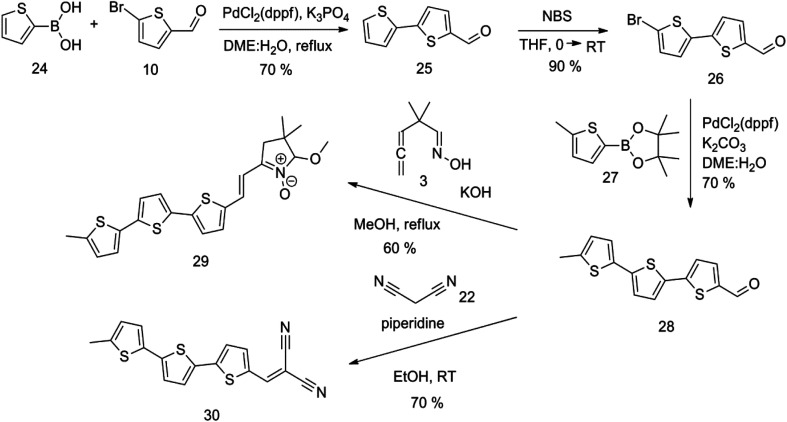


**Scheme 6:**
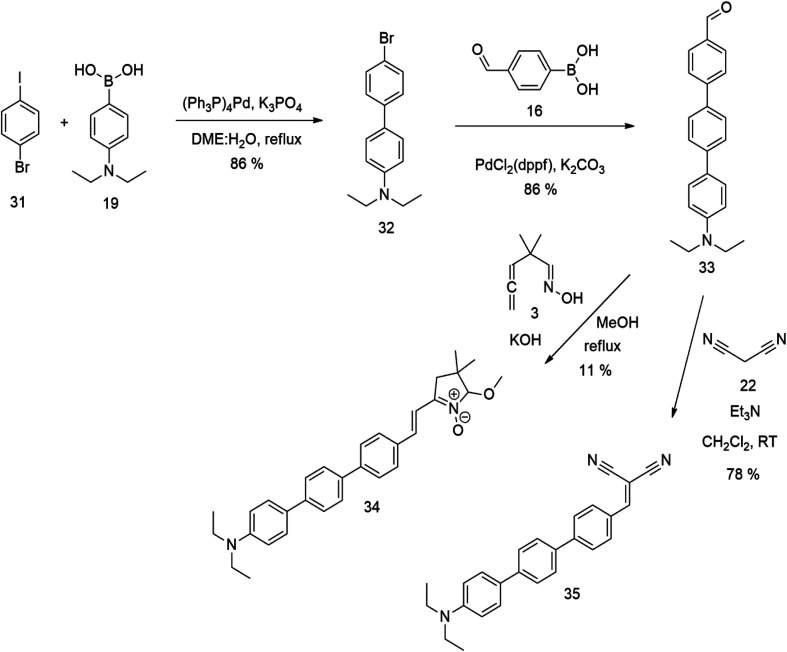


An overview of the mentioned strategy ultimately proved successful for synthesizing the other intended molecules, which can be seen in Schemes 5 and 6.

Synthesis of compound 28 with terthiophene bridge is covered by 3 patents. We decided to prepare the compound 28 by our synthetic procedure. We initiated the reaction with 5-bromothiophene-2-carbaldehyde 10 and 2-thiophene boronic acid 24. Although the synthesis of intermediate 26 from 1-bromo-5-methylthiophene under PdCl_2_ catalysis under microwave irradiation and other similar procedures and conditions are documented in the literature,^27^ we preferred to use our already proven procedure. Subsequent bromination compound of 25 using NBS at low temperature yielded compound 26 efficiently. This product successfully underwent a reaction with pinacol ester of boronic acid 27, resulting in the formation of the desired new compound 28 The aldehyde group of 28 facilitated the synthesis of additional new molecules: the reaction with oxime 3 yielded the corresponding nitrone molecule 29, and the reaction with malononitrile produced compound 30.

The literature on the synthesis of terphenyl bridges is rather extensive, encompassing numerous processes, most of which are covered by patents, using the classic approach from halogenated benzene or dihalogenated benzene and phenyl boronic acid in the presence of a suitable Pd catalyst (Suzuki coupling). In the older records, high temperatures were often used in the place of the catalyst. For specific patterns, you can refer to the literature.^28–33^ Compound 33 has not been published yet, but the synthesis of its precursor formyl-terphenyl compound is available in the literature.^34–37^ Similarly, the modification of the 4-amino terphenyl molecule to be substituted by *N*,*N*-diethyl-molecule is described in work.^38^ In our case, we decided to use our established procedures for preparing the unknown compound 33 and the previously presented methods for synthesizing the final structures 34 and 35.

Details of all the syntheses, and the structure analysis of all prepared compounds and their physical properties, are fully described in ESI.†

## 3. Electrochemical study

**Fig. 1:**
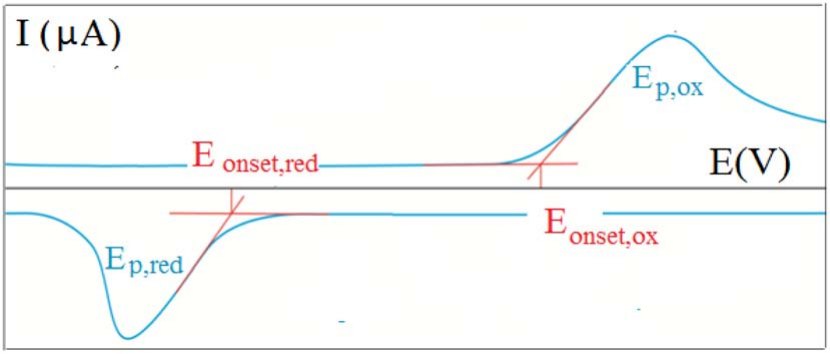
Graphical representation of a cyclic voltammetric recording with an example of the onset potentials.

To obtain a deeper insight into the ground state properties and the mutual donor–acceptor electronic influence, we investigated the redox properties of our new push–pull systems by cyclic voltammetry. All voltammetric measurements were performed by using the electrochemical analyzer μAUTOLAB TYPE III (Metrohm, Switzerland) controlled by GPES 4.9 software. The newly synthesized derivatives (1 mM; in dichloromethane) with the addition of 0.1 M ferrocene solution as internal standard (in dichloromethane) were dosed into the voltammetric cell occupied with three electrodes – platinum electrode (effective area of 7,1 mm^2^) as the working electrode, Ag/AgCl/3M KCl, and Pt wire as the reference and auxiliary electrodes, respectively. The 0.1M [NBu_4_][PF_6_] was used as the supporting electrolyte. The experimental conditions were as follows: potential range: from 0 V to +2 V (oxidation process) and/or from 0 V to −2 V (reduction process), scan rate: 75 mV s^−1^, potential step: 2 mV, inert argon atmosphere, room temperature. CV records allow for determining the onset potentials, which are obtained as the intersections of two tangents for the oxidation (*E*_onset, ox_) and reduction (*E*_onset, red_) peaks as shown in Fig. 1.^20^

**Fig. 2:**
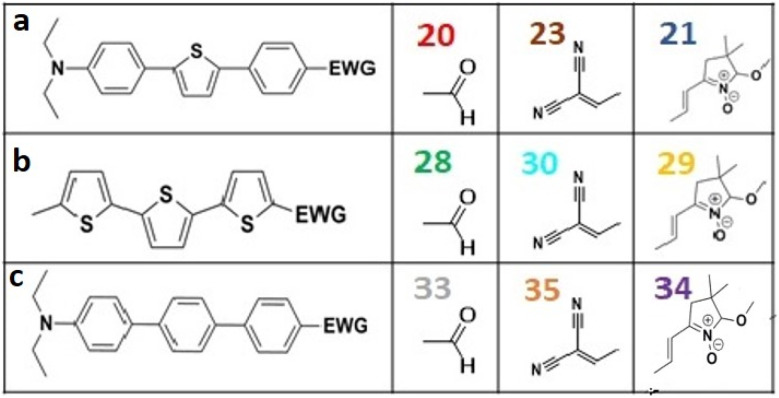
The structures of prepared aromatic conjugated D–π–A derivatives.

**Fig. 3:**
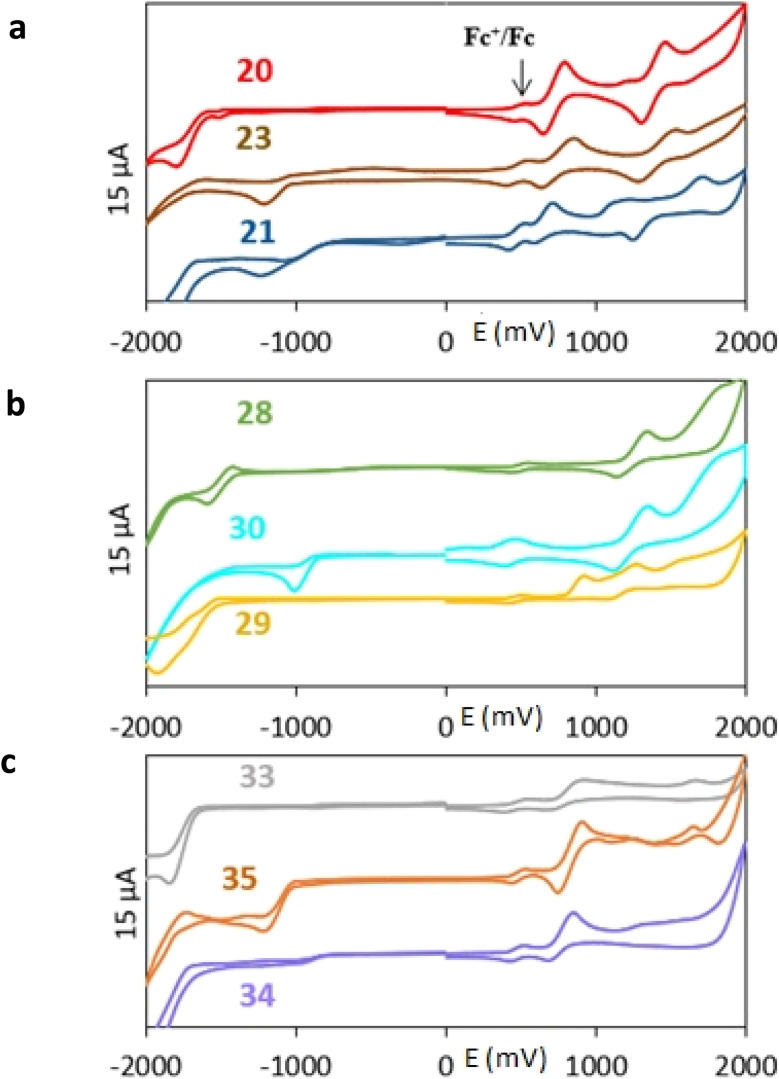
Cyclic voltammograms of all D–π–A derivatives (1 mM) – (a) the compounds with the 2,5-diphenylthiophene bridge; (b) the compounds with the terthiophene bridge; (c) the compounds with the terphenyl bridge, all on the platinum electrode in dichloromethane with the basic electrolyte of 0.1 M ([NBu_4_][PF_6_]). The potential scale corresponds to the Ag/AgCl reference electrode.

All nine prepared aromatic conjugated D–π–A derivatives (Fig. 2) exhibit oxidation voltammetric peaks when the working Pt electrode was polarized to positive potentials and reduction voltammetric peaks when the working electrode was polarized to negative potentials, each time starting from zero potential (Fig. 3). The onset potentials *E*_onset, ox_ and *E*_onset, red_ were determined from the first oxidation signal and the first reduction signal, respectively. For better clarity, the D–π–A derivatives are marked with colour numbers, and the reversible redox signal of the internal standard ferrocenium/ferrocene (Fc^+^/Fc) is indicated by an arrow (Fig. 3).

A detailed description of the voltammetric experiment and its output for individual conjugates is presented in the ESI,† where an arrow for each derivative indicates the reversible wave of the internal standard.

To calculate the HOMO and LUMO energies, it is necessary to establish the reference electrochemical system related to the vacuum. For this purpose, the redox couple ferrocenium/ferrocene (Fc^+^/Fc) is used; it is a stable, relatively well-soluble complex in non-aqueous solvents, moreover, without a significant solvation load. Considering the formal potential Fc^+^/Fc in the selected solvent (CH_2_Cl_2_) with the basic electrolyte ([NBu_4_][PF_6_]) and converting to the potential of the reference electrode (Ag/AgCl/3MKCl) from the electrode SCE, the reference energy on the Fermi energy scale corresponds to −4.89 eV.^9,21,39–41^

The energies of the HOMO and LUMO orbitals are calculated according to the following equations eqn (1) and (2),^21^

1 *E*_HOMO_ = −(*E*_onset, ox_ − *E*°_Fc^+^/Fc_ + 4.89) eV

2 *E*_LUMO_ = −(*E*_onset, red_ − *E*°_Fc^+^/Fc_ + 4.89) eV

where *E*°_Fc^+^/Fc_ is the formal redox potential of the Fc^+^/Fc, calculated from its anodic (*E*_pa_) and cathodic (*E*_pc_) potentials, *i.e.*, *E*°_Fc^+^/Fc_ = (*E*_pa_ + *E*_pc_)/2. The oxidation (*E*_onset, ox_) and reduction (*E*_onset, red_) onset potentials, formal redox potential of the ferrocenium/ferrocene system (*E*°_Fc^+^/Fc_), HOMO (*E*_HOMO_) and LUMO (*E*_LUMO_) and band gap (*E*_g_) energies for the series of compounds are presented in Tables 1–3.

**Table 1:** Oxidation (*E*_onset, ox_) and reduction (*E*_onset, red_) onset potentials, formal redox potential of the ferrocenium/ferrocene system (*E*°_Fc^+^/Fc_), HOMO (*E*_HOMO_) and LUMO (*E*_LUMO_) and band gap (*E*_g_) energies for the series of compounds with the 2,5-diphenylthiophene bridge^a^

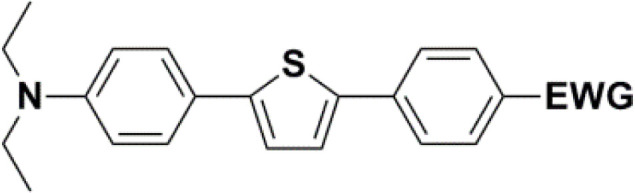	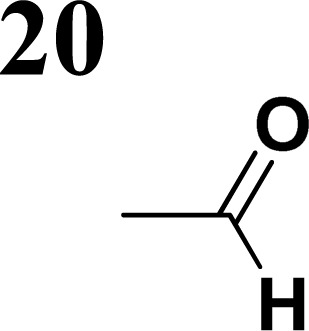	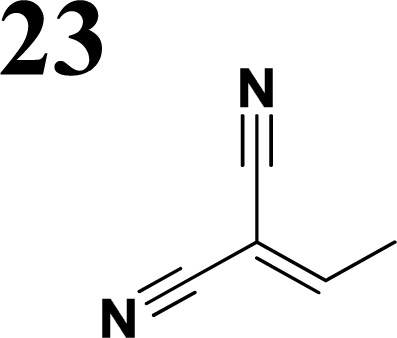	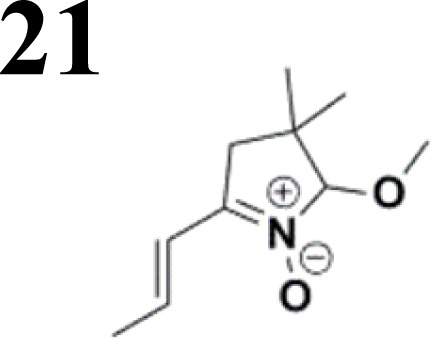
*E*_onset, ox_/V	0.65	0.67	0.60
*E*_onset, red_/V	−1.43	−1.05	−0.81
*E*°_Fc^+^/Fc_/V	0.49	0.47	0.47
*E*_HOMO_/eV	−5.05	−5.09	−5.02
*E*_LUMO_/eV	−2.96	−3.37	−3.61
***E***_**g**_ **/eV**	**2.09**	**1.72**	**1.41**

a Electron-withdrawing group (EWG).

**Table 2:** Oxidation (*E*_onset, ox_) and reduction (*E*_onset, red_) onset potentials, formal redox potential of the ferrocenium/ferrocene system (*E*°_Fc^+^/Fc_), HOMO (*E*_HOMO_) and LUMO (*E*_LUMO_) and band gap (*E*_g_) energies for the series of compounds with the bridge of terthiophenes^a^

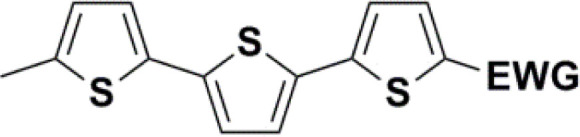	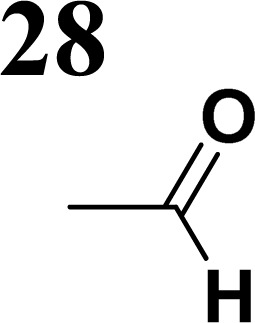	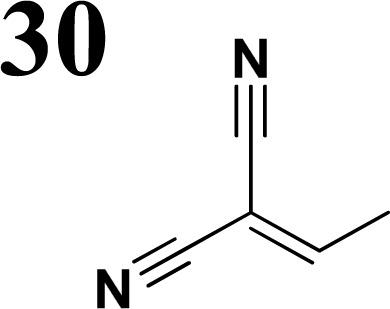	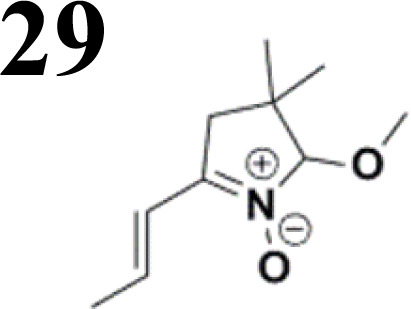
*E*_onset, ox_/V	1.19	1.17	0.81
*E*_onset, red_/V	−1.41	−0.88	−1.56
*E*°_Fc^+^/Fc_/V	0.50	0.43	0.47
*E*_HOMO_/eV	−5.57	−5.63	−5.23
*E*_LUMO_/eV	−2.98	−3.58	−2.86
***E***_**g**_ **/eV**	**2.60**	**2.04**	**2.37**

a Electron-withdrawing group (EWG).

**Table 3:** Oxidation (*E*_onset, ox_) and reduction (*E*_onset, red_) onset potentials, formal redox potential of the ferrocenium/ferrocene system (*E*°_Fc^+^/Fc_), HOMO (*E*_HOMO_) and LUMO (*E*_LUMO_) and band gap (*E*_g_) energies for the series of compounds with terphenyl bridge

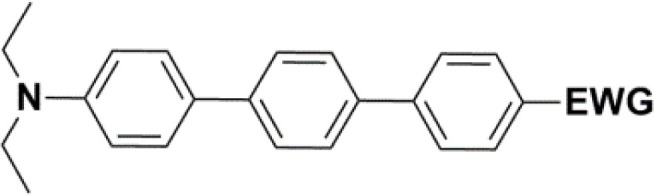	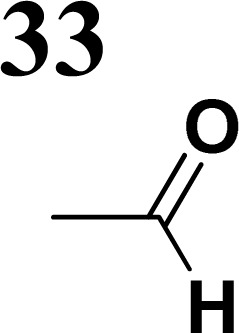	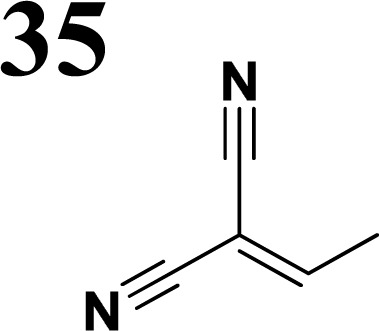	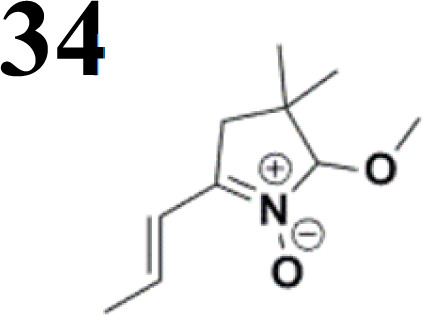
*E*_onset, ox_/V	0.73	0.75	0.70
*E*_onset, red_/V	−1.68	−1.04	−0.82
*E*°_Fc^+^/Fc_/V	0.47	0.49	0.48
*E*_HOMO_/eV	−5.15	−5.15	−5.11
*E*_LUMO_/eV	−2.74	−3.36	−3.59
***E***_**g**_ **/eV**	**2.41**	**1.79**	**1.52**

## 4. Spectral study

The UV-vis and fluorescence of all the synthesized molecules were measured in 0.01 mM dichloromethane solutions. Energy differences *E*_opt_ corresponding to electron transitions were calculated according to equation,^20^*E*_opt_ = *hc*/*λ* = 1242/*λ*_onset_where *h* is Planck's constant, *c* the speed of light, and *λ*_onset_ is an onset wavelength in nm (ESI VI†). The resulting spectra are given in ESI† and summarized for individual substituents in Tables 4–6.

**Table 4:** The maximum of lowest lying absorption (*λ*_max_) and emission (*λ*_em_,_max_), supplemented by the maximum of the excitation (*λ*_ex,max_), and the onset of S1 and S3 transitions and their vertical energies (*E*_opt_); n.d. stands for not detected

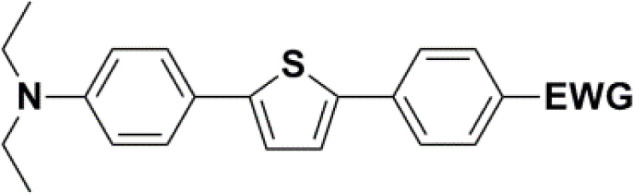	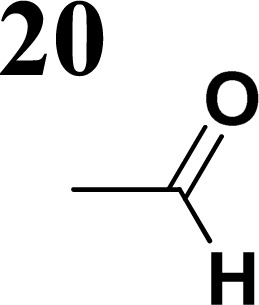	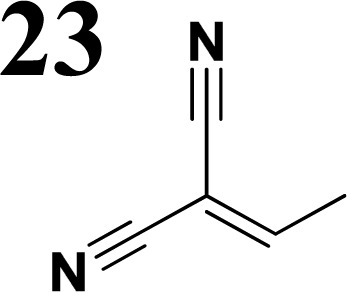	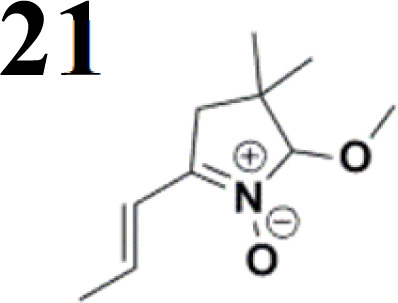
*λ*_max_/nm	423	506	425
*λ*_em, max_/nm	597	Two emission maxima	619
*λ*_ex, max_/nm	421	506	425
*λ*_onset_(S1)/nm	487	602	495
***E***_**opt**_ **/eV**	**2.55**	**2.06**	**2.51**
*λ*_onset_(S3)/nm	n.d.	390	457
***E***_**opt**_ **/eV**	n.d.	**3.18**	**2.71**

**Table 5:** The maximum of lowest lying absorption (*λ*_max_) and emission (*λ*_em, max_), supplemented by the maximum of the excitation (*λ*_ex, max_), and the onset of S1 transition and their vertical energies (*E*_opt_)

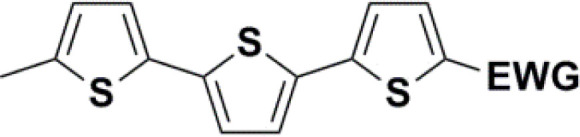	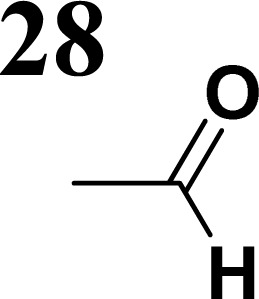	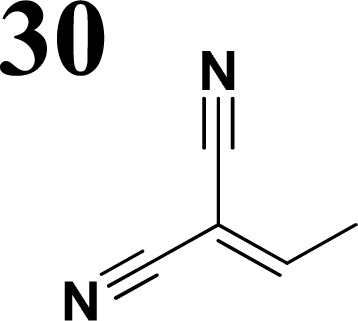	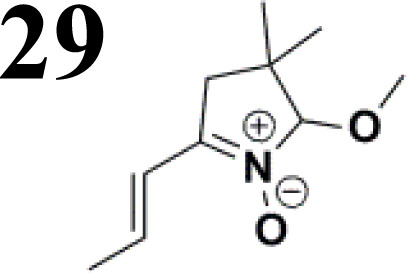
*λ*_max_/nm	408	488	402
*λ*_em, max_/nm	513	620	485
*λ*_ex, max_/nm	409	492	396
*λ*_onset_(S1)/nm	469	557	505
***E***_**opt**_ **/eV**	**2.65**	**2.23**	**2.46**

**Table 6:** The maximum of lowest lying absorption (*λ*_max_) and emission (*λ*_em_, _max_), supplemented by the maximum of the excitation (*λ*_ex, max_), and the onset of S1 and S3 transitions and their vertical energies (*E*_opt_)

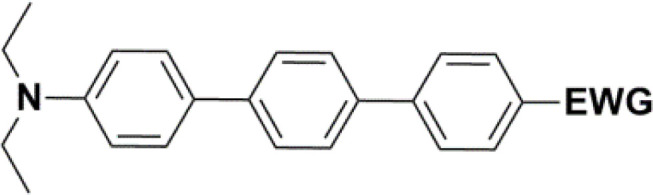	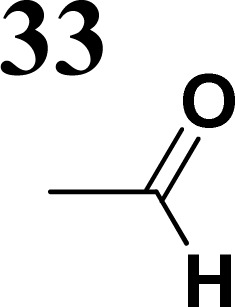	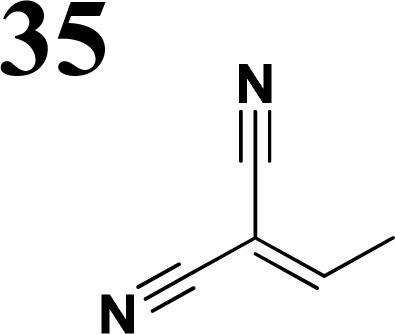	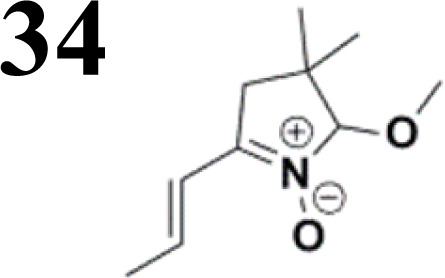
*λ*_max_/nm	371	326	378
*λ*_em, max_/nm	598	350	594
*λ*_ex, max_/nm	371	560	391
*λ*_onset_(S1)/nm	445	540	440
***E***_**opt**_ **/eV**	**2.79**	**2.30**	**2.83**
*λ*_onset_(S3)/nm	310	395	340
***E***_**opt**_ **/eV**	**4.00**	**3.14**	**3.65**

## 5. Computational studies

In order to qualitatively understand the outcomes of the electrochemical study, the electronic structure of push–pull systems was explored by means of quantum chemistry. All structures were optimized using the B3LYP functional^42–44^ along with the 6-31G* basis set^45,46^ and Grimme's GD3BJ dispersion correction.^47,48^ Gaussian 16, Rev. C.01 implementation^49^ has been employed with the grid variable set on the “ultrafine” value. For HOMO and LUMO energy estimation, geometry optimization was followed by single-point energy calculations at the B3LYP/6-311G*/GD3BJ level. The SMD solvation model^50^ has been involved in both calculation procedures, with dichloromethane chosen as the solvent. Molecular orbitals have been visualized by the Avogadro program, ver. 1.1.1. with the default value of MO isosurface.^51,52^

For selected systems, in particular molecules 20, 23, 28, and 33, the vertical excitation and emission spectra were calculated. These calculations were obtained with Algebraic Diagrammatic Construction to 2. Order (ADC(2)) method,^53,54^ and def2-TZVP^55^ for single-point calculations and def2-SVP for optimization. The vertical excitation energies were calculated as a difference of total energies of S_0_ and S_n_ states at the ground-state optimized geometries while the emission energies were obtained as an energy difference between S_n_ states at the equilibrium geometries of the S_n_ states. The Turbomole package program was used for these calculations.^56^

## 6. Interpretation of the HOMO–LUMO gaps *via* frontier Mo analysis

**Fig. 4:**
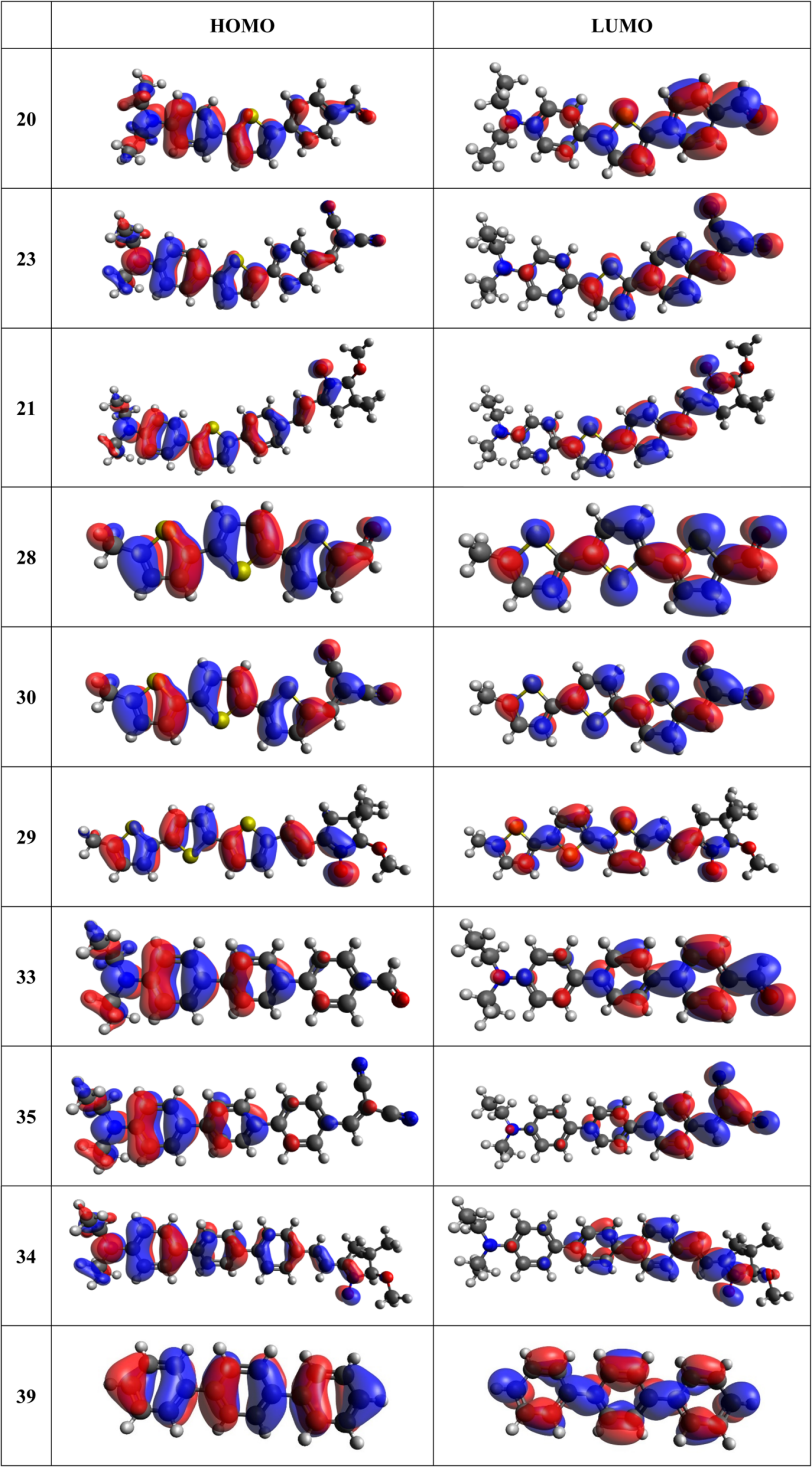
Frontier orbitals of D–π–A derivatives calculated at the B3LYP/6-311G* level, visualized by Avogadro 1.1.1 employing the default isosurface value.

Frontier MOs of push–pull molecules are shown in Fig. 4. Ordering of the systems is adjusted to stress the similarity of HOMO/LUMO shapes in molecular pairs: 20 and 23, 28 and 30, 33 and 35. Frontier MO isosurfaces demonstrate the extent of π-delocalization in the push–pull systems. The shortest chain is found in 28, where – disregarding hyperconjugation with the CH_3_ donor group – 13 bonds are concerned, starting from the methyl-substituted carbon and proceeding through the C–C linkages toward the aldehydic oxygen. For the aldehyde-substituted 2,5-diphenyl thiophene and terphenyl, 20 and 33, 14 bonds are conjugated due to the *N*,*N*-diethyl donor whose nitrogen atom is symmetry-allowed to participate in π-electron delocalization. Within the dicyanovinyl group, π-delocalization is two bonds longer than for the aldehyde electron acceptor, resulting in 16, 15, and 16 conjugated bonds for systems 23, 30, and 35, respectively. Finally, symmetry-allowed electron delocalization extends up to the negatively charged oxygen for the nitrone substituent, resulting in 17, 16, and 17 conjugated bonds for systems 21, 29, and 34, respectively.

Interestingly, not for all systems in Fig. 4 do HOMO and LUMO extend over the full number of conjugated bonds. While for 28–30, frontier MOs do indeed cover the complete π-skeleton, for 20–23, and especially for 33–35; HOMOs are more localized on the electron-donating end of the molecule and LUMOs on the electron-accepting end. This can be well understood considering the deviations of the push–pull systems from planarity. Table 7 summarizes dihedral angles between the planes of neighboring aromatic rings proceeding from the EDG towards EWG. While for 28–30, these are 0–11°, in 20–23, the dihedrals are already 8–18° and in 33–35, as much as 29–33°. In addition, Table 7 shows the calculated HOMO and LUMO of the non-substituted terphenyl molecule (39). There, the conjugation over the molecule contrasts with the partial localization of the electron densities in cases of 33–35.^57^

**Table 7:** Dihedral angles between aromatic ring planes

Structure	Dihedral angles
20	−18.2°/14.4°
23	13.6°/−8.0°
21	8.7°/12.7°
28	−0.5°/0.1°
30	−8.0°/0.0°
29	−10.5°/7.9°
33	29.9°/−32.5°
35	28.6°/−29.2°
34	30.7°/−32.6°
39	34.7°/−34.8°

Let us now compare HOMO and LUMO energies as obtained from electrochemical measurements (Tables 1–3), from UV-VIS spectroscopy (Tables 4–6), and from *ab initio* calculations (Tables 8–10). To decouple the influence of EDG from the other structural and electronic effects, the set of molecules explored was augmented by three theoretical counterparts (36–38) of systems 28–30 bearing the diethylamino (instead of CH_3_) electron donor group. HOMO and LUMO energies of the latter are summarized in Table 11. Focusing first on the experimentally studied molecules 20–35, we note that trends in HOMO/LUMO gaps obtained from calculations agree much more closely with the UV-vis values than with the electrochemically determined energy differences. Indeed, UV-vis energy gaps increase in the order 23 < 30 < 35 < 29 < 21 < 20 < 28 < 33 < 34. Theoretical values copy this trend except for switching 30 with 35 and 28 with 34. The agreement of theoretical and UV-vis energy gaps with the electrochemical values is much worse, possibly due to the influence of electrode polarization on the detected redox potentials.

**Table 8:** Frontier MO energies and their differences *E*_g_ for series of molecules with 2,5-diphenylthiophene bridge

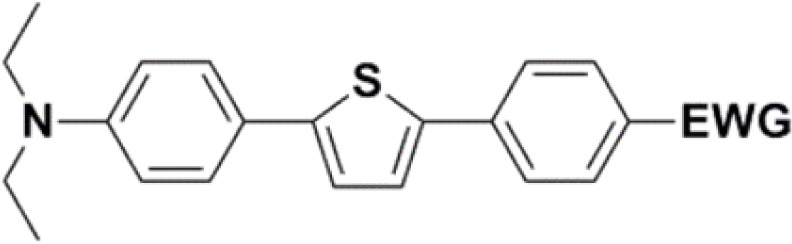	*E*_HOMO, calc_ [eV]	*E*_LUMO, calc_ [eV]	*E*_g, calc_ [eV]	*E*_HOMO, elch_ [eV]	*E*_LUMO, elch_ [eV]	*E*_g, elch_ [eV]	*E*_g,opt_ [eV]
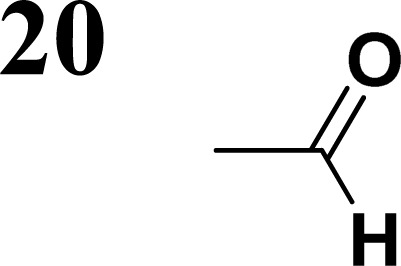	−4.94	−2.15	**2.79**	−5.05	−2.96	**2.09**	**2.55**
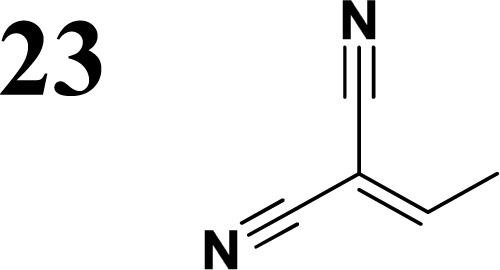	−4.99	−2.85	**2.14**	−5.09	−3.37	**1.72**	**2.06**
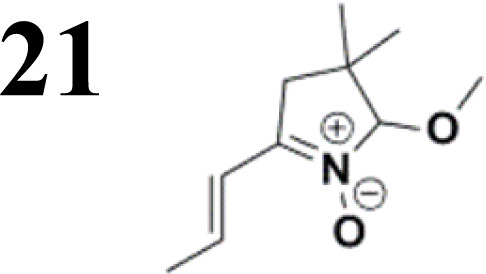	−4.76	−2.07	**2.69**	−5.02	−3.61	**1.41**	**2.51**

**Table 9:** Frontier MO energies and their differences *E*_g_ for series of molecules with terthiophene bridge

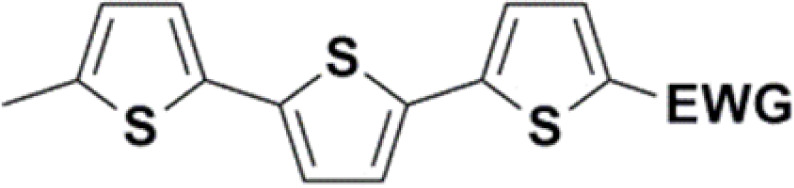	*E*_HOMO, calc_ [eV]	*E*_LUMO, calc_ [eV]	*E*_g, calc_ [eV]	*E*_HOMO, elch_ [eV]	*E*_LUMO, elch_ [eV]	*E*_g, elch_ [eV]	*E*_g,opt_ [eV]
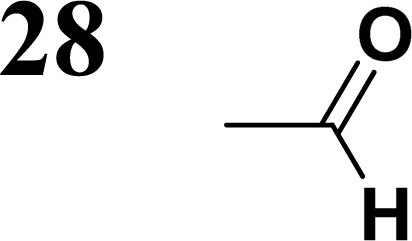	−5.46	−2.51	**2.95**	−5.57	−2.98	**2.59**	**2.65**
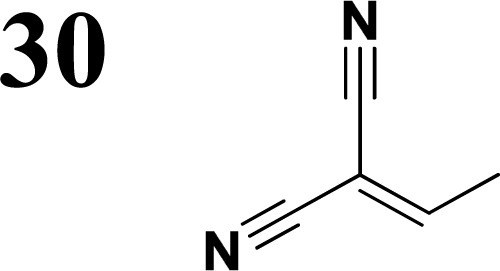	−5.54	−3.06	**2.48**	−5.63	−3.58	**2.05**	**2.23**
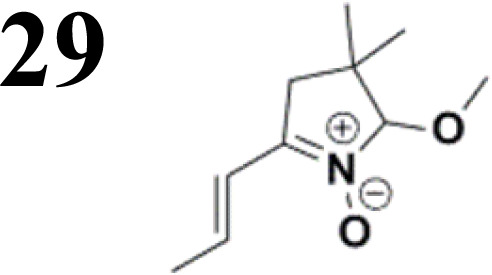	−4.95	−2.33	**2.62**	−5.23	−2.86	**2.37**	**2.46**

**Table 10:** Frontier MO energies and their differences *E*_g_ for series of compounds with terphenyl bridge

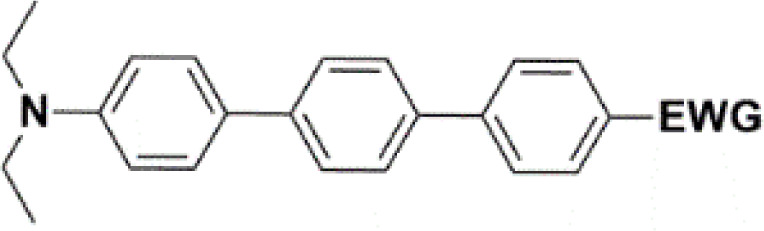	*E*_HOMO, calc_ [eV]	*E*_LUMO, calc_ [eV]	*E*_g, calc_ [eV]	*E*_HOMO, elch_ [eV]	*E*_LUMO, elch_ [eV]	*E*_g, elch_ [eV]	*E*_g,opt_ [eV]
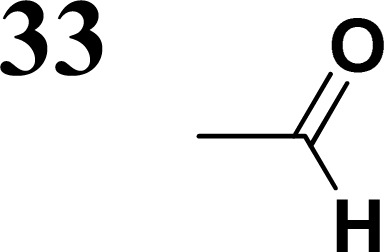	−5.00	−2.01	**2.99**	−5.15	−2.74	**2.41**	**2.79**
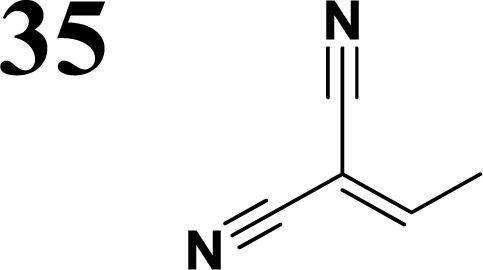	−5.04	−2.84	**2.20**	−5.15	−3.36	**1.79**	**2.30**
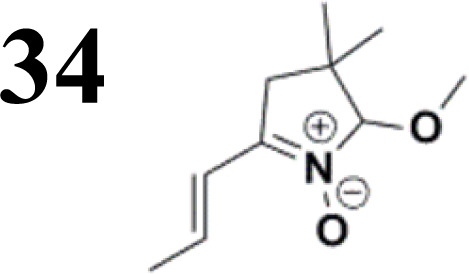	−4.92	−2.01	**2.91**	−5.11	−3.59	**1.52**	**2.83**

**Table 11:** Frontier MO energies and their differences, *E*_g_, for series of theoretically studied analogues of systems 28, 30, and 29 with EDG = diethylamine

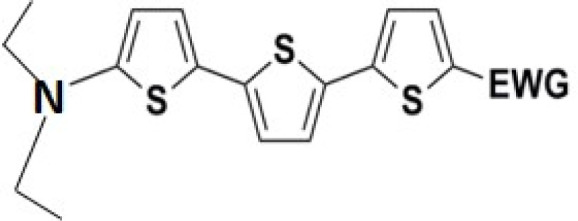	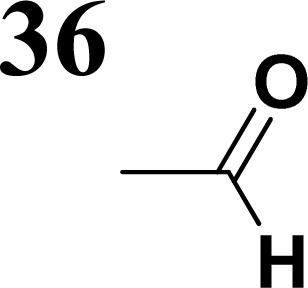	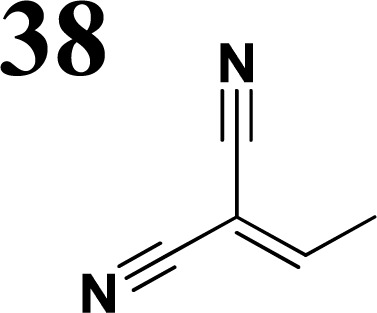	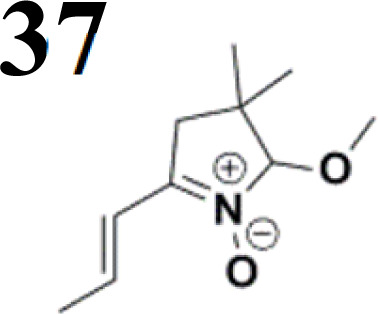
*E*_HOMO, calc_ [eV]	−4.75	−4.85	−4.53
*E*_LUMO, calc_ [eV]	−2.35	−2.90	−2.21
*E*_g, calc_ [eV]	**2.40**	**1.95**	**2.32**

The theoretical and UV-vis ordering of energy gaps cannot be understood solely based on conjugated chain length. Using the simple particle-in-a-box point of view, both HOMO and LUMO energies should grow with extending chain length, but their energy gap should decrease as suggested by Hans Kuhn as early as in 1949.^58^ Apparently, this simple trend is not followed: the smallest energy gap is found for systems with 16 (not 17) conjugated bonds, while the largest energy gap is for 14 (not 13) conjugated bonds. Obviously, the effects of distinct EDG and EWG (absent in Kuhn's work) compete with the chain length influence.

Several regular patterns emerge when ordering systems in Tables 8–11, including molecules 36–38, according to quantum-chemically determined MO energies: (a) for the same type of EWG, HOMO energy grows in the order methyl-substituted terthiophene, terphenyl, 2,5-diphenylthiophene, diethylamine-substituted terthiophene. (b) For the same type of donor-substituted π-chain, HOMO energy grows in the order dicyanovinyl, aldehyde, and nitrone. (c) Lowest LUMO energies are found for dicyanovinyl derivatives and highest LUMO energies for nitrone derivatives, disregarding the type of donor-substituted π-chain. From (b) and (c), it can be concluded that the dicyanovinyl group pulls both HOMO and LUMO energies down while nitrone group pushes both HOMO and LUMO energies up. This is understandable from the point of view of electronegativity perturbation by the strongly electron accepting dicyanovinyl group. Indeed, electronegativity perturbation pulls both occupied and unoccupied levels down in energy. On the other end, the nitrone group possesses not only the electron-accepting N–O unit but also the electron-donating vinyl unit. Thus, compared to the aldehyde EWG, nitrone is a weaker electron acceptor, which results in shifting both HOMO and LUMO levels up.

Considering the influence of the conjugated systems on electron transfer in molecules, it can be stated that all three types differ in their abilities. Surprisingly, higher *E*_g_ values were recorded in the case of the terphenyl molecules. They are the least flexible once from electron-delocalization point of view, due to significant rotation angles (of approx. 30°) between the adjacent phenyls, caused by the Pauli repulsions by hydrogen atoms of the benzene units. On the other hand, the most efficient systems in terms of electron delocalization are those with alternating benzene and thiophene rings.

The effect of donor identity on the energy gap size was also tested. The comparison of HOMO–LUMO gaps for experimentally tested terthiophene derivatives 28–30 (Table 9) with those for the theoretically studied counterparts 36–38 (Table 11) demonstrates that *E*_g calc_ are smaller for the latter by 0.3–0.6 eV. Their ordering with respect to EWG identity remains however same among all groups of compounds.

## 7. Interpretation of the spectra using the CC2 calculations

The absorption spectra of all three families show systematic behaviour: in each family, the dicyanovinyl possesses the largest bathochromic shift. Table 12 shows the results of the calculations of the absorption and emission energies and oscillator strengths of selected molecules 20, 23, 28, and 33.

**Table 12:** The vertical excitation energies (Δ*E*_vert_, in eV), the wavelengths (*λ*_exc_, nm), emission energies (Δ*E*_em_, in eV), the wavelengths (*λ*_em_, nm), and oscillator strengths (*f*) calculated using ADC(2)/def2-TZVP method

	Absorption			Emission^a^		
	Δ*E*_vert_	*λ*_exc_	*f*	Δ*E*_em_	*λ*_em_	*f*
20						
S1	3.269	379	1.20	2.885	430	1.25
S2	3.619	343	0.00	—b	—	—
S3	4.041	307	0.03	3.655	339	0.00

23						
S1	2.506	495	1.18	2.232	556	1.20
S2	3.663	338	0.20	—b	—	—
S3	3.915	317	0.71	3.686	336	1.11

28						
S1	3.249	382	1.12	2.755	450	1.28
S2	3.518	352	0.00	—b	—	—
S3	4.231	293	0.01	3.736	332	0.01

33						
S1	3.604	344	0.49	2.054	604	0.00
S2	3.609	344	0.62	—b	—	—
S3	4.089	303	0.02	3.676	337	0.00
				3.600^c^	344	1.00
				3.665^d^	338	0.02

a The excited state minima were optimized using def2-SVP basis set.

b The S2 minima geometry optimizations failed due to a negligible energy gap between the S2 and S1 states.

c The S1 state at the geometry of S3 minimum.

d The S2 state at the geometry of S3 minimum.

The absorption spectra can be divided into two groups, differing by the pattern in intensity of the first and second absorption bands. The spectra of terthiophene compounds (28–30) exhibit strong first (lowest energy) absorption bands, followed by weaker ones. This finding is in accordance with the literature^59^ and also with our calculations performed for 28 with the strongest transition S0–S1 (*λ* = 382 nm, *f* = 1.12) followed by the much weaker transition of S0–S3 (*λ* = 293 nm, *f* = 0.01), in very good agreement with the experiment. On the contrary, the compounds containing the terphenyl moiety (33–35) show two absorption bands of similar intensities, the first (lower energy one) being always slightly weaker. The same is observed in calculations of 33, with the energy of S0–S1 (*λ* = 344 nm, *f* = 0.49) being weaker than the S0–S2 (*λ* = 344 nm, *f* = 0.62), even though the relative energies slightly disagree with the experiment.

The thiophene oligomers (28–30) are known to be well-conjugated as they are applied as molecular conductors.^60–62^ These molecules thus form a single conjugated system, as is seen from the inspection of molecular orbitals in Fig. 4. Consequently, terthiophene derivatives behave in accordance with the Kasha's rule, showing a single emission from the S1 state. In contrast, HOMO of the substituted terphenyl molecules (33–35) is mostly restricted to the phenyl bearing the electron-donating group and, to a smaller extent, to the middle phenyl ring. The molecule thus resembles a combination of two independent chromophores forming two fluorescing diabatic states.^63^ This behaviour is underlined by the observed dual fluorescence of anti-Kasha like character and contrasts with the one of unsubstituted terphenyl (Fig. 4, structure 39). According to the literature unsubstituted terphenyl's fluorescence follows the Kasha rule.^57^

We want to stress out here, that the “anti-Kasha” interpretation of the spectral features is only a tentative one and should be a topic of future investigations *via* time-resolved techniques. The observed spectroscopic behaviour was recorded in a single solvent (dichloromethane), at a single concentration, and without time resolution. On the other hand, the measurements were repeatable and the CC2 calculation explained the observations satisfactorily, which give us a reasonable confidence in the interpretation. In compounds 33–35 the characterization of the fluorescence from the higher energy states is complicated by the absorption to the lower states since there is a strong overlap between the S3–S0 emissions and the S0–S1 absorption. Despite the problem of self-absorption, the two emission bands are clearly recognized. In any case, further studies must be employed in order to confirm or exclude the presence of the fluorescence from high-lying excited state for substituted terphenyls. In the cases of terthiophene derivatives, only Kasha's fluorescence is observed, whereas in the terphenyls, both Kasha and anti-Kasha like emissions are clearly detected. On the other hand, the hybrid compounds containing one thiophene and two phenyl rings fluoresce strongly dependent on the substitution.

## 8. Conclusions

We have developed a synthetic approach for the preparation of three novel series of polarized aromatic conjugated D–π–A systems consisting of three aromatic systems (benzenes, thiophenes, and their mix) having the *N*,*N*-diethylamino or methyl group as a donor on one side. On the other side, the formyl, 1,1-dicyanovinyl, or 5-(2-methoxy-3,3-dimethyl-3,4-dihydro-2*H*-pyrrol-1-oxide) group was attached in the role of electron acceptor. The synthetic part reports the preparation of desired molecules and details the isolation and identification of all these organic dyes, which were not previously reported in the literature.

The series of novel molecules were fully characterized by MS, NMR, cyclic voltammetry, UV-vis, and fluorescence spectroscopy measurements. Electrochemical and optical response trends have been reproduced, analysed, and interpreted in terms of linker/substituent composition *via* DFT and wave-function-based ADC(2) calculations. It is notable that the terthiophene compounds exhibit regular fluorescence from the S1 state following Kasha's rule, whereas the terphenyl derivatives exhibit both the Kasha and anti-Kasha like fluorescence. Conversely, the fluorescence exhibited by the hybrid 2,5-diphenylthiophene compounds is contingent upon the nature of the substituent. Time-resolved methods should be employed in future studies in order to interpret unambiguously the origins of complex spectroscopic response of substituted terphenyl D–π–A systems.

## Data availability

Data supporting the article and details of syntheses, analyses, yields, physical–chemical characteristics, spectra records, and records of cyclic voltammetry measurements and Cartesian coordinates of quantum-chemically optimized structures, are enclosed in ESI.†

## Author contributions

Michaela Babejová carried out all the syntheses, analyses, and literature searches. Iveta Třísková and Libuše Trnková conducted cyclic voltammetry experiments and their evaluation. Quantum mechanical calculations were performed by Hugo Semrád and Dana Nachtigallová. Markéta Munzarová participated in the analysis and interpretation of quantum chemical results. The spectroscopic data were interpreted by Dominik Heger and Dana Nachtigallová. The project was formulated, supervised, and summarized by Milan Potáček. All authors contributed to the conceptualization and editing of the text.

## Conflicts of interest

There are no conflicts to declare.

## Supplementary Material

RA-014-D4RA02668C-s001

RA-014-D4RA02668C-s002
