# Supplementary material for: Synthesis, optical, electrochemical, and computational study of benzene/thiophene based D–π–A chromophores
Source: RSC Adv. 2024 Nov 5;14(48):35424–37. doi: 10.1039/d4ra02668c (PMC11537213; doi:10.1039/d4ra02668c)
Supplement: RA-014-D4RA02668C-s001 [file RA-014-D4RA02668C-s001.pdf]

Supplementary Information (SI) for RSC Advances.
This journal is © The Royal Society of Chemistry 2024

ELECTRONIC SUPPORTING INFORMATION

Table of contents

I. General information
II. Synthesis
III. NMR spektra
IV. Electrochemistry
V. Theoretical calculations

I. General information

All chemicals were purchased from Sigma-Aldrich and were used as received. Anhydrous solvents were dried according procedures in literature. All reaction were performed under argon atmosphere.

^{1}H and ^{13}C NMR spectra were recorded on a Bruker Advance 300 or Bruker Advance 500. All spectra were measured in $CDCl_3$. ^{1}H NMR spectra were calibrated on TMS (δ 0 ppm) or $CDCl_3$ (δ 7.27 ppm). ^{13}C NMR spectra were calibrated on $CDCl_3$ (δ 77.23 ppm). Infrared spectra were recorded on Alpha Bruker FT-IR Spectrometer (Platinum ATR). High-resolution mass spectra were collected on Agilent 6224 Accurate-Mass TOF LC-MS with dual mode (ESI/APCI) of ionization. Melting points were measured on Melting point Meter HV2.

Reactions were monitored by thin layer chromatography on TLC Silica gel 60 F_{254} purchased from Merck. Column chromatography was performed over silicagel (40 – 63 µm, 60 Å).Vacuum distillation was performed on BÜCHI Glass Oven B-580 „Kugelrohr“.

II. Synthesis

Synthesis of 4-(2-thienyl) benzaldehyde 17

To a solution of 2-bromothiophene **15** (10.0 mmol, 1.67 g) in the mixture of 1,2-dimethoxyethane (32 ml) and water (8 ml), 4-formylphenylboronic acid **16** (13.0 mmol, 2.00 g), tetrakis(triphenylphosphine) palladium (0.52 mmol, 0.60 g) a potassium phosphate (31.0 mmol, 6.54 g) were added. Reaction mixture was heated under reflux for 7.5 hours. Then the mixture was poured into brine. Product was extracted into ethyl-acetate (3 × 30 ml). Combined organic phase was dried over $MgSO_4$ and concentrated in vacuum to afford crude

product, which was purified by column chromatography (CH_2Cl_2). Yellow solid, m.p. 68-72 ºC, 1.45 g (75 %).

^{1}H NMR (300 MHz, $CDCl_3$): δ 7.12 (dd, *J* = 5.0, 3.7 Hz, 1H), 7.38 (d, *J* = 5.1 Hz, 1H), 7.44 (dd, *J* = 3.6, 0.8 Hz, 1H), 7.73 (d, *J* = 8.4 Hz, 2H), 7.86 (d, *J* = 8.4 Hz, 2H), 9.99 (s, 1H).
^{13}C NMR (75 MHz, $CDCl_3$): δ 125.3, 126.3, 127.1, 128.7, 130.7, 135.4, 140.3, 143.0, 191.6.
HRMS (APCI): calculated for $C_{11}H_2OS$ $[M+H]^+$: 189.0369, found 189.0370.
IR (ν/cm^{-1}): 818, 832, 854, 909, 958, 1054, 1110, 1171, 1214, 1262, 1289, 1310, 1351, 1390, 1424, 1529, 1565, 1602, 1662, 1699, 2734, 2796, 2822, 3075, 3108.

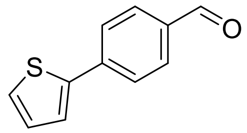

Synthesis of 4-(5-bromothiophene-2-yl) benzaldehyde 18

To a solution of 4-(thiophen-2-yl) benzaldehyde **17** (6.5 mmol, 1.20 g) in tetrahydrofuran (38 ml) at 0 ºC, *N*-bromosuccinimide (9.8 mmol) 1.74 g was added. Reaction mixture was stirred for 1.5 hours at RT. Crude product was purified by column chromatography (EtOAc:petrolether = 1:9). Yellow solid, m.p. 118-120ºC, 1.68 g (96 %).

^{1}H NMR (300 MHz, $CDCl_3$): δ 7.11 (d, *J* = 3.9 Hz, 1H), 7.23(d, *J* = 3.9 Hz, 1H), 7.68 (d, *J* = 8.3 Hz, 2H), 7.90 (d, *J* = 8.3 Hz, 2H), 10.03 (s, 1H).
^{13}C NMR (75 MHz, $CDCl_3$): δ 114.2, 125.4, 125.9, 130.7, 131.6, 139.4, 144.3, 191.5.
HRMS (APCI): calculated for $C_{11}H_7BrOS$ $[M+H]^+$: 268.9453, found 268.9454.
IR (ν/cm^{-1}): 823, 836, 945, 980, 1006, 1108, 1121, 1172, 1205, 1218, 1286, 1312, 1331, 1396, 1427, 1499, 1507, 1522, 1541, 1563, 1603, 1668, 1692, 2366, 2756, 2849, 3082, 3092.

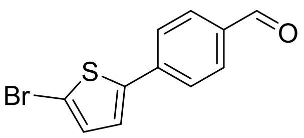

Synthesis of 4-{5-[4-(*N,N*-diethylamino)phenyl]thiophene-2-yl} benzaldehyde 20

To a solution of 4-(5-bromthiophene-2-yl) benzaldehyde **18** (5.8 mmol, 1.56 g) in the mixture of 1,2-dimethoxyethane (40 ml) and water (8 ml), 4-(N,N-diethylamino)phenylboronic acid **19** (7.6 mmol, 1.47 g), [1,1´-bis(diphenylphosphino)ferrocene]dichloropalladium (0.46 mmol, 0.34 g) a potassium phosphate (18.0 mmol, 3.73 g) were added. Reaction mixture was heated

under reflux for 2 hours. Then mixture was poured into brine. Product was extracted into ethyl acetate (3 × 30 ml). Combined organic phase was dried over $MgSO_4$ and concentrated in vacuum to afford crude product, which was purified by column chromatography (EtOAc:petrolether = 1:9). Orange solid, m.p. 175-180 ºC, 1.51 g (77 %).

^{1}H NMR (300 MHz, $CDCl_3$): δ 1.20 (t, J = 7.1 Hz, 6H), 3.40 (q, J = 7.1 Hz, 4H), 6.69 (d, J = 8.9 Hz, 2H), 7.15 (d, J = 3.8 Hz, 1H), 7.40 (d, J = 3.8 Hz, 1H), 7.50 (d, J = 8.8 Hz, 2H), 7.74 (d, J = 8.3 Hz, 2H), 7.86 (d, J = 8.4 Hz, 2H), 9.98 (s, 1H).
^{13}C NMR (75 MHz, $CDCl_3$): δ 12.8, 44.7, 111.9, 121.3, 122.0, 125.5, 126.3, 127.3, 130.7, 134.8, 139.2, 140.8, 147.7, 147.9, 191.6.
HRMS (APCI): calculated for $C_{21}H_{21}NOS$ $[M+H]^+$: 336.1417, found 336.1417.
IR (ν/cm^{-1}): 830, 938, 1075, 1110, 1170, 1197, 1219, 1271, 1358, 1377, 1401, 1419, 1426, 1451, 1468, 1497, 1521, 1542, 1561, 1600, 1695, 2732, 2819, 2872, 2896, 2933, 2974.

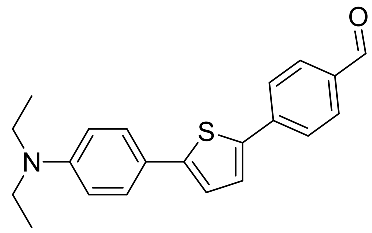

Synthesis of *(E)*-5-{4-[5-(4-(*N,N*-diethylamino) phenyl) thiophene-2-yl] styryl}-2-methoxy-3,3-dimethyl-3,4-dihydro-*2H*-pyrrol-1-oxide 21

To a solution of allenyloxim **3** (0.58 mmol, 0.074 g) and potassium hydroxide (0.90 mmol, 0.052 g) in methanol (5 ml), aldehyde **20** (0.30 mmol, 0.10 g) was added. Reaction mixture was heated under reflux for 2 days. The solvent was removed in vacuum. Residue was dissolved in dichloromethane, washed with water and brine and dried over $MgSO_4$. Solvent was removed in vacuum. Crude product was purified by column chromatography (EtOAc:CH_2Cl_2 = 3:1). Red solid, m.p. 141-145 ºC, 0.038 g (27 %).

^{1}H NMR (300 MHz, $CDCl_3$): δ 1.11 – 1.24 (m, 12H), 2.56 (d, J = 16.3 Hz, 1H), 2.74 (d, J = 16.4 Hz, 1H), 3.38 (q, J = 7.0 Hz, 4H), 3.87 (s, 3H), 4.58 (s, 1H), 6.67 (d, J = 8.9 Hz, 2H), 6.91 (dd, J = 16.6, 3.9 Hz, 1H), 7.10 (d, J = 3.8 Hz, 1H), 7.29 (d, J = 3.8 Hz, 1H), 7.35 – 7.43 (m, 1H), 7.44 – 7.54 (m, 4H), 7.59 (d, J = 8.3 Hz, 2H).
^{13}C NMR (126 MHz, $CDCl_3$): δ 12.7, 21.9, 27.2, 37.1, 40.9, 44.4, 60.9, 108.1, 111.8, 115.4, 121.6, 124.5, 125.5, 127.0, 128.0, 134.6, 135.6, 136.5, 140.2, 143.2, 145.7, 147.5.

HRMS (APCI): calculated for $C_{29}H_{34}N_2O_2S$ $[M+H]^+$: 475.2414, found 475.2412.
IR (ν/cm^{-1}): 818, 861, 939, 969, 1004, 1075, 1110, 1140, 1155, 1196, 1220, 1269, 1299, 1357, 1373, 1398, 1418, 1453, 1499, 1535, 1556, 1595, 1606, 2851, 2871, 2930, 2968, 3042.

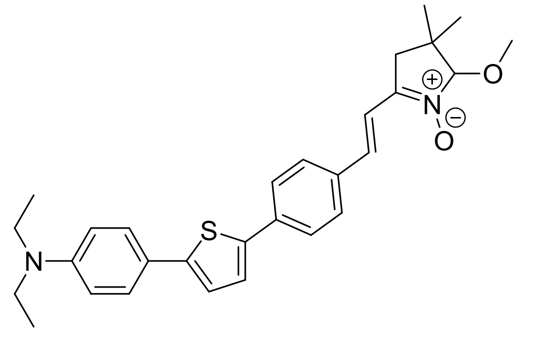

Synthesis of 2-{4-[5-(4-(*N,N*-diethylaminophenyl)] thiophene-2-yl} benzyliden malononitrile 23

To a solution of malononitrile **22** (0.36 mmol, 0.024 g) and piperidine (0.36 mmol, 0.030 g) in ethanol (5 ml), aldehyde **20** (0.36 mmol, 0.12 g) was added. Reaction mixture was stirred for 8 hours at RT. The solvent was removed in vacuum. Residue was dissolved in dichloromethane, washed with water and brine and dried over $MgSO_4$. Solvent was removed in vacuum. Crude product was purified by column chromatography (CH_2Cl_2) and crystallization from hexane. Brown solid, m.p. 220-223 ºC, 0.11 g (77 %).

^{1}H NMR (300 MHz, $CDCl_3$): δ 1.23 (t, J = 7.0 Hz, 6H), 3.43 (q, J = 7,0 Hz, 4H), 6.71 (d, J = 8.5 Hz, 2H), 7.19 (d, J = 4.0 Hz, 1H), 7.48 (d, J = 3.7 Hz, 1H), 7.53 (d, J = 8.7 Hz, 2H), 7.70 (s, 1H), 7.74 (d, J = 8.3, 2H), 7.93 (d, J = 8,4 Hz, 2H).
^{13}C NMR (126 MHz, $CDCl_3$): δ 12.6, 44.5, 80.3, 111.7, 113.2, 114.3, 120.8, 122.0, 125.4, 127.0, 127.1, 129.0, 131.7, 138.3, 140.8, 147.9, 148.7, 158.5.
HRMS (APCI): calculated for $C_{24}H_{21}N_3S$ $[M+H]^+$: 384.1529, found 384.1528.
IR (ν/cm^{-1}): 807, 824, 856, 925, 938, 949, 965, 1006, 1073, 1092, 1159, 1193, 1232, 1267, 1304, 1318, 1351, 1373, 1401, 1444, 1466, 1499, 1536, 1577, 1600, 1684, 1700, 1717, 1734, 1742, 2225, 2364, 2896, 2925, 2938, 2975, 3023.

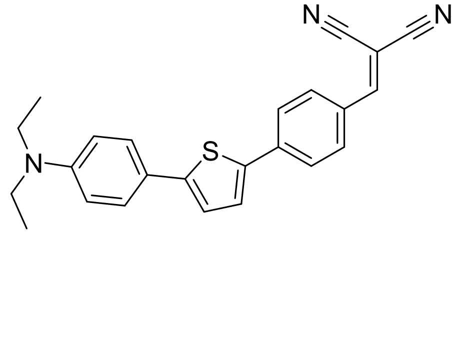

Synthesis of (2,2′-bithiophene)-5-carbaldehyde 25

To a solution of 5-bromothiophene-2-carbaldehyde **10** (5.2 mmol, 1.00 g) in the mixture of 1,2-dimethoxyethane (30 ml) and water (6 ml), 2-thienylboronic acid **24** (6.8 mmol, 0.87 g), [1,1′-bis(diphenylphosphino)ferrocene]dichlorpalladium (0.26 mmol, 0.19 g) and potassium phosphate (16.0 mmol, 3.33 g) were added. Reaction mixture was heated under reflux for 24 hours. Then mixture was poured into brine. Product was extracted into ethyl acetate. Combined organic phase was dried over $MgSO_4$ and concentrated in vacuum to afford crude product, which was purified by column chromatography (CH_2Cl_2:hexane = 3:1). Orange solid, m.p. 53-57 ºC, 0.71 g (70 %).

^{1}H NMR (300 MHz, $CDCl_3$): δ 7.05 (dd, *J* = 4.9, 3.6 Hz, 1H), 7.22 (d, *J* = 3.9 Hz. 1H), 7.34 (m, 2H), 7.64 (d, *J* = 3.9 Hz, 1H), 9.84 (s, 1H).

^{13}C NMR (75 MHz, $CDCl_3$): δ 124.5, 126.4, 127.3, 128.6, 136.3, 137.5, 142.0, 147.4, 182.7.

HRMS (APCI): calculated for $C_9H_6OS_2$ $[M+H]^+$: 194.9933, found 194.9933.

IR (ν/cm^{-1}): 803, 843, 893, 1050, 1080, 1162, 1201, 1228, 1299, 1314, 1382, 1399, 1415, 1421, 1449, 1508, 1546, 1657, 2811, 2838, 3088, 3105.

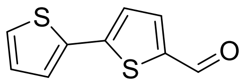

Synthesis of 5′-bromo-(2,2′-bithiophene)-5-carbaldehyde 26

To a solution of aldehyde **25** (2.7 mmol, 0.53 g) in tetrahydrofuran (20 ml) at 0 ºC, *N*-bromosuccinimide (4.4 mmol, 0.78 g) was added. Reaction mixture was stirred for 3 hours at RT. Crude product was purified by column chromatography (CH_2Cl_2:hexane = 2:1).Yellow solid, m.p. 145-149ºC, 0.67 g (90 %).

^{1}H NMR (300 MHz, $CDCl_3$): δ 7.06 (d, *J* = 3.9 Hz, 1H), 7.13 (d, *J* = 3.9 Hz. 1H), 7.21 (d, *J* = 3.9 Hz, 1H), 7.68 (d, *J* = 4.0 Hz, 1H), 9.89 (s, 1H).

^{13}C NMR (75 MHz, $CDCl_3$): δ 114.4, 124.6, 126.4, 131.4, 137.3, 137.7, 142.3, 146.0, 182.6.

HRMS (APCI): calculated for $C_9H_5BrOS_2$ $[M+H]^+$: 274.9016, found 274.9014.

IR (ν/cm^{-1}): 880, 974, 1051, 1059, 1193, 1233, 1377, 1423, 1458, 1510, 1626, 1657, 2807, 2833, 3096.

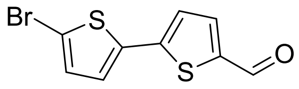

Synthesis of 5″-methyl-[2,2′:5′,2″-terthiophene]-5-carbaldehyde 28

To a solution of 5´-bromo-(2,2´-bithiophen)-5-carbaldehyde **26** (1.5 mmol, 0.40 g) in 1,2-dimethoxyethane (15 ml), 5-methylthiophene-2-boronic acid pinacol ester **27** (2.2 mmol, 0.49 g), [1,1´-bis(diphenylphosphino)ferrocene]dichlorpalladium (0.073 mmol, 0.054 g) and 2M solution of potassium carbonate (4.4 mmol, 2.2 ml) were added. Reaction mixture was heated under reflux for 5.5 hours. Then mixture was poured into brine. Product was extracted into dichloromethane. Combined organic phase was dried over $MgSO_4$ and concentrated in vacuum to afford crude product, which was purified by column chromatography (CH_2Cl_2:hexane = 2:1) and crystallization from diethylether. Orange solid, m.p. 157-160 ºC, 0.30 g (70 %).

^{1}H NMR (300 MHz, $CDCl_3$): δ 2.49 (s, 3H), 6.68 – 6.74 (m, 1H), 7.04 (t, J = 3.7 Hz, 2H), 7.23 (d, J = 4.0 Hz, 1H), 7.26 (d, J = 3.8 Hz, 1H), 7.67 (d, J = 4.0 Hz, 1H), 9.85 (s, 1H).

^{13}C NMR (75 MHz, $CDCl_3$): δ 15.4, 123.8, 123.9, 124.5, 126.3, 126.9, 133.8, 134.1, 137.3, 139.7, 140.5, 141.5, 147.0, 182.3.

HRMS (APCI): calculated for $C_{14}H_{10}OS_3$ $[M+H]^+$: 290.9967, found 290.9967

IR (ν/cm^{-1}): 814, 859, 912, 1051, 1066, 1158, 1199, 1223, 1231, 1251, 1383, 1440, 1450, 1479, 1507, 1558, 1618, 1663, 1712, 2807, 2826, 2835, 2918, 3061, 3078.

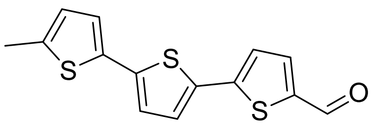

Synthesis of *(E)*-2-methoxy-3,3-dimethyl-5-{2-[5″-methyl-[2,2′:5′,2″-terthiophene]-5-yl) vinyl}-3,4-dihydro-*2H*-pyrrol-1-oxide 29

To a solution of allenyloxim **3** (0.17 mmol, 0.022 g) and potassium hydroxide (0.19 mmol, 0.011 g) in methanol (3 ml), aldehyde **28** (0.086 mmol, 0.025 g) was added. Reaction mixture was heated under reflux for 2 days. The solvent was removed in vacuum. Residue was dissolved in dichloromethane, washed with water and brine and dried over $MgSO_4$. Solvent was removed in vacuum. Crude product was purified by column chromatography (Et_2O:CH_3OH = 12:1) and crystallization from the mixture hexane:CH_2Cl_2 = 2:1. Orange solid, m.p. 177-179 ºC, 0.022 g (60 %).

^{1}H NMR (500 MHz, $CDCl_3$): δ 1.13 (s, 3H), 1.20 (s, 3H), 2.43 – 2. 53 (m, 4 H), 2.69 (d, *J* = 16.2 Hz, 1H), 3.86 (s, 3H), 4.49 (s, 1H), 6.66 – 6.69 (m, 1H), 6.96 – 7.01 (m, 2H), 7.02 – 7.10 (m, 5H).

^{13}C NMR (126 MHz, $CDCl_3$): δ 15.4, 21.9, 27.2, 37.1, 41.0, 60.9, 108.0, 115.0, 123.9. 124.1, 125.0, 126.1, 129.0, 129.9, 134.6, 135.0, 137.7, 139.0, 139.8, 140.6, 142.3.

HRMS (APCI): calculated for $C_{22}H_{23}NO_2S_3$ $[M+H]^+$: 430.0964, found 430.0961.

IR (ν/cm^{-1}): 951, 1005, 1128, 1396, 1419, 1437, 1458, 1489, 1507, 1521, 1541, 1569, 1617, 1648, 1670, 1699, 1717, 1733, 1772, 1918, 1965, 2006, 2037, 2141, 2331, 2364, 3567, 3617, 3648, 3689, 3711, 3745, 3801, 3820, 3838, 3853, 3870, 3882, 3910.

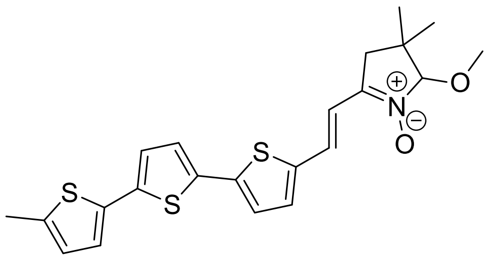

Synthesis of 2-(5′′-methyl-[2,2′:5′,2′′-terthiophene]-5-yl) methylenmalononitrile 30

To a solution of malononitrile **22** (0.17 mmol, 0.011 g) and piperidine (0.17 mmol, 0.015 g) in ethanol (2 ml), aldehyde **28** (0.17 mmol, 0.050 g) was added. Reaction mixture was stirred for 2 hours at RT. The solvent was removed in vacuum. Residue was dissolved in dichloromethane, washed with water and brine and dried over $MgSO_4$. Solvent was removed in vacuum. Crude product was purified by column chromatography (CH_2Cl_2:hexane = 3:1) and crystallization from the mixture of CH_2Cl_2:hexane = 1:2. Red solid, m.p. 192-195 ºC, 0.042 g (75 %).

^{1}H NMR (300 MHz, $CDCl_3$): δ 2.53 (s, 3H), 6.74 (dd, *J* = 3.6 Hz, 1.1 Hz, 1H), 7.04 – 7.12 (m, 2H), 7.26 (d, *J* = 4.1 Hz, 1H), 7.35 (d, *J* = 3.9 Hz, 1H), 7.65 (d, *J* = 4.5 Hz, 1H), 7.76 (s, 1H).

^{13}C NMR (126 MHz, $CDCl_3$): δ 15.5, 75.8, 113.5, 114.3, 124.1, 124.2, 125.0, 126.5, 128.1, 132.9, 133.3, 133.8, 140.1, 141.1, 141.3, 149.3, 149.9.

HRMS (APCI): calculated for $C_{17}H_{10}N_2S_3$ $[M+H]^+$: 339.0079, found 339.0078.

IR (ν/cm^{-1}): 1396, 1419, 1457, 1489, 1507, 1534, 1558, 1575, 1624, 1647, 1670, 1699, 1717, 1741, 1772, 1829, 1868, 2035, 2166, 2331, 2362, 3567, 3608, 3628, 3648, 3675, 3689, 3711, 3735, 3750, 3757, 3801, 3820, 3853, 3870, 3881, 3902.

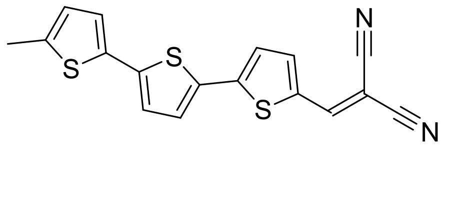

Synthesis of 4′-bromo-(1,1′-biphenyl)-4-*N,N*-diethylamine 32

To a solution of 1-bromo-4-iodobenzene **31** (5.3 mmol, 1.50 g) in the mixture of 1,2-dimethoxyethane (30 ml) and water (7.5 ml), 4-(diethylamino)phenylboronic acid **19** (6.9 mmol, 1.33 g), tetrakis(triphenylphosphine) palladium (0.32 mmol, 0.37 g) and potassium phosphate (16.0 mmol, 3.38 g) were added. Reaction mixture was heated under reflux for 24 hours. Then mixture was poured into brine. Product was extracted into ethyl acetate. Combined organic phase was dried over $MgSO_4$ and concentrated in vacuum to afford crude product, which was purified by column chromatography (EtOAc:hexan = 2:5). White solid, m.p. 123-128 °C, 1.38 g (86 %).

^{1}H NMR (300 MHz, $CDCl_3$): δ 1.19 (t, *J* = 7.1 Hz, 6H), 3.39 (q, *J* = 7.1 Hz, 1H), 6.70 – 6.75 (m, 2H), 7.37 – 7.45 (m, 4H), 7.45 – 7.51 (m, 2H).

^{13}C NMR (75 MHz, $CDCl_3$): δ 12.6, 44.4, 112.0, 119.7, 126.6, 127.6, 127.7, 131.6, 140.2, 147.4.

HRMS (APCI): calculated for $C_{16}H_{18}BrN$ $[M+H]^+$: 304.0695, found 304.0694.

IR (ν/cm^{-1}): 807, 837, 908, 991, 1004, 1074, 1093, 1154, 1199, 1214, 1265, 1346, 1359, 1374, 1401, 1429, 1446, 1485, 1526, 1608, 2868, 2886, 2930, 2973.

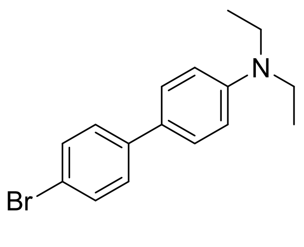

Synthesis of 4″-(*N,N*-diethylamino)-[1,1′:4′,1″-terphenyl]-4-carbaldehyde 33

To a solution of 4′-bromo-(1,1′-biphenyl)-4-*N,*N-diethylamine **32** (4.4 mmol, 1.33 g) in the mixture of 1,2-dimethoxyethane (30 ml) and water (7 ml), 4-formylphenylboronic acid **16** (6.1 mmol, 1.52 g), [1,1′-bis(diphenylphosphino)ferrocene]dichlorpalladium (0.22 mmol, 0.16 g) and potassium carbonate (13.0 mmol, 1.80 g) were added. Reaction mixture was heated under reflux for 24 hours. Then mixture was poured into brine. Product was extracted into ethyl acetate. Organic phase was dried over $MgSO_4$ and concentrated in vacuum to afford crude

product, which was purified by column chromatography (EtOAc:hexan = 1:4) and crystallization from ethyl acetate. Yellow solid, m.p. 232-235 ºC, 1.38 g (86 %).

^{1}H NMR (300 MHz, $CDCl_3$): δ 1.21 (t, J = 7.0 Hz, 6H), 3.41 (q, J = 7.0 Hz, 4H), 6.77 (d, J = 8.8 Hz, 2H), 7.54 (d, J = 8.8 Hz, 2H), 7.67 (s, 4H), 7.79 (d, J = 8.2 Hz, 2H), 7.95 (d, J = 8.2 Hz, 2H), 10.05 (s, 1H).

^{13}C NMR (75 MHz, $CDCl_3$): δ 12.7, 44.4, 112.0, 126.5, 126.9, 127.3, 127.6, 127.9, 130.3, 135.0, 136.8, 141.6, 147.0, 147.5, 191.9.

HRMS (APCI): calculated for $C_{23}H_{23}NO$ $[M+H]^+$: 330.1852, found 330.1851.

IR (ν/cm^{-1}): 807, 828, 841, 1008, 1073,1095, 1159, 1173, 1198, 1213, 1272,1310, 1327, 1360, 1403, 1449, 1470, 1490, 1518, 1539, 1574, 1595, 1608, 1697, 2729, 2813, 2900, 2934, 2975.

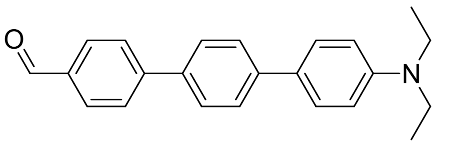

Synthesis of *(E)*-5-{2-[4″-(*N,N*-diethylamino)-[1,1′:4′,1″-terphenyl]-4-yl]-vinyl}-2-methoxy-3,3-dimethyl-3,4-dihydro-*2H*-pyrrol-1-oxide 34

To a solution of allenyloxim **3** (0.61 mmol, 0.08 g) and potassium hydroxide (0.67 mmol, 0.04 g) in methanol (5 ml), aldehyde **33** (0.30 mmol, 0.10 g) was added. Reaction mixture was heated under reflux for 2 days. The solvent was removed in vacuum. Residue was dissolved in dichloromethane, washed with water and brine and dried over $MgSO_4$. Solvent was removed in vacuum. Crude product was purified by column chromatography (Et_2O:CH_3OH = 12:1) and crystallization from the mixture hexane:CH_2Cl_2 = 2:1. Orange solid, m.p. 215-220 ºC, 0.015 g (11 %).

^{1}H NMR (500 MHz, $CDCl_3$): δ 1.13 – 1.24 (m, 12H), 2.57 (d, *J* = 12.2 Hz, 1H), 2.75 (d, *J* = 16.2 Hz, 1H), 3.41 (q, *J* = 7.0 Hz, 4H), 3.88 (s, 3H), 4.53 (s, 1H), 6.76 (d, *J* = 8.9 Hz, 2H), 6.94 (d, *J* = 16.5 Hz, 1H), 7.42 (d, *J* = 16.5 Hz, 1H), 7.53 (d, *J* = 8.8 Hz, 2H), 7.56 – 7.69 (m, 8H).

^{13}C NMR (126 MHz, $CDCl_3$): δ 12.7, 21.9, 27.3, 37.0, 40.9, 44.4, 60.9, 108.1, 112.0, 115.8, 126.4, 127.1, 127.2, 127.8, 127.9, 134.8, 136.5, 137.5, 140.7, 141.7, 143.0, 147.4.

HRMS (APCI): calculated for $C_{31}H_{36}N_2O_2$ $[M+H]^+$: 469.2850, found 469.2849.

IR (ν/cm^{-1}): 1457, 1507, 1541, 1647, 1684, 1717, 1868, 1898, 1920, 1945, 1966, 1990, 2006, 2028, 2071, 2111, 2134, 2164, 2201, 2252, 2272, 2323, 2345, 2363, 3610, 3649, 3711, 3750, 3800, 3821, 3853.

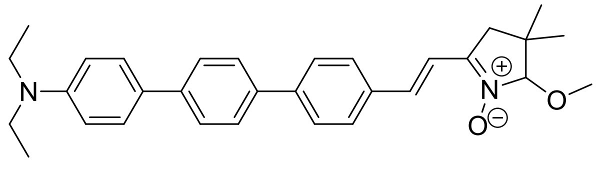

Synthesis of 2-{(4″-(*N,N*-diethylamino)-[1,1′:4′,1″-terphenyl]-4-yl} methylen malononitrile 35

To a solution of malononitrile **22** (0.30 mmol, 0.020 g) and triethylamine (0.30 mmol, 0.031 g) in dichloromethane (5 ml), aldehyde **33** (0.30 mmol, 0.10 g) was added. Reaction mixture was stirred for 5 hours at RT. The solvent was removed in vacuum. Residue was dissolved in dichloromethane, washed with water and brine and dried over $MgSO_4$. Solvent was removed in vacuum. Crude product was purified by column chromatography (CH_2Cl_2) and crystallization from ethyl acetate. Red solid, m.p. 187-191 ºC, 0.089 g (78 %).

^{1}H NMR (300 MHz, $CDCl_3$): δ 1.21 (t, J = 7.0Hz, 6H), 3.41 (q, J = 7.1 Hz, 4H), 6.76 (d, J = 8.9 Hz, 2H), 7.54 (d, J = 8.9 Hz, 2H), 7.67 (s, 4H), 7.75 (s, 1H), 7.79 (d, J = 8.5 Hz, 2H), 7.98 (d, J = 8.4 Hz, 2H).

^{13}C NMR (75 MHz, $CDCl_3$): δ 12.9, 44.6, 81.7, 112.2, 113.2, 114.3, 126.8, 127.7, 127.8, 128.1, 129.7, 131.7, 136.1, 142.3, 147.4, 147.8, 159.4.

HRMS (APCI): calculated for $C_{26}H_{23}N_3$ $[M+H]^+$: 378.1965, found 378.1966.

IR (ν/cm^{-1}): 809, 911, 1006, 1058, 1157, 1200, 1270, 1357, 1404, 1292, 1516, 1536, 1579, 2227, 2974.

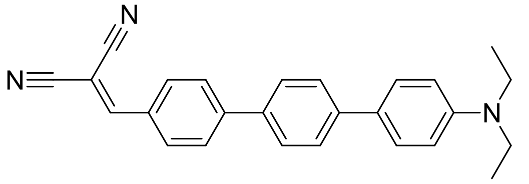

III. NMR spectra

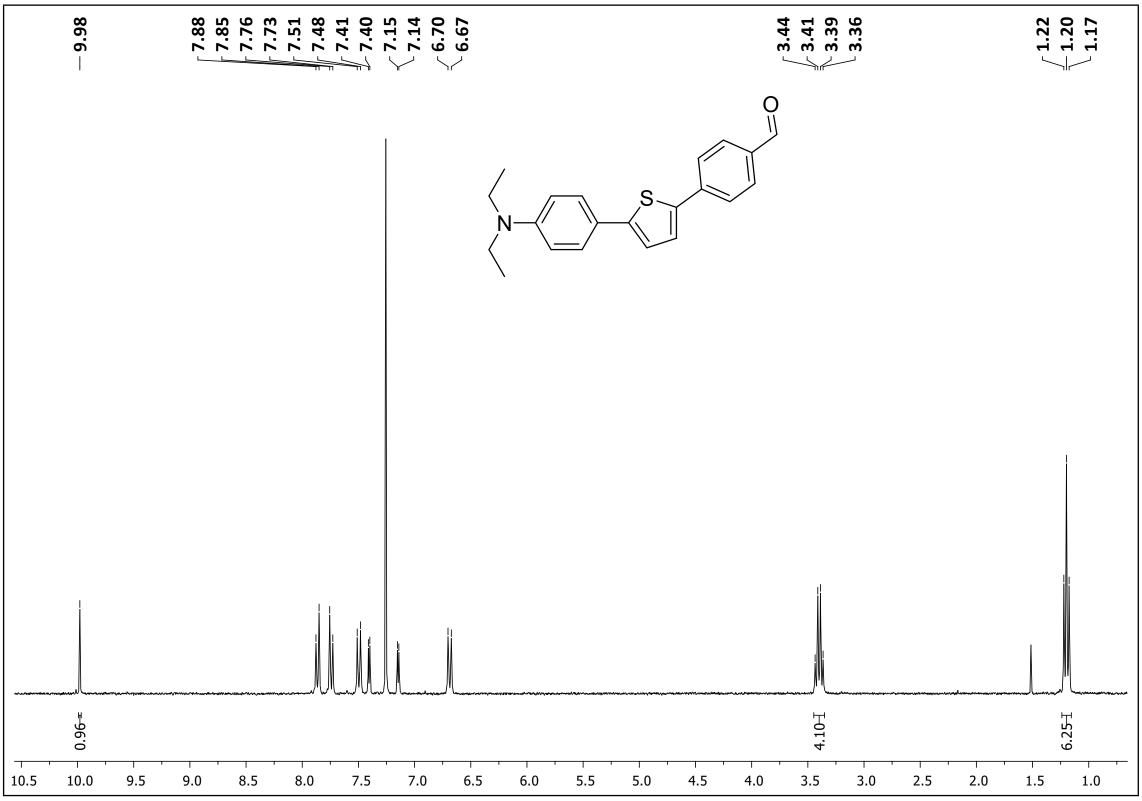

^{1}H NMR spectrum of compound **20**

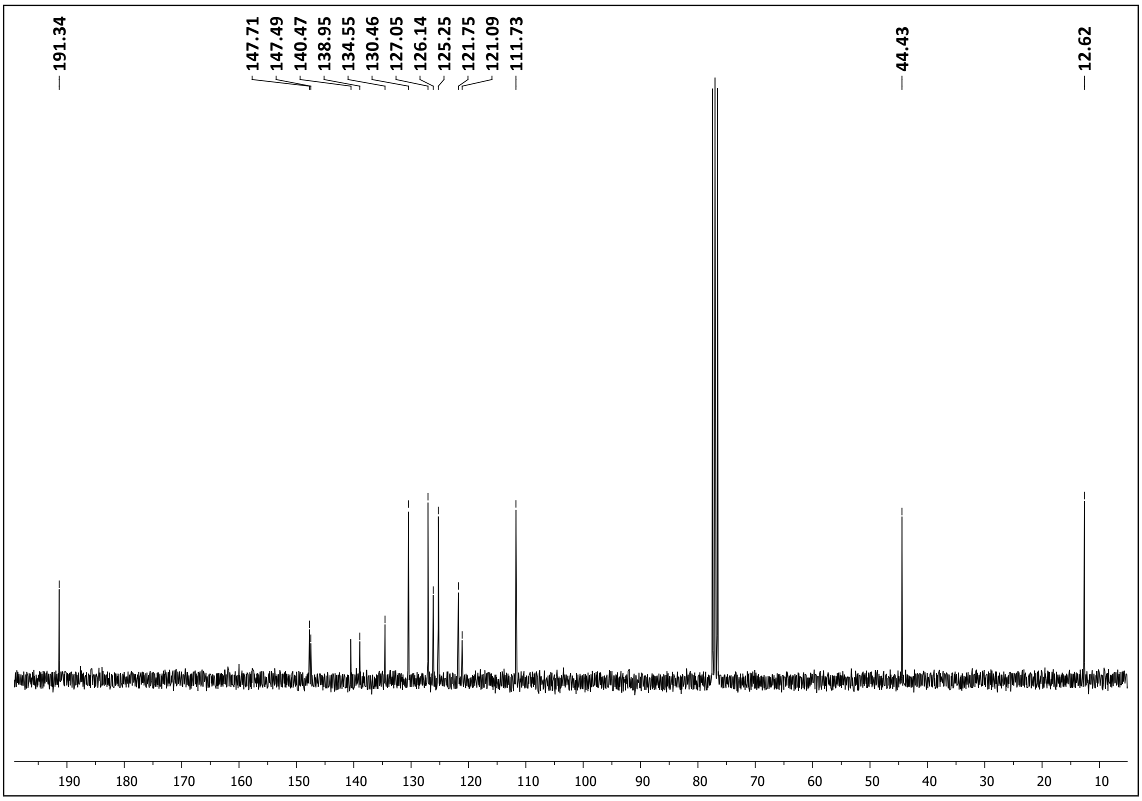

^{13}C NMR spectrum of compound **20**

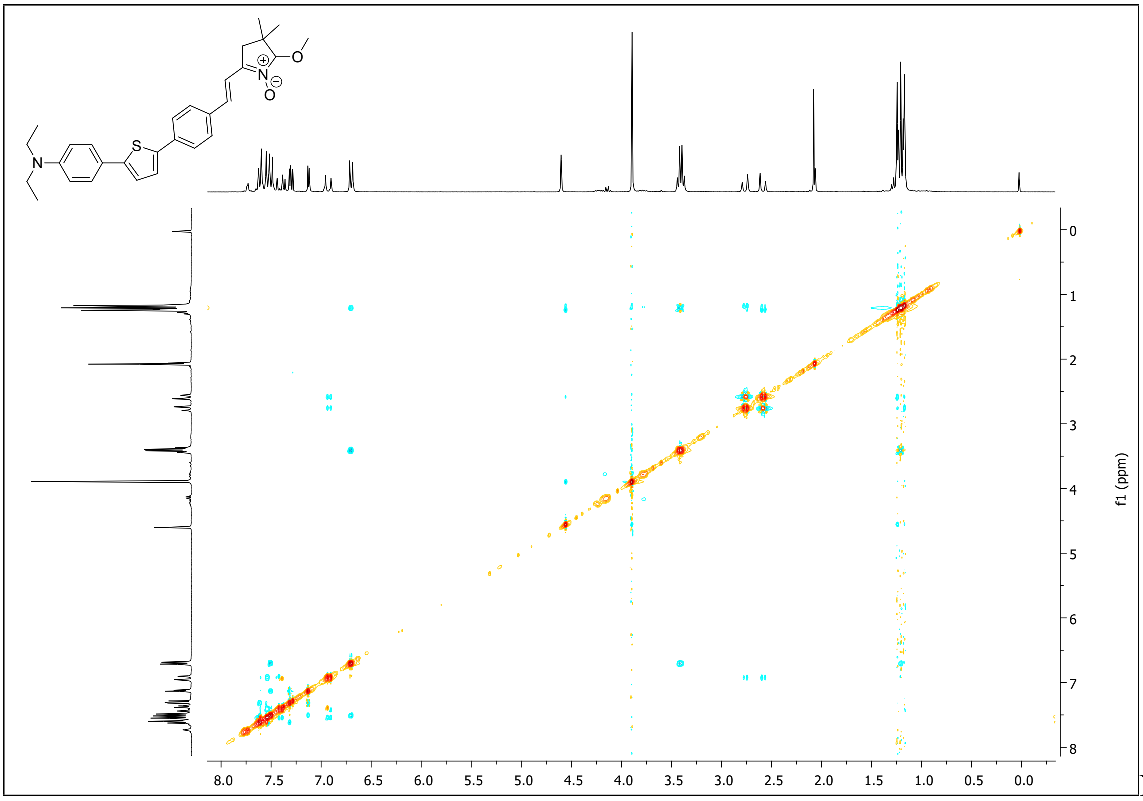

NOESY spectrum of compound **21**

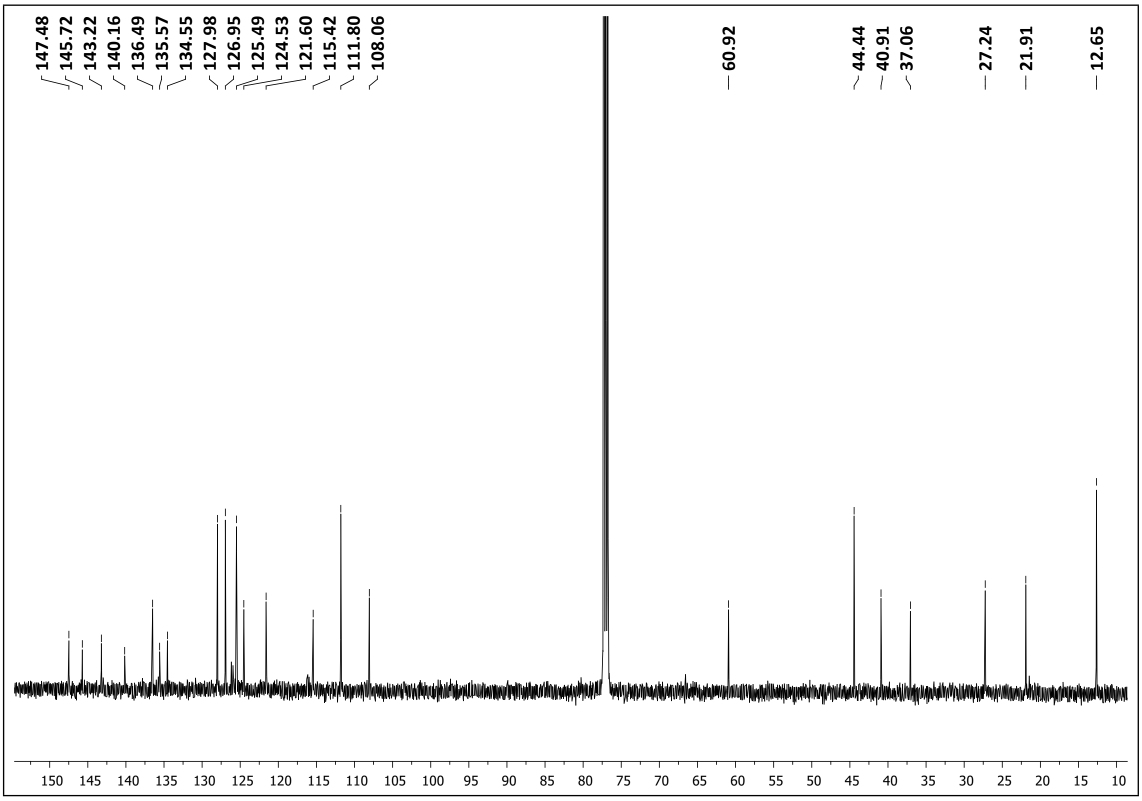

^{13}C NMR spectrum of compound **21**

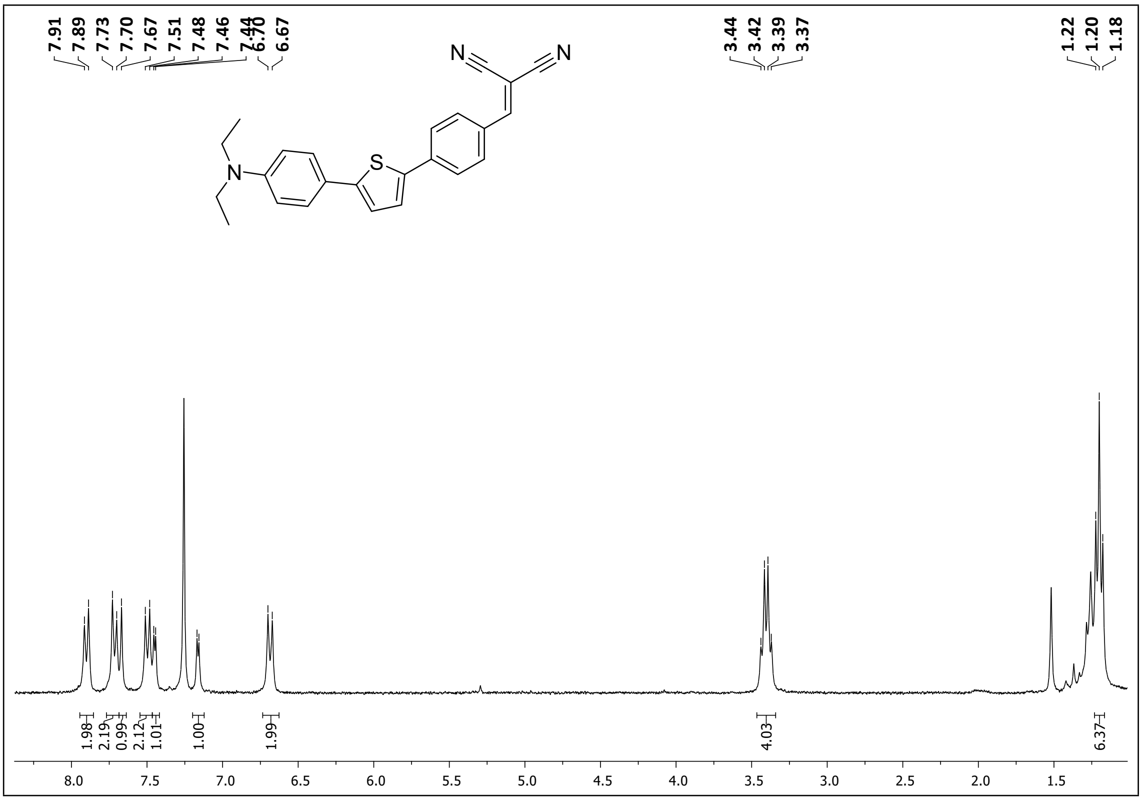

^{1}H NMR spectrum of compound **23**

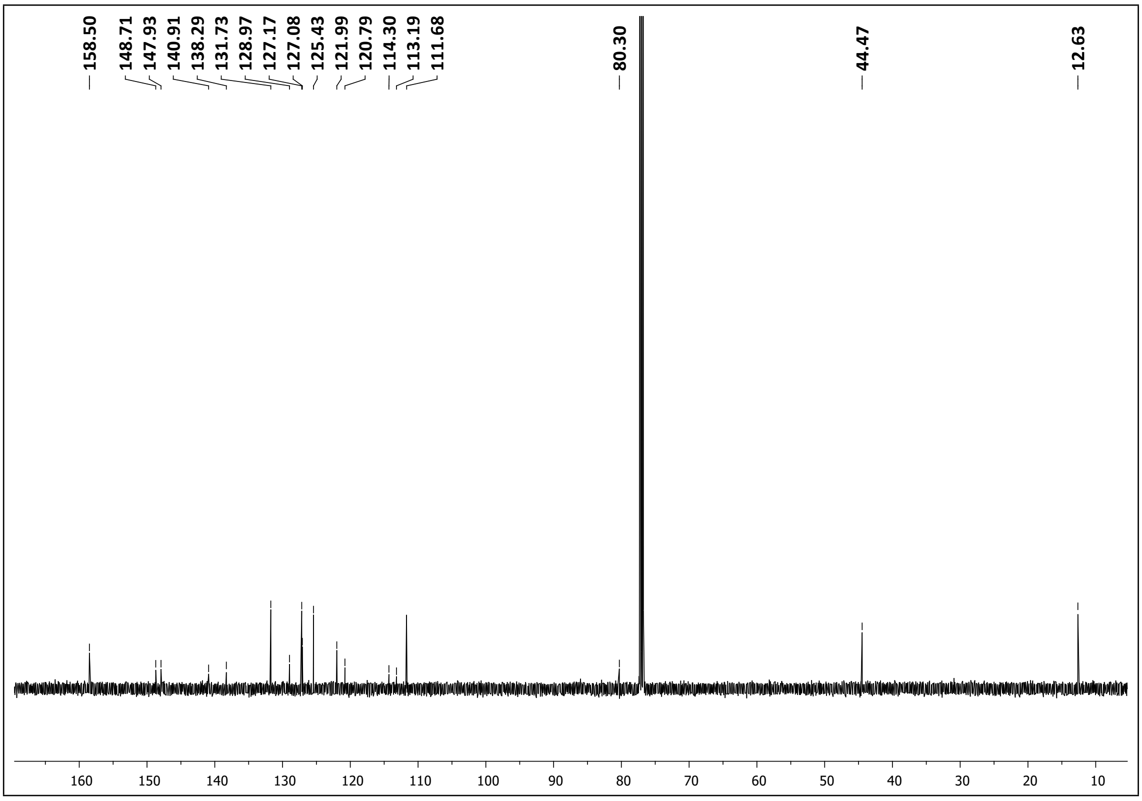

^{13}C NMR spectrum of compound **23**

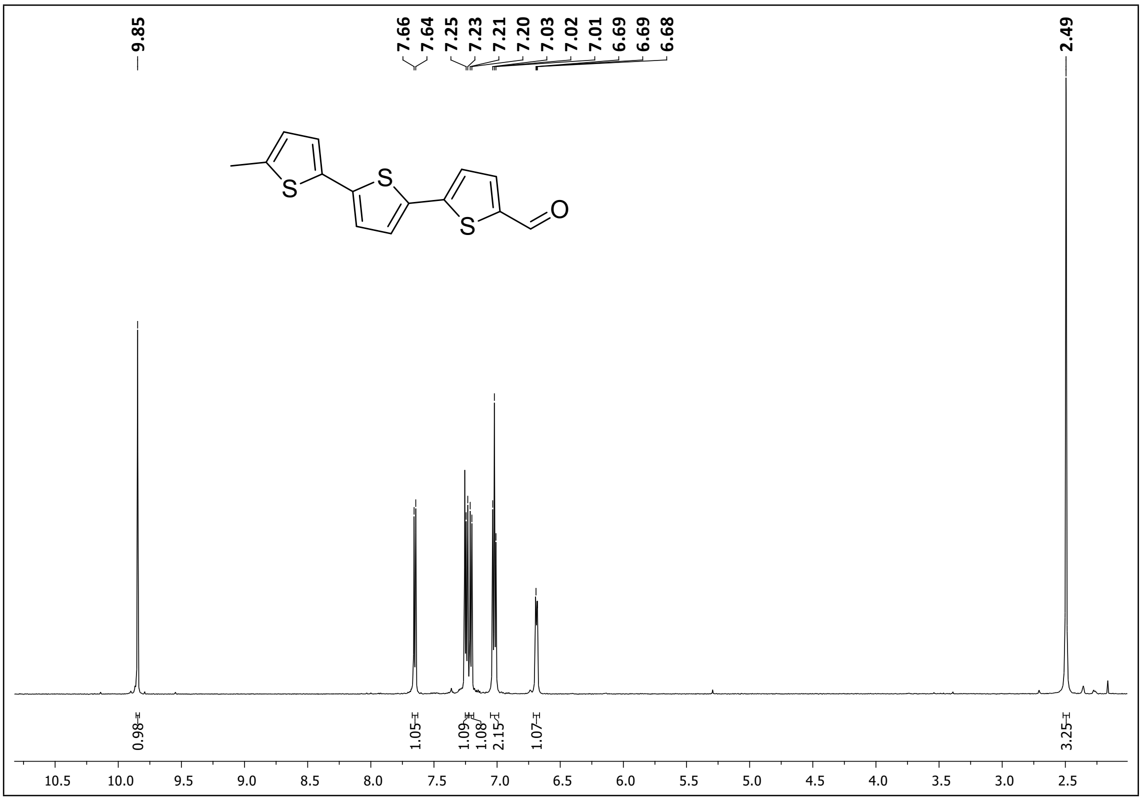

^{1}H NMR spectrum of compound **28**

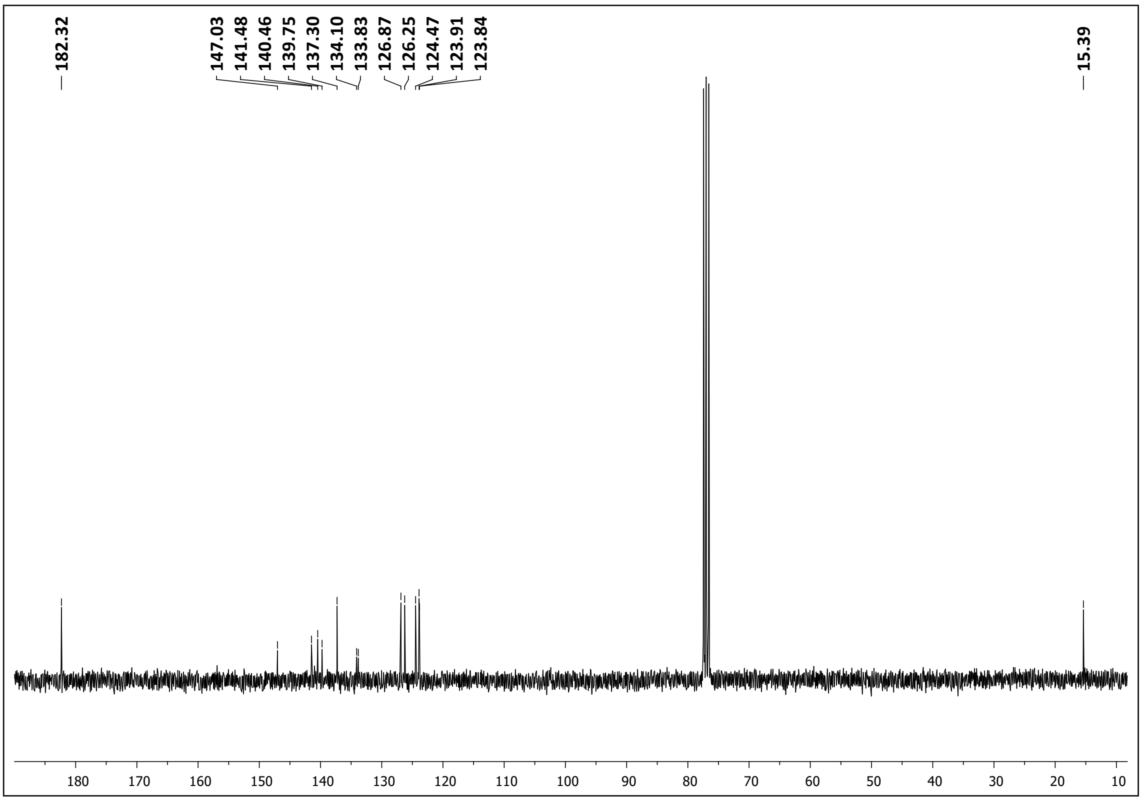

^{13}C NMR spectrum of compound **28**

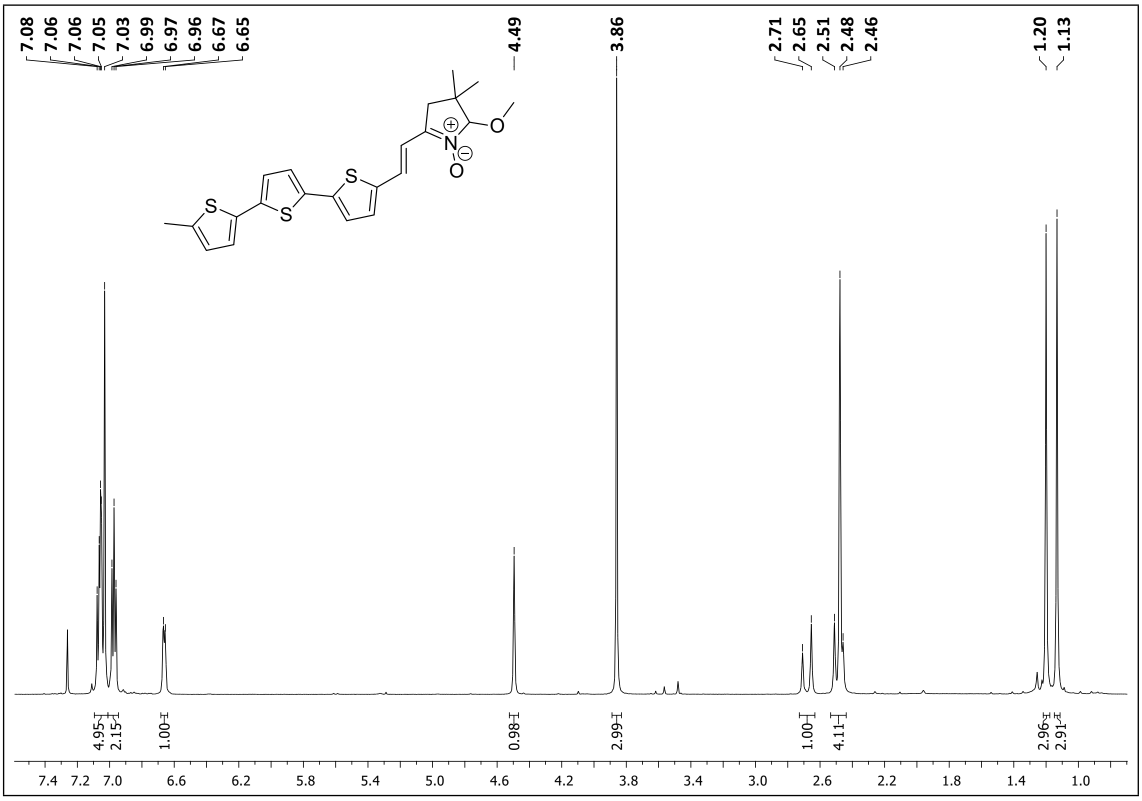

^{1}H NMR spectrum of compound **29**

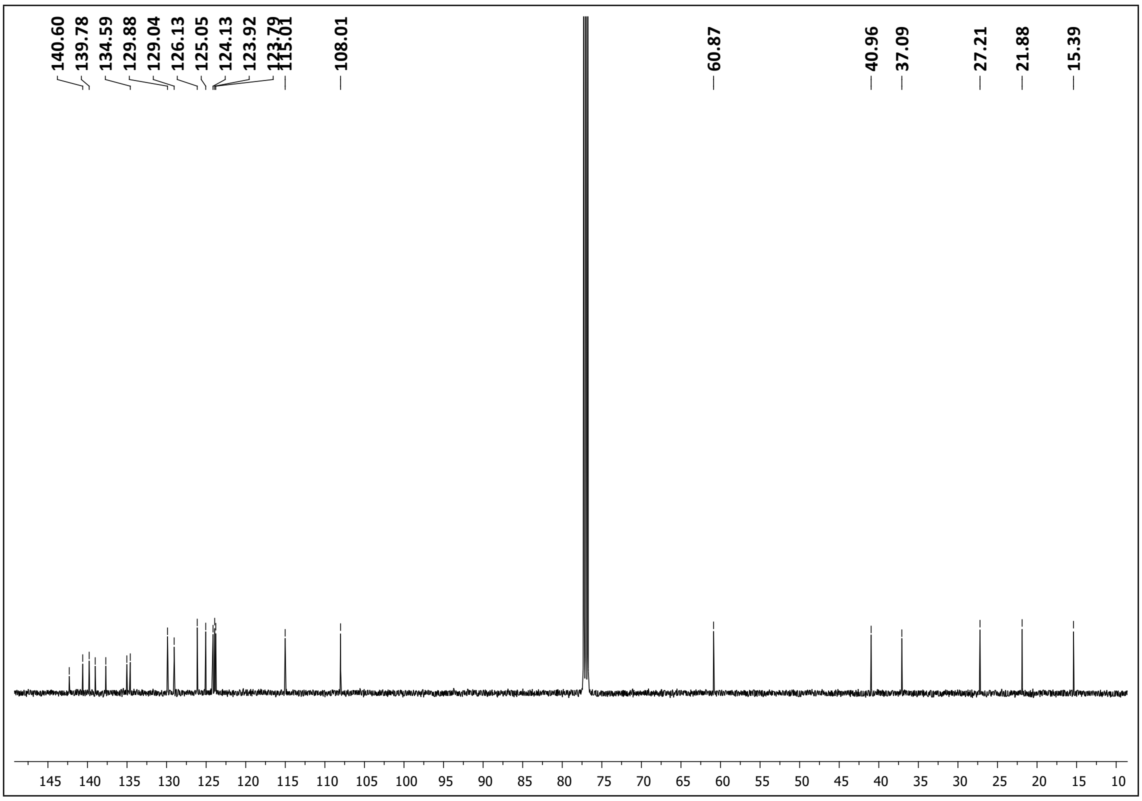

^{13}C NMR spectrum of compound **29**

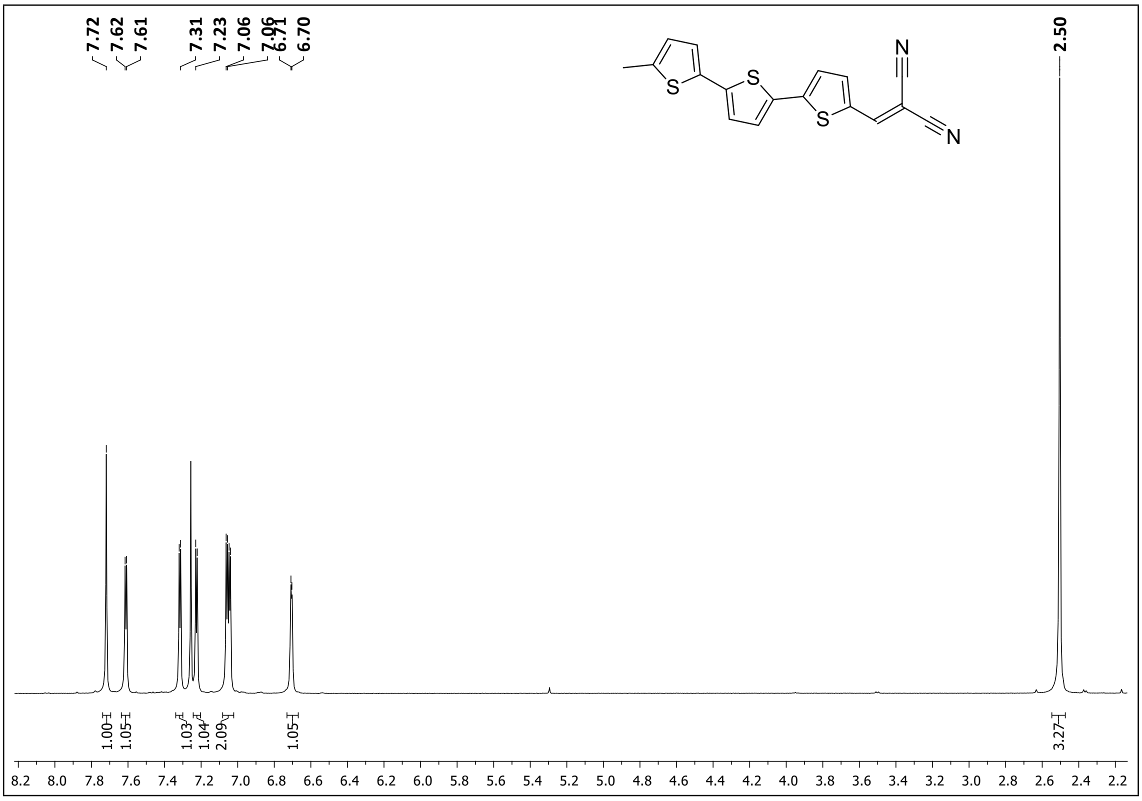

^{1}H NMR spectrum of compound **30**

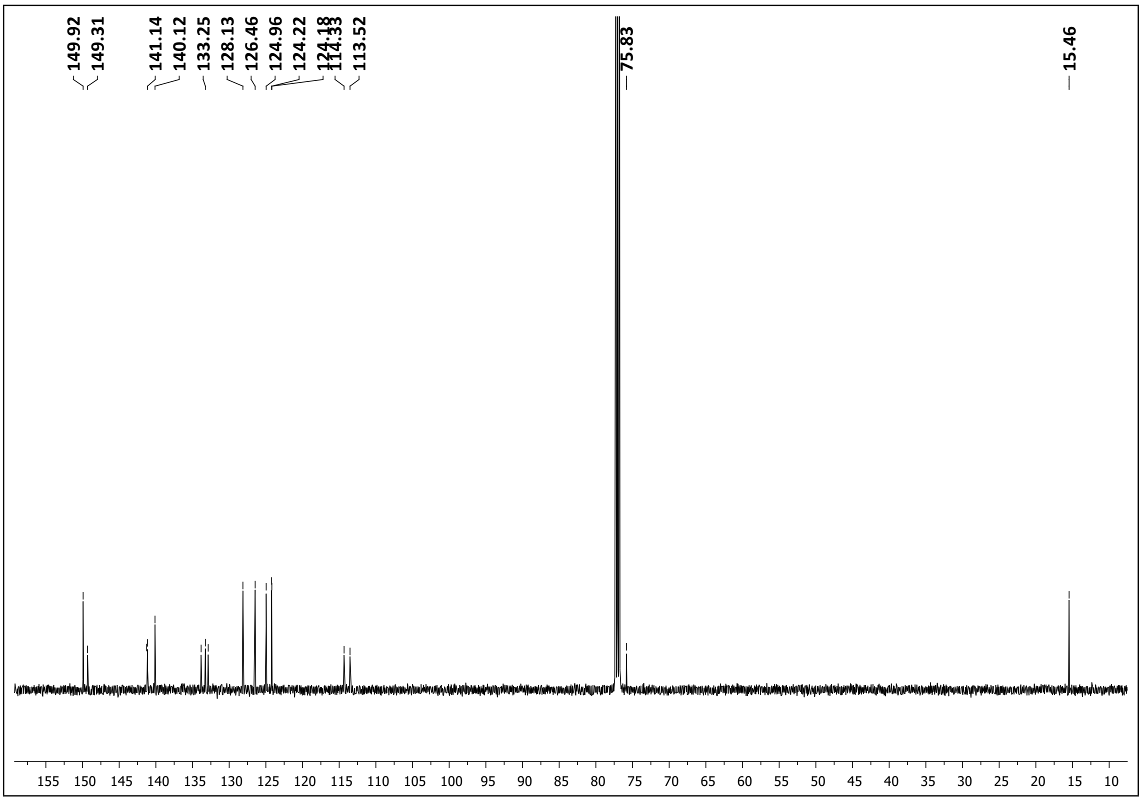

^{13}C NMR spectrum of compound **30**

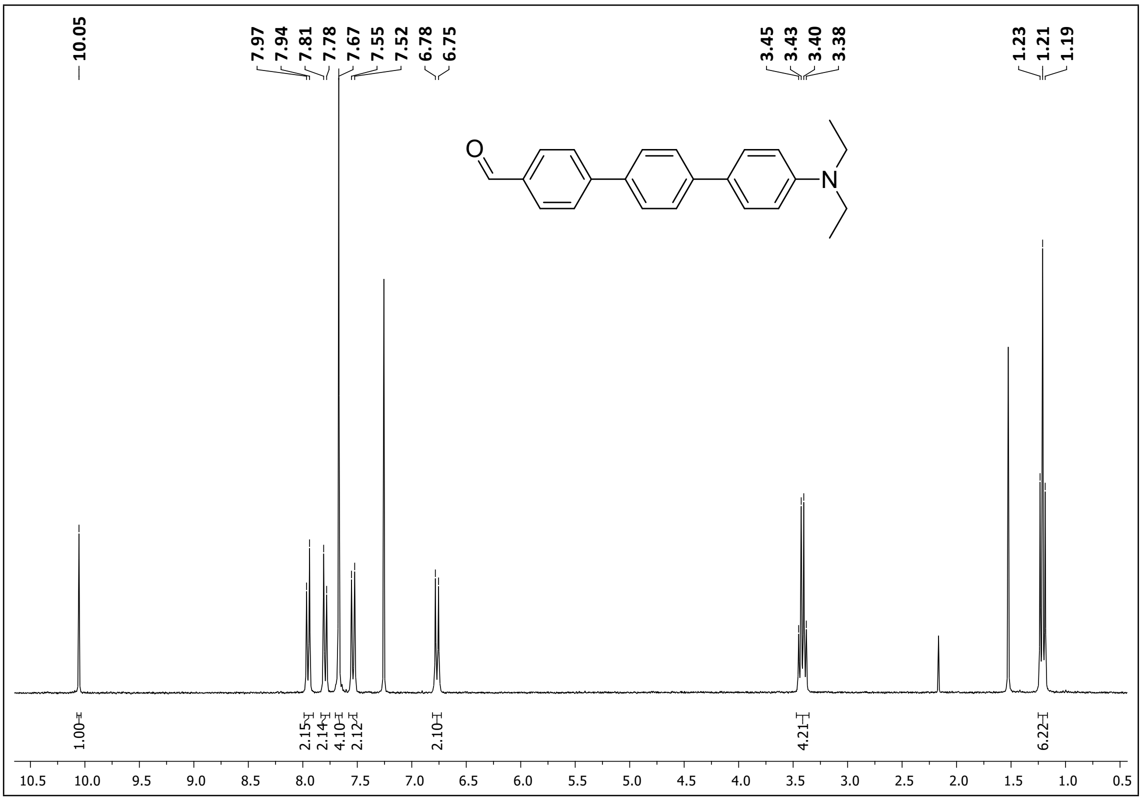

^{1}H NMR spectrum of compound **33**

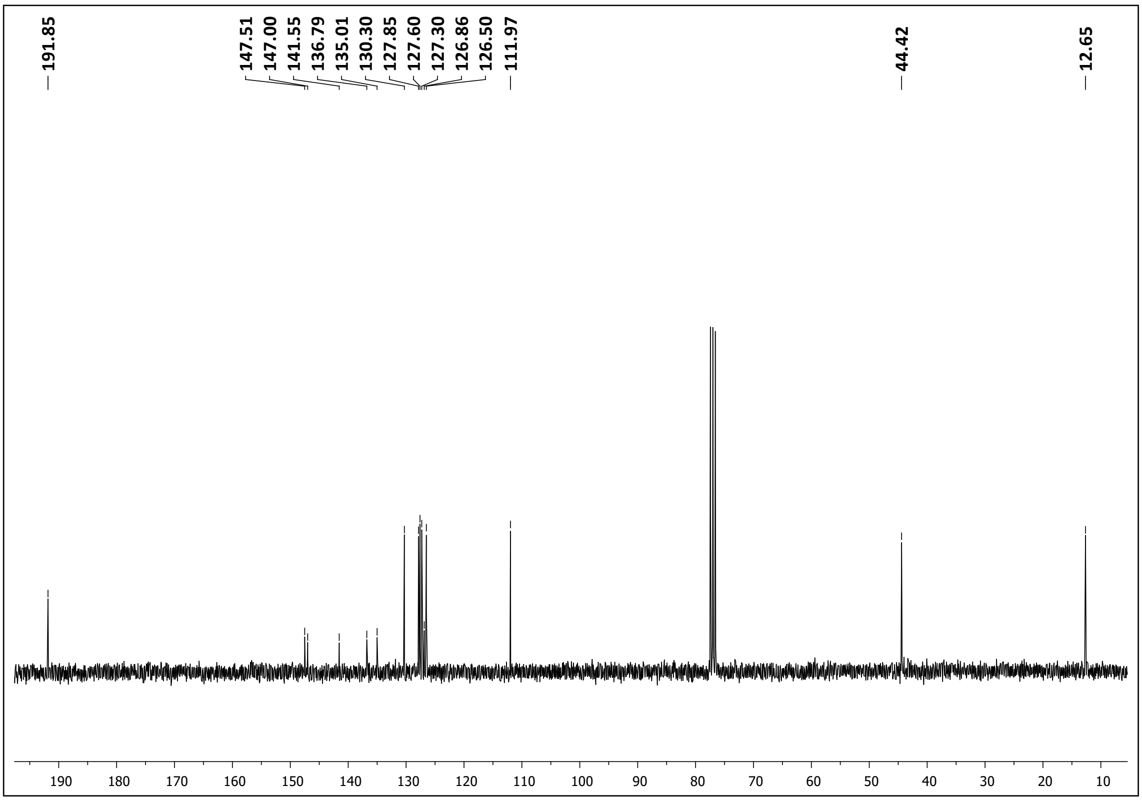

^{13}C NMR spectrum of compound **33**

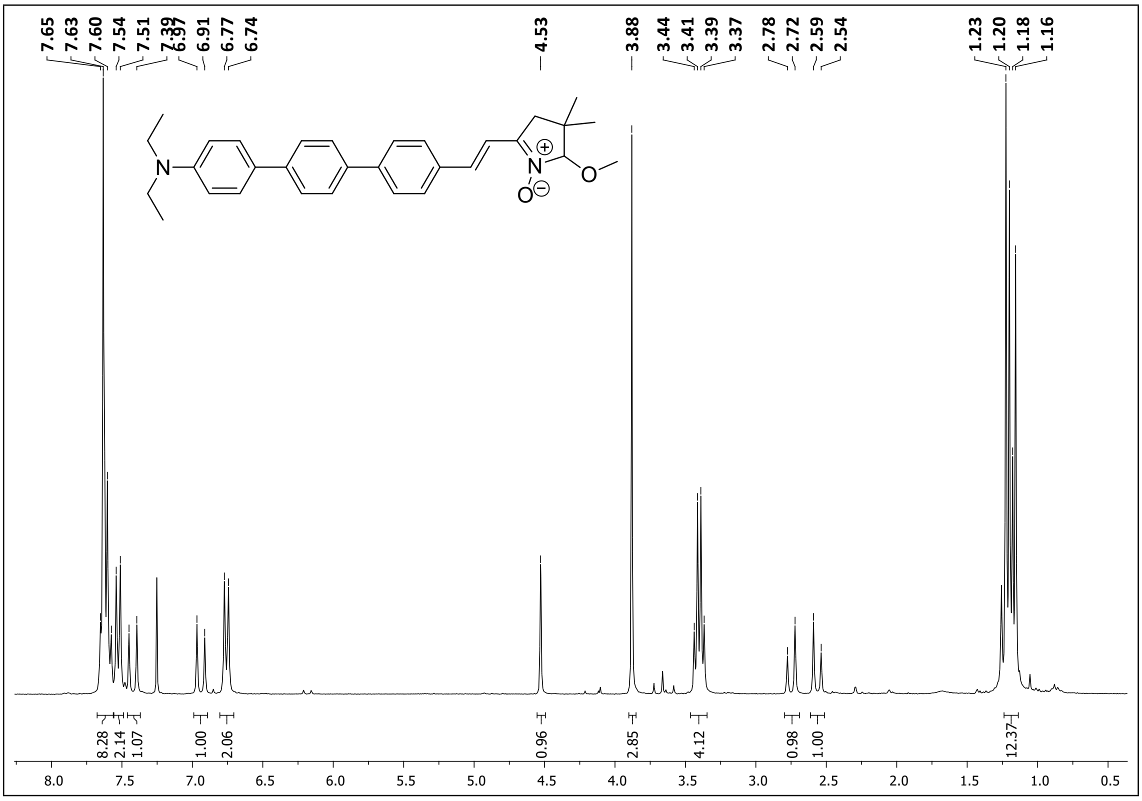

^{1}H NMR spectrum of compound **34**

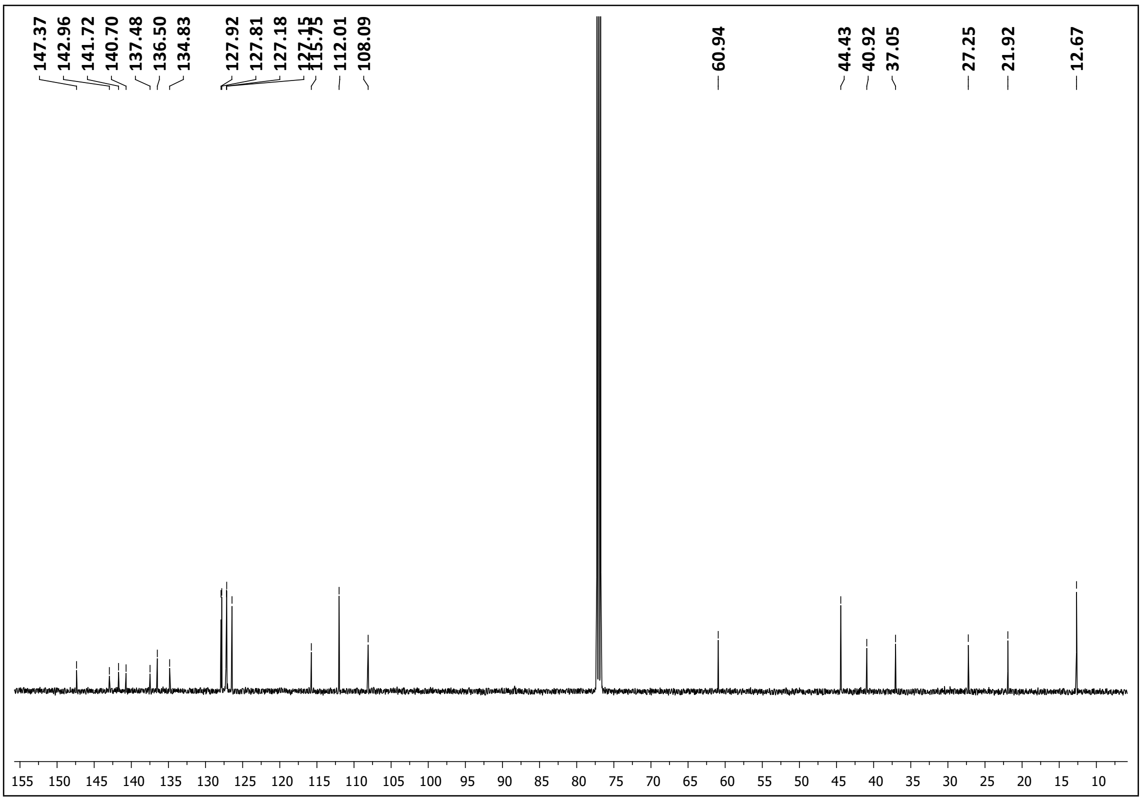

^{13}C NMR spectrum of compound **34**

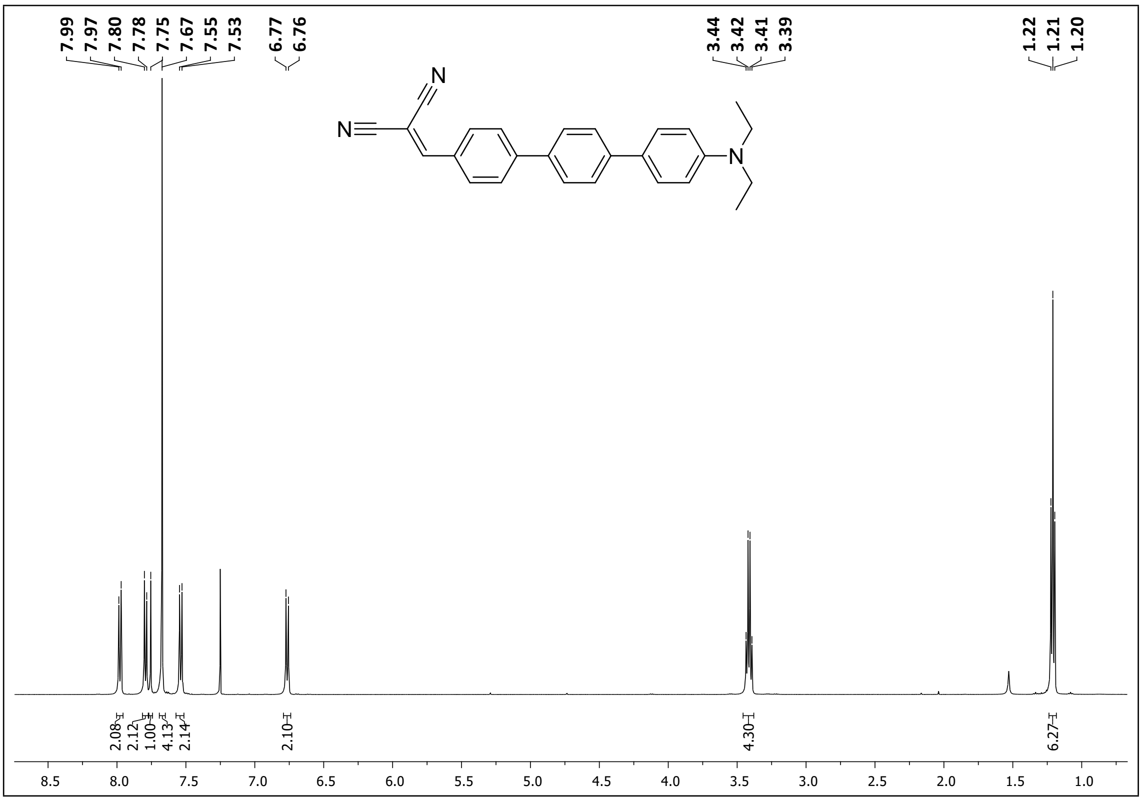

^{1}H NMR spectrum of compound **35**

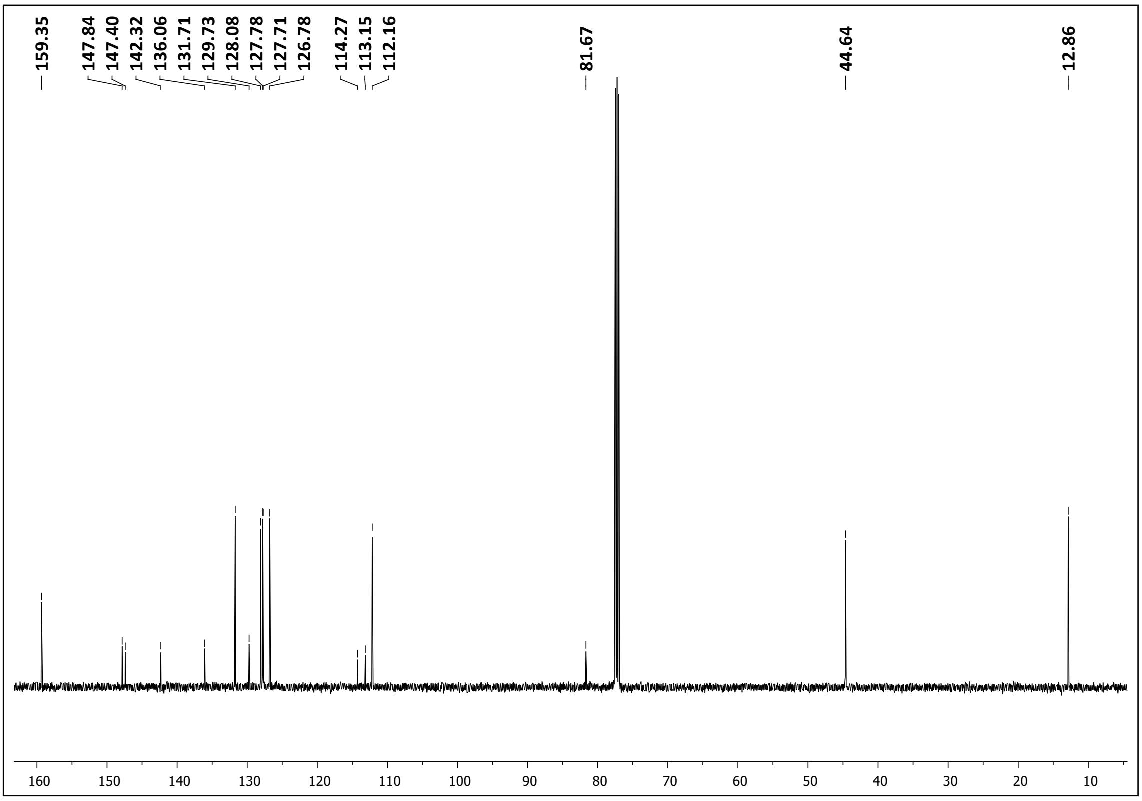

^{13}C NMR spectrum of compound **35**
