# Supplementary material for: Synthesis, optical, electrochemical, and computational study of benzene/thiophene based D–π–A chromophores
Source: RSC Adv. 2024 Nov 5;14(48):35424–37. doi: 10.1039/d4ra02668c (PMC11537213; doi:10.1039/d4ra02668c)
Supplement: RA-014-D4RA02668C-s002 [file RA-014-D4RA02668C-s002.pdf]

This journal is © The Royal Society of Chemistry 2024

## IV. ELECTROCHEMISTRY

### Apparatus

Swiss µAUTOLAB, TYPE III (Metrohm, Herisau, Switzerland) operated the three-electrode electrochemical cell under the software GPES to collect the cyclic voltammetry (CV) data. A platinum wire with the area of 7.1 $mm^2$ served as the working electrode. A Pt-plate and Ag/AgCl/3M KCl-electrode were chosen as the counter and the reference electrode, respectively. The redox signals of 1 mM solutions of all synthetized molecular complexes were measured in dichloromethane (DCM) with 0.1 M tetrabutylammonium hexafluorophosphate ($TBAPF_6$) as the supporting electrolyte against the Ag/AgCl/3M KCl aqueous reference electrode separated from the main electrolytic compartment by a fritted junction containing the same supporting electrolyte (0.1M $TBAPF_6$ in DCM). The potential ranges from 0 V to -2V and from 0 V to + 2 V for the reduction and oxidation were used, respectively. As an internal redox standard $Fc^+/Fc$ (ferrocenium/ferrocene) couple and for the evaluation of HOMO, LUMO, gap energy the scan rate of 75 mV/s was chosen. All CV experiments were performed under argon atmosphere at constant room temperature (23 °C) and with repeatability of RSD ≤ 3.2 %.

$$E_{HOMO} = -(E_{onset,\,ox} - E^{o}_{Fc+/Fc} + 4.89)\ (eV) \quad (1)$$

$$E_{LUMO} = -(E_{onset,\,red} - E^{o}_{Fc+/Fc} + 4.89)\ (eV) \quad (2)$$

where $E^{o}_{Fc+/Fc}$ is the formal redox potential of the $Fc^+/Fc$, which is calculated from its anodic ($E_{pa}$) and cathodic ($E_{pc}$) potentials, i.e., $E^{o}_{Fc+/Fc} = (E_{pa} + E_{pc})/2$.

VOLTAMMETRIC RECORDS

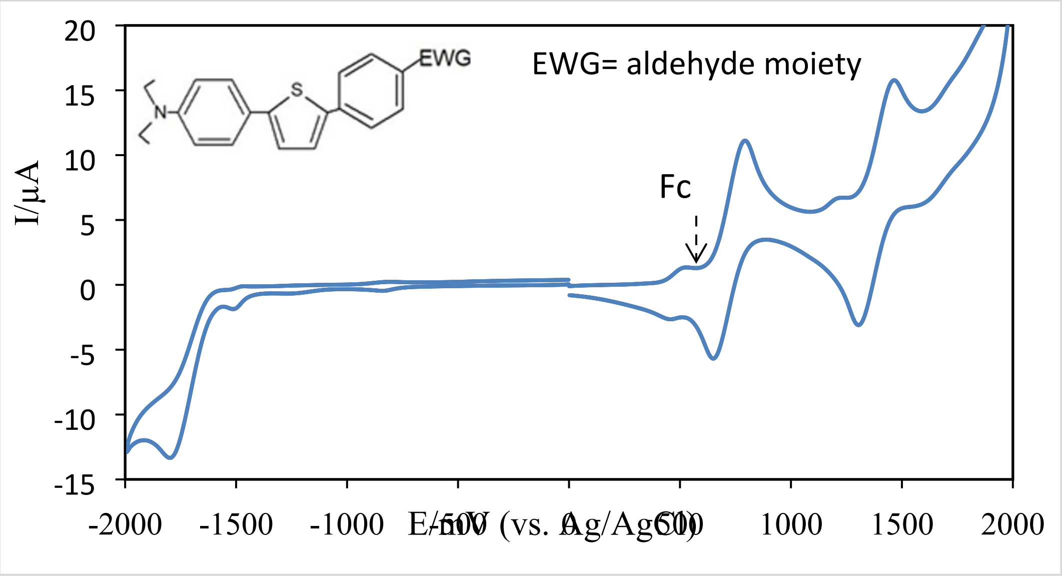

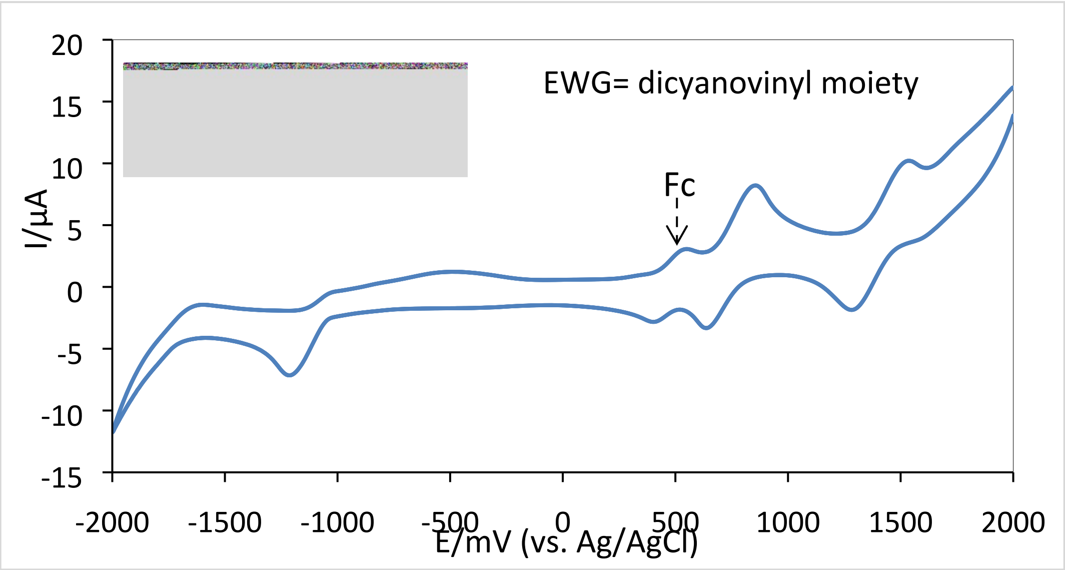
EWG= dicyanovinyl moiety
Fc
I/μA
20
15
10
5
0
-5
-10
-15
-2000
-1500
-1000
-500
0
500
1000
1500
2000
E/mV (vs. Ag/AgCl)

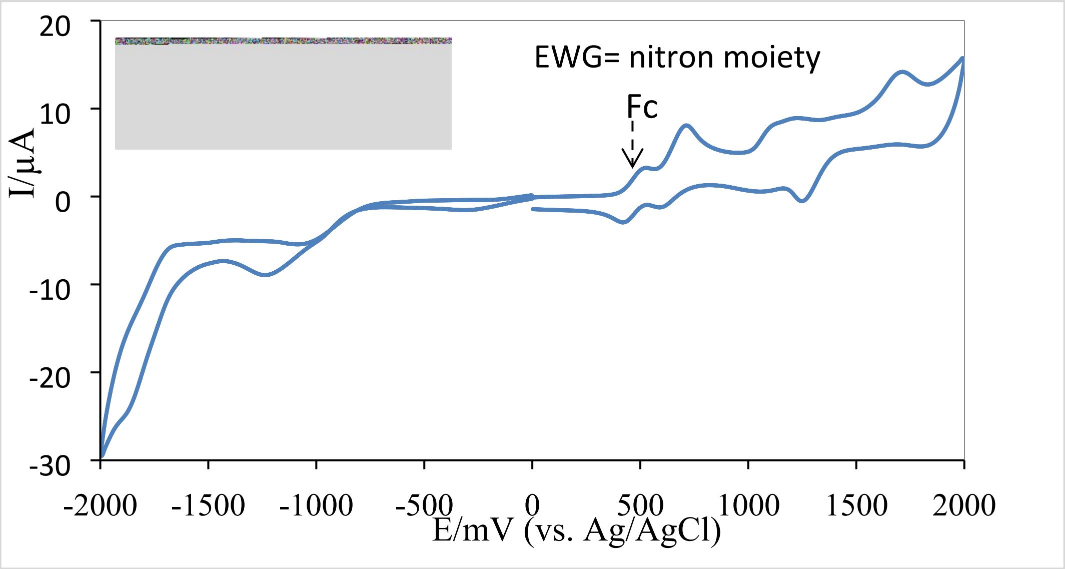
EWG= nitron moiety
Fc
I/μA
20
10
0
-10
-20
-30
-2000
-1500
-1000
-500
0
500
1000
1500
2000
E/mV (vs. Ag/AgCl)

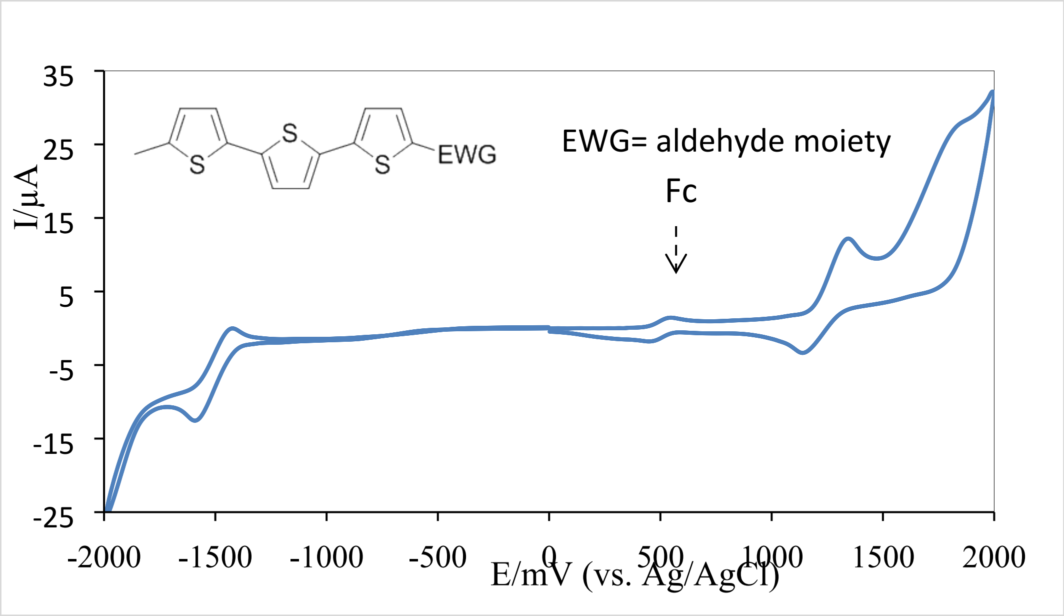
S
S
S
EWG
EWG= aldehyde moiety
Fc
I/μA
35
25
15
5
-5
-15
-25
-2000
-1500
-1000
-500
0
500
1000
1500
2000
E/mV (vs. Ag/AgCl)

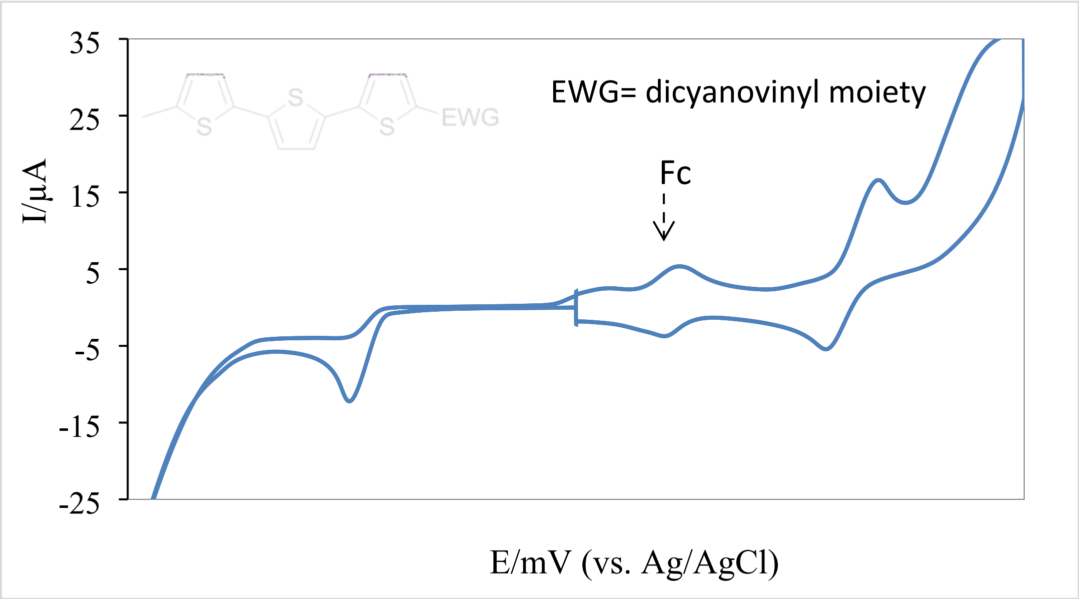
35
25
15
5
-5
-15
-25
I/μA
S
S
S
EWG
EWG= dicyanovinyl moiety
Fc
E/mV (vs. Ag/AgCl)

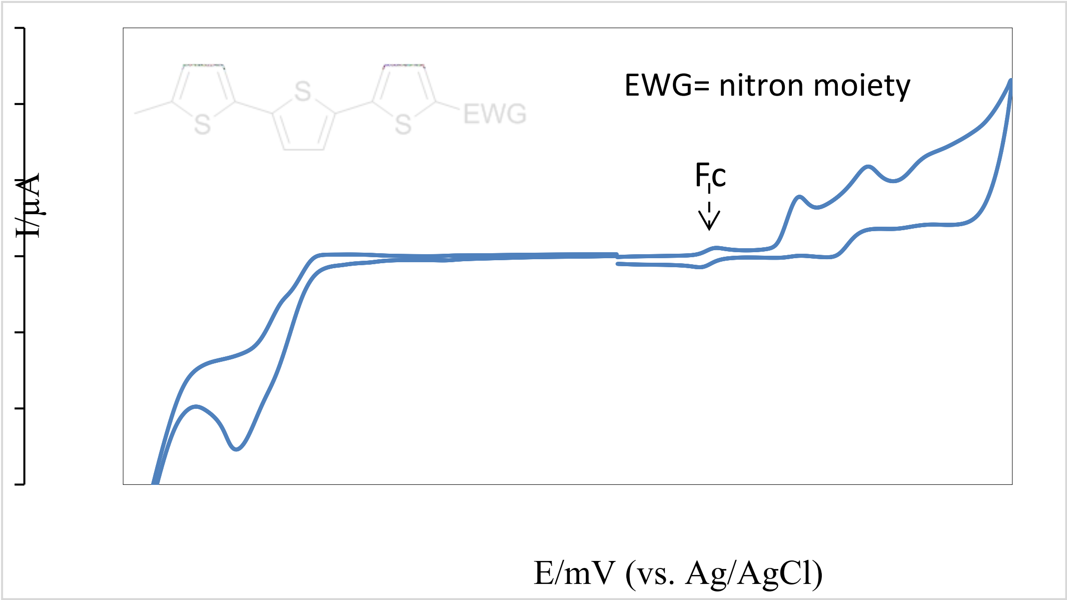
I/μA
S
S
S
EWG
EWG= nitron moiety
Fc
E/mV (vs. Ag/AgCl)

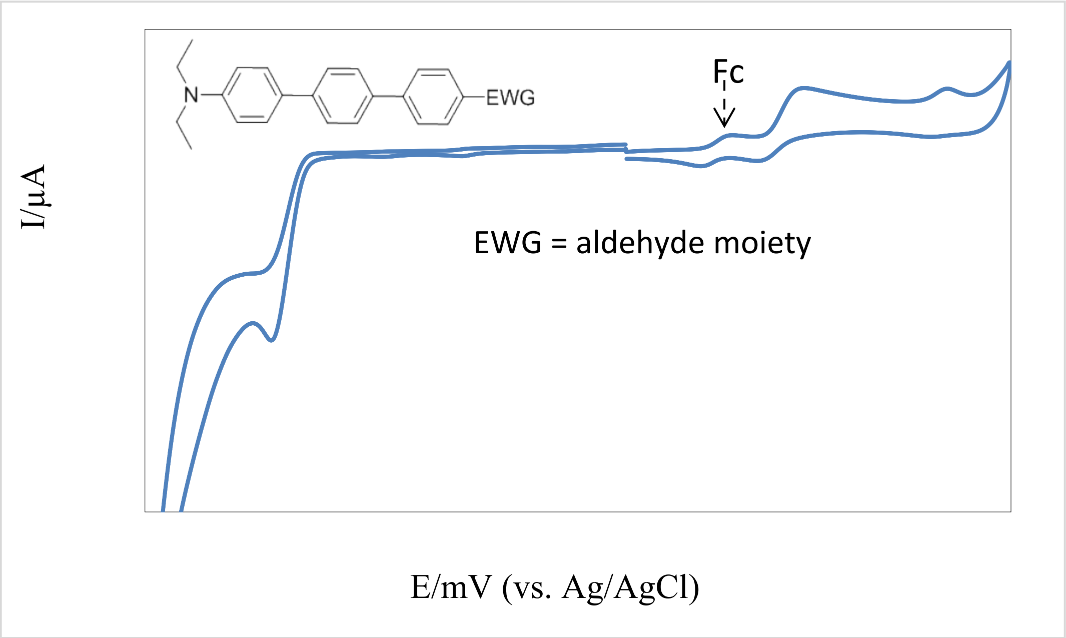
I/μA
N
EWG
Fc
EWG = aldehyde moiety
E/mV (vs. Ag/AgCl)

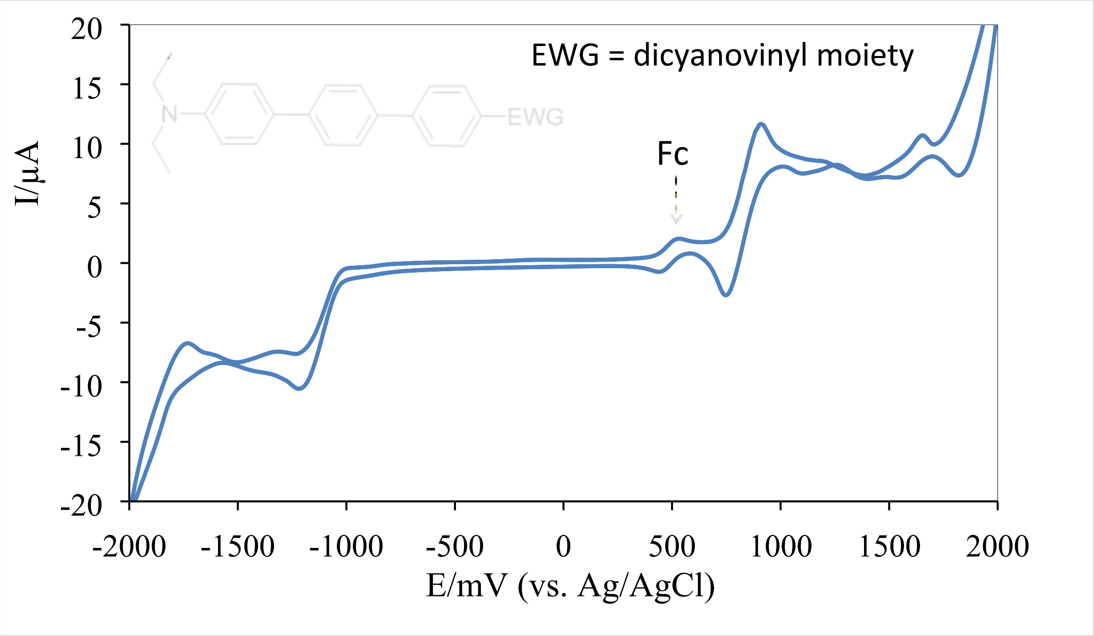
EWG = dicyanovinyl moiety
N
EWG
Fc
I/μA
E/mV (vs. Ag/AgCl)
20
15
10
5
0
-5
-10
-15
-20
-2000
-1500
-1000
-500
0
500
1000
1500
2000

## V. UV-VIS SPECTRA

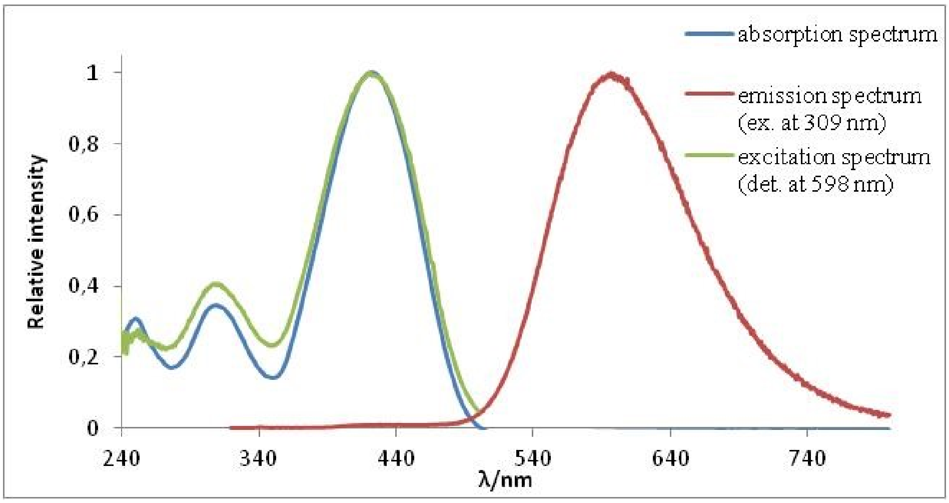


Compound 20
Compound 21

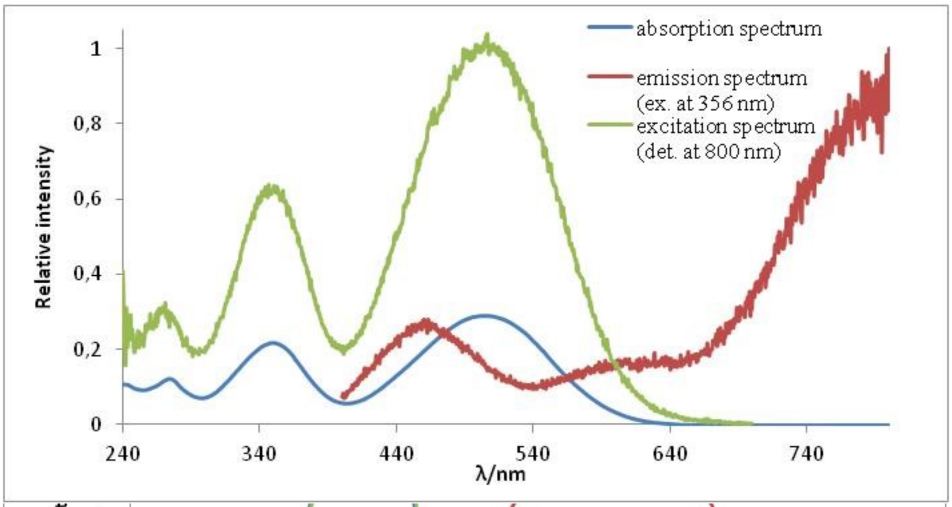


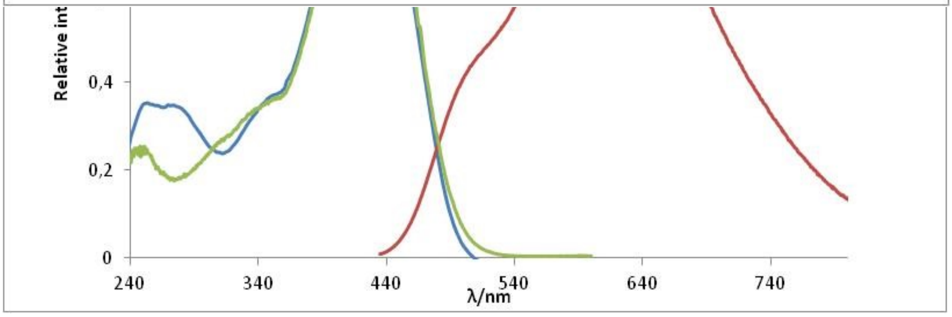

Compound 23

Compound 28

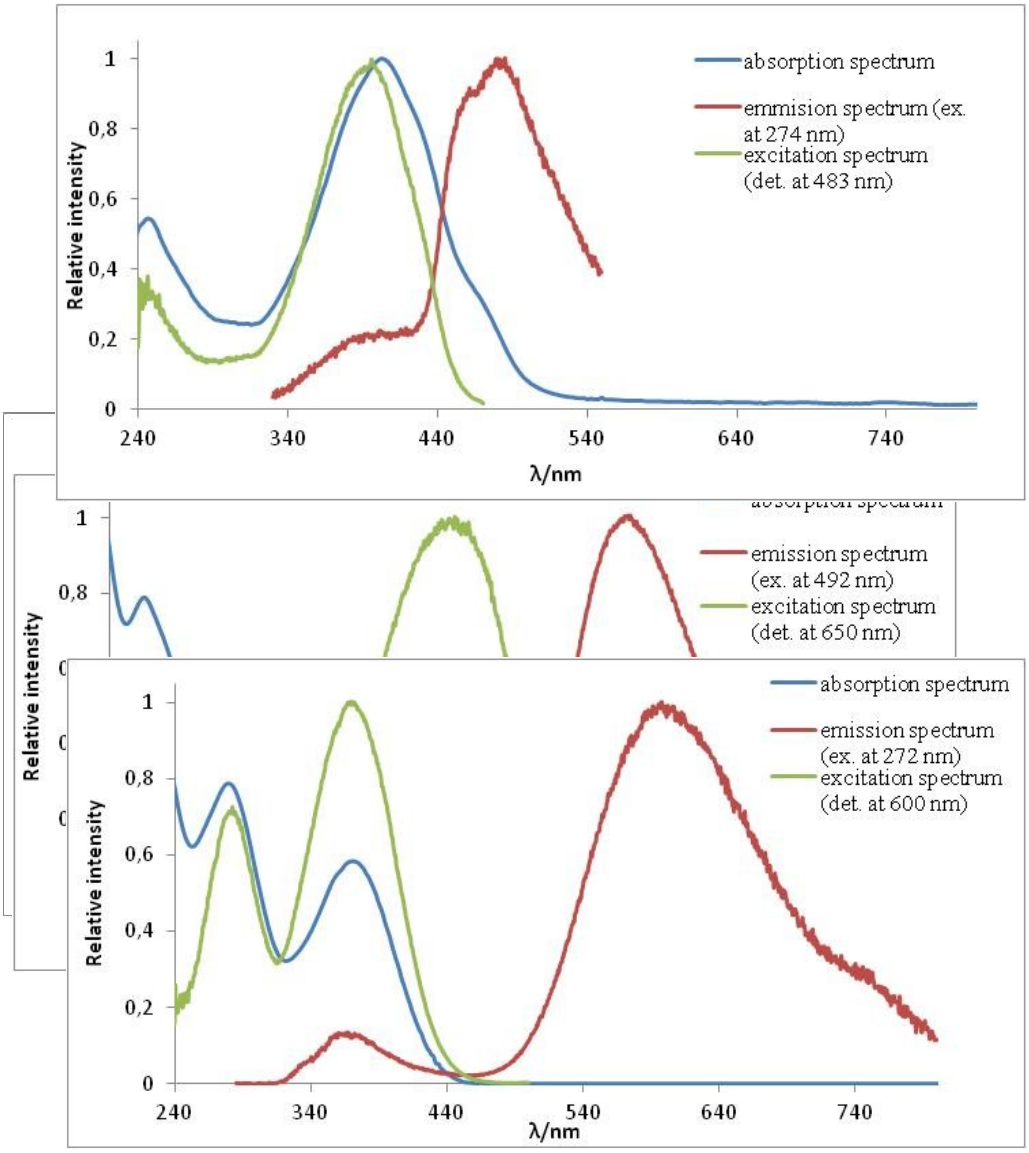


Compound 30

Compound 29

Compound 33
Compound 35

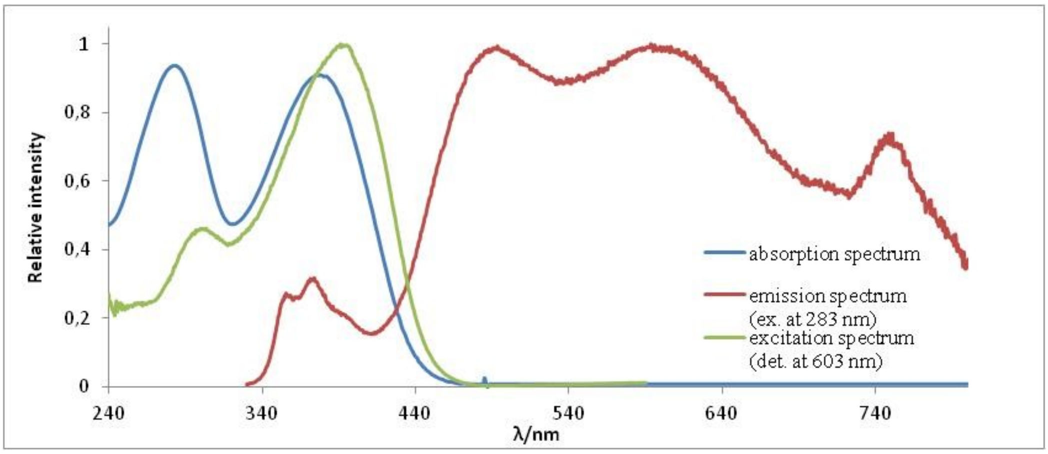


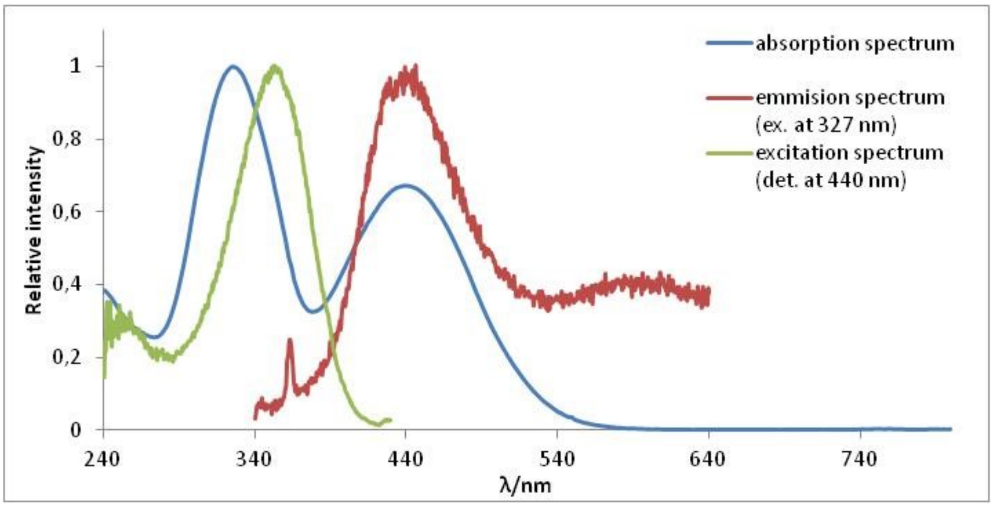


Compound 34

## VI. THEORETICAL CALCULATIONS

| Structure | Torsion angles |
|---|---|
| **20** | −18.2°/14.4° |
| **23** | 13.6°/−8.0° |

Table 5: Torsion angles of synthesised molecules

| Structure | Torsion angles |
|---|---|
| **20** | −18.2°/14.4° |

| | |
|---|---|
| **23** | 13.6°/−8.0° |
| **21** | 8.7°/12.7° |
| **28** | −0.5°/0.1° |
| **30** | −8.0°/0.0° |
| **29** | −10.5°/7.9° |
| **33** | 29.9°/−32.5° |
| **35** | 28.6°/−29.2° |
| **34** | 30.7°/−32.6° |
| **39** | 34.7°/−34.8° |

## Follows frontier orbitals of D-π-A derivatives

| | HOMO | LUMO |
|---|---|---|
| **20** | 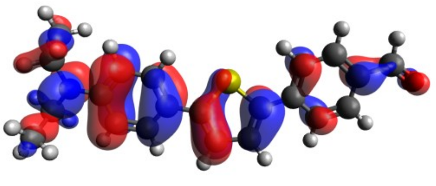 | 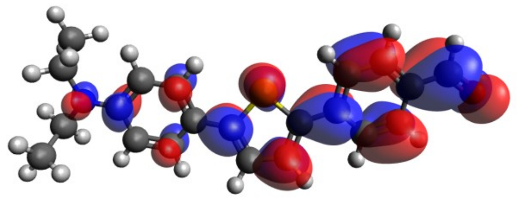 |
| **23** | 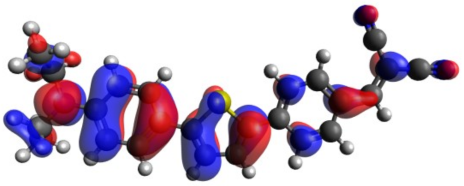 | 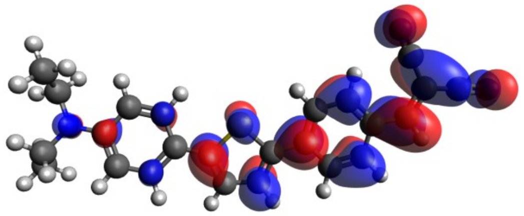 |
| **21** | 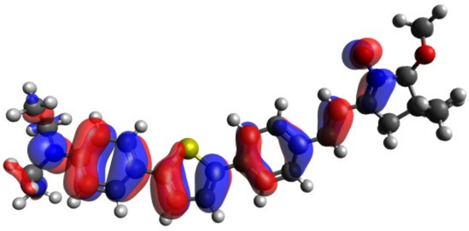 | 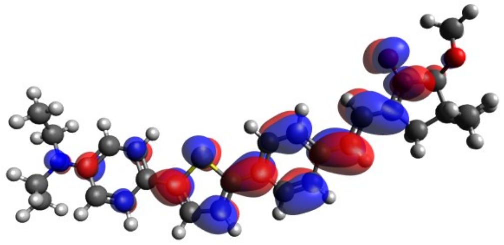 |
| **28** | 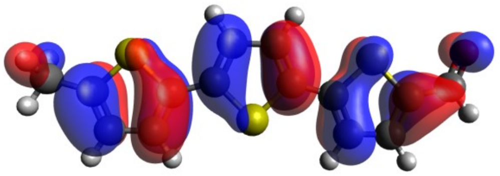 | 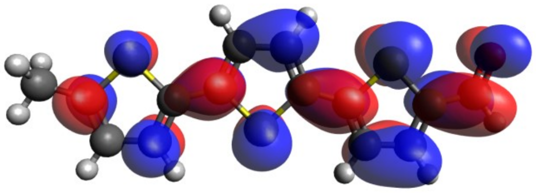 |
| **30** | 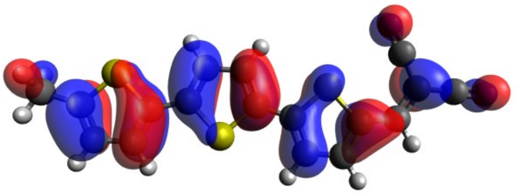 | 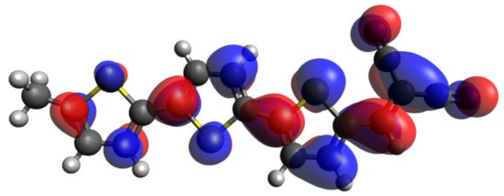 |
| **29** | 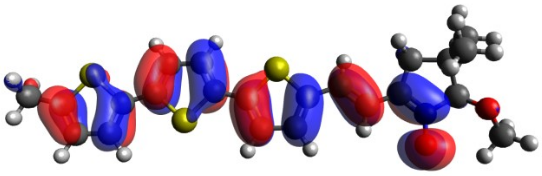 | 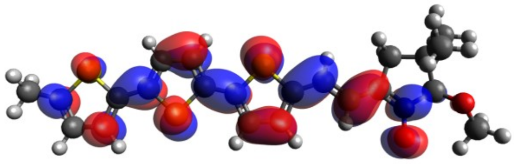 |
| **33** | 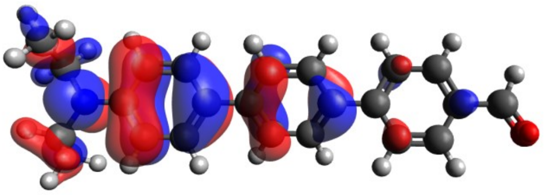 | 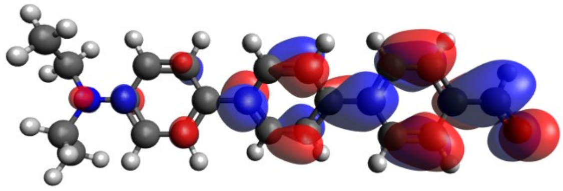 |
| **35** | 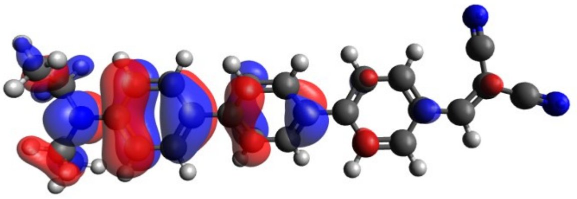 | 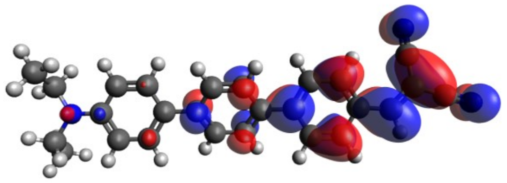 |

| | | |
|---|---|---|
| **34** | 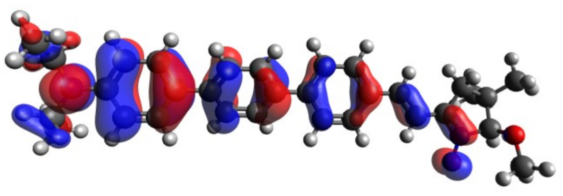 | 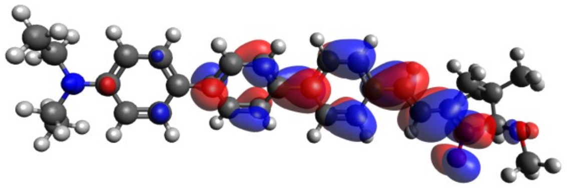 |
| **39** | 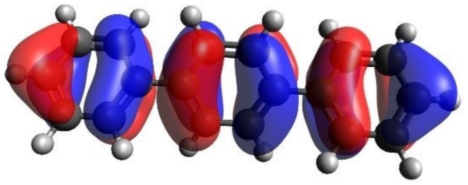 | 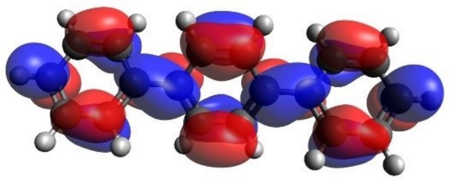 |

Table 6: Cartesian coordinates of related structures

| **20** | *x* | *y* | *z* |
|---|---|---|---|
| C | −3.5818463631 | 0.5562174440 | −0.0137209529 |
| C | −2.9618374851 | −0.6795348685 | −0.0342920576 |
| S | −2.3716748949 | 1.8296858724 | −0.0087526488 |
| C | −1.0443842336 | 0.6727236541 | −0.0134873739 |
| C | −1.5482090698 | −0.6128039610 | −0.0359334093 |
| H | −3.5129386321 | −1.6128385882 | −0.0195586057 |
| H | −0.9160612794 | −1.4937268101 | −0.0217259234 |
| C | −5.0055349239 | 0.8639227985 | −0.0131251757 |
| C | 0.3457364368 | 1.1050803460 | 0.0066494816 |
| C | 0.7042574985 | 2.4315417655 | 0.3247866863 |
| C | 1.3808348856 | 0.1890922254 | −0.2988706484 |
| C | 2.0351179999 | 2.8251685015 | 0.3379668808 |
| C | 2.7068331586 | 0.5821605630 | −0.2766237978 |
| C | 3.0530568723 | 1.9076021204 | 0.0413258643 |
| C | −5.5073715779 | 2.1182724619 | 0.3811304475 |
| C | −5.9538009757 | −0.0986125982 | −0.4123408546 |
| C | −7.3133281336 | 0.1660766353 | −0.4116360551 |
| C | −7.8200412402 | 1.4321006740 | −0.0162243405 |
| C | −6.8635731216 | 2.4029565599 | 0.3787537486 |

| | | | |
|---|---|---|---|
| H | −4.8222438990 | 2.8952603723 | 0.7108338271 |
| H | −7.1805048131 | 3.3931632446 | 0.6808158849 |
| H | −5.6181083647 | −1.0776193034 | −0.7412014973 |
| H | −7.9893797990 | −0.6225316668 | −0.7174845474 |
| H | 1.1300373169 | −0.8311910185 | −0.5685857600 |
| H | −0.0649643183 | 3.1548686569 | 0.5781728846 |
| H | 2.2947341639 | 3.8512968781 | 0.5872554811 |
| H | 3.4968087764 | −0.1240717561 | −0.5128674868 |
| C | 4.4559655121 | 2.3388868263 | 0.0640163978 |
| O | 5.4144446205 | 1.6184544496 | −0.1789111813 |
| H | 4.6066942472 | 3.4070363183 | 0.3266897118 |
| N | −9.1673048750 | 1.7038422934 | −0.0168128161 |
| C | −9.6978193142 | 2.9633995368 | 0.4998261394 |
| C | −9.7213659007 | 4.0830686900 | −0.5426439374 |
| H | −9.1227488950 | 3.2668190111 | 1.3801588988 |
| H | −10.7145617751 | 2.7657161142 | 0.8534874292 |
| H | −8.7129967694 | 4.3084698803 | −0.9051804594 |
| H | −10.1444425496 | 4.9981101126 | −0.1113744808 |
| H | −10.3348444780 | 3.7961150302 | −1.4044091905 |
| C | −10.1465876752 | 0.7475995884 | −0.5282657043 |
| C | −10.6084819594 | −0.2648289564 | 0.5217944279 |
| H | −9.7332894274 | 0.2359984106 | −1.4026710233 |
| H | −11.0039488933 | 1.3241596732 | −0.8898977634 |
| H | −9.7687525026 | −0.8627776008 | 0.8912625432 |
| H | −11.3541732396 | −0.9458392980 | 0.0946136267 |
| H | −11.0633600947 | 0.2461384238 | 1.3781103321 |

| **23** | *x* | *y* | *z* |
|---|---|---|---|
| S | −0.0900996689 | −0.0006664822 | −0.1180806330 |
| C | 1.2499882378 | −0.9965857730 | 0.4242067915 |
| C | −1.2848022824 | −1.1656769097 | 0.4487491482 |
| C | 0.7668683858 | −2.1641015312 | 0.9954547414 |
| C | −0.6401675345 | −2.2567113475 | 1.0070633230 |

| | | | |
|---|---|---|---|
| H | 1.4140432849 | −2.9236934024 | 1.4178891332 |
| H | −1.1699188630 | −3.0983510017 | 1.4384870756 |
| C | 2.6293285947 | −0.5756626849 | 0.2443026244 |
| C | 3.6919982503 | −1.4886781897 | 0.4051196865 |
| C | 5.0136735001 | −1.1113600973 | 0.2462255957 |
| C | 5.3670472234 | 0.2210921363 | −0.0968006420 |
| C | 4.2964523664 | 1.1385892757 | −0.2646006779 |
| C | 2.9797823975 | 0.7460648634 | −0.0943433098 |
| H | 2.2039652441 | 1.4966846302 | −0.2222839164 |
| H | 3.4789644288 | −2.5236494667 | 0.6538470881 |
| H | 5.7820906976 | −1.8585346082 | 0.3980475655 |
| H | 4.4943546462 | 2.1667428081 | −0.5395351007 |
| C | −2.7063546184 | −0.9278659900 | 0.3136813258 |
| C | −3.2151060574 | 0.3154693732 | −0.1331071675 |
| C | −3.6412142619 | −1.9421388868 | 0.6277773211 |
| C | −4.9974718826 | −1.7156081888 | 0.5090264069 |
| C | −4.5716547308 | 0.5429983115 | −0.2539562127 |
| C | −5.5074807472 | −0.4689041931 | 0.0687125381 |
| H | −3.2937582480 | −2.9144741670 | 0.9581876958 |
| H | −2.5314035056 | 1.1220427404 | −0.3796752165 |
| H | −4.9047678251 | 1.5136697158 | −0.5975779875 |
| H | −5.6952340378 | −2.5110293523 | 0.7552937404 |
| C | −6.9344495294 | −0.3417128405 | −0.0116114497 |
| N | 6.6757633678 | 0.6006391348 | −0.2580718694 |
| C | 7.0517090074 | 1.9932672319 | −0.4960734951 |
| C | 7.7717110625 | −0.3649187089 | −0.1964455147 |
| C | −7.7332772922 | 0.7201701906 | −0.3694627150 |
| H | −7.4836406430 | −1.2397319897 | 0.2608978572 |
| C | −9.1524167918 | 0.5575139281 | −0.3605503479 |
| C | −7.2752444141 | 2.0132816929 | −0.7575109770 |
| N | −10.3095169455 | 0.4269224860 | −0.3543799787 |
| N | −6.9284274927 | 3.0781271718 | −1.0767929614 |
| C | 8.2776761771 | −0.6138404392 | 1.2257246002 |
| H | 8.5838121772 | 0.0288224671 | −0.8154616176 |
| H | 7.4578284901 | −1.3024358323 | −0.6652390722 |
| H | 8.6347820497 | 0.3182686127 | 1.6782247401 |

| | | | |
|---|---|---|---|
| H | 9.1087520520 | −1.3288969840 | 1.2152067216 |
| H | 7.4845145263 | −1.0176439249 | 1.8636255152 |
| C | 7.0317020871 | 2.3791000025 | −1.9763375301 |
| H | 8.0591631912 | 2.1298324491 | −0.0905639616 |
| H | 6.3973145013 | 2.6504014630 | 0.0844713216 |
| H | 7.7192182180 | 1.7472609870 | −2.5502100243 |
| H | 7.3409572694 | 3.4233591068 | −2.1026331481 |
| H | 6.0295898413 | 2.2633275125 | −2.4023454291 |

| **21** | *x* | *y* | *z* |
|---|---|---|---|
| C | −2.7602779558 | 0.7281587450 | 0.3680325067 |
| C | −2.3296189331 | −0.5528203803 | 0.6563487998 |
| S | −1.3703185946 | 1.7921009924 | 0.1910418396 |
| C | −0.2300585435 | 0.4869420375 | 0.5054118165 |
| C | −0.9209634554 | −0.6855654196 | 0.7331506905 |
| C | 1.2094550416 | 0.7154234975 | 0.4941574248 |
| C | 1.7585739544 | 2.0158840276 | 0.5035767506 |
| C | 3.1281877749 | 2.2240431693 | 0.4902329747 |
| C | 2.1105867027 | −0.3687070018 | 0.4716109985 |
| C | 3.4811008230 | −0.1578959409 | 0.4656246835 |
| C | 4.0311042131 | 1.1404392898 | 0.4724872509 |
| C | −4.1212315267 | 1.2275826208 | 0.2124847944 |
| C | −4.4013098780 | 2.5222590694 | −0.2628782097 |
| C | −5.2332163177 | 0.4227258277 | 0.5307920613 |
| C | −6.5350192699 | 0.8708749117 | 0.3757439648 |
| C | −6.8161948278 | 2.1757811443 | −0.1061981678 |
| C | −5.6971509293 | 2.9900675492 | −0.4155335241 |
| H | −3.0092106406 | −1.3846265991 | 0.8030075431 |
| H | −0.4293237784 | −1.6248620342 | 0.9612887642 |
| H | 1.7310861353 | −1.3848716928 | 0.4442011315 |
| H | 1.1005254532 | 2.8799052690 | 0.5336333514 |
| H | 3.5037168356 | 3.2423995467 | 0.4996142473 |
| H | 4.1515918572 | −1.0135520410 | 0.4459303186 |
| H | −3.5859919638 | 3.1914661134 | −0.5259373970 |
| H | −5.8382664038 | 4.0032119704 | −0.7700129949 |

| | | | |
|---|---|---|---|
| H | −5.0790019399 | −0.5825441635 | 0.9107863299 |
| H | −7.3430975266 | 0.1938536582 | 0.6229147594 |
| N | −8.1057074488 | 2.6276719525 | −0.2641435788 |
| C | −8.3959960733 | 3.9238966255 | −0.8722684364 |
| C | −8.3907499163 | 5.0781010633 | 0.1322189160 |
| H | −9.3808016843 | 3.8480715642 | −1.3439802755 |
| H | −7.6844688970 | 4.1139361998 | −1.6818376658 |
| H | −9.1346797243 | 4.9093614273 | 0.9192785551 |
| H | −8.6320137650 | 6.0228767522 | −0.3693851598 |
| H | −7.4107876526 | 5.1830664900 | 0.6095448855 |
| C | −9.2572496014 | 1.8286488280 | 0.1475712152 |
| C | −9.7261457997 | 0.8451225293 | −0.9269057185 |
| H | −10.0659670513 | 2.5253194494 | 0.3902635788 |
| H | −9.0210985571 | 1.3009441135 | 1.0767345932 |
| H | −10.0064924015 | 1.3777765800 | −1.8428784243 |
| H | −10.6005419200 | 0.2836491793 | −0.5768070095 |
| H | −8.9366738899 | 0.1295748519 | −1.1796832462 |
| C | 5.4769884452 | 1.2967382342 | 0.4568585762 |
| C | 6.1654141787 | 2.4664554119 | 0.4553652350 |
| H | 6.0396206070 | 0.3646329982 | 0.4425774354 |
| C | 7.5913172509 | 2.5434929822 | 0.4352343693 |
| H | 5.6530887245 | 3.4243819164 | 0.4671815938 |
| C | 8.5842454675 | 1.4171617315 | 0.3906886187 |
| C | 9.9309712466 | 2.0841505916 | 0.7807490268 |
| H | 8.6327112205 | 0.9881867571 | −0.6197271864 |
| H | 8.3181271179 | 0.6044435061 | 1.0744845211 |
| C | 9.7176164685 | 3.5329707888 | 0.3024861914 |
| C | 11.1322746838 | 1.4430430162 | 0.0850279795 |
| C | 10.1090211735 | 2.0401613497 | 2.3059392109 |
| N | 8.2174268119 | 3.7097961626 | 0.4124739109 |
| O | 7.7067463579 | 4.8802634173 | 0.4141496553 |
| O | 10.4009601429 | 4.4646416052 | 1.0625487492 |
| H | 9.9500138472 | 3.6480103049 | −0.7662254091 |
| C | 10.6814196183 | 5.6980055505 | 0.3952095304 |
| H | 9.7592999689 | 6.2224477341 | 0.1345977438 |
| H | 11.2772638413 | 5.5218072147 | −0.5113949215 |

| | | | |
|---|---|---|---|
| H | 11.2659102384 | 6.2986857653 | 1.0966147119 |
| H | 11.0153505983 | 1.4559794833 | −1.0047478023 |
| H | 11.2491759088 | 0.4004081091 | 0.4021224030 |
| H | 12.0557040997 | 1.9777328063 | 0.3364361055 |
| H | 9.2525179105 | 2.4835528682 | 2.8252675846 |
| H | 11.0074167503 | 2.5826959803 | 2.6117757179 |
| H | 10.2043654654 | 0.9987291259 | 2.6325835954 |

| **28** | *x* | *y* | *z* |
|---|---|---|---|
| S | −1.2851342512 | 1.2716641512 | −0.0519889138 |
| C | −2.5871483186 | 0.0947106672 | 0.0142319666 |
| C | −2.0737267955 | −1.1878611454 | 0.0809063093 |
| C | −0.6615817909 | −1.2353533870 | 0.0806225475 |
| C | −0.0616295589 | 0.0077846934 | 0.0134687437 |
| H | −2.6975533854 | −2.0736867950 | 0.1288775022 |
| H | −0.1006368222 | −2.1625710184 | 0.1291848663 |
| C | −3.9643723212 | 0.5206530366 | −0.0044891923 |
| C | 1.3391240956 | 0.3375138262 | −0.0060460728 |
| S | −5.2734747544 | −0.6567144137 | 0.0484266378 |
| C | −6.4918876232 | 0.5999554378 | −0.0069285517 |
| C | −5.9023656390 | 1.8370352019 | −0.0623214956 |
| C | −4.4803925278 | 1.7980017205 | −0.0610387658 |
| H | −6.4784534014 | 2.7555058093 | −0.1025238089 |
| H | −3.8599682298 | 2.6875941539 | −0.0992628369 |
| S | 2.5585841584 | −0.9213486435 | 0.0562437298 |
| C | 3.8418060584 | 0.2695470084 | −0.0065301899 |
| C | 1.9252822114 | 1.5958713836 | −0.0713638535 |
| C | 3.3340495307 | 1.5534113422 | −0.0713347678 |
| H | 1.3533589890 | 2.5160736970 | −0.1170797959 |
| H | 3.9696469638 | 2.4315590273 | −0.1166353352 |
| C | −7.9476982037 | 0.2576857965 | 0.0090729115 |
| H | −8.2288962516 | −0.3635589595 | −0.8501881152 |
| H | −8.5408888676 | 1.1763977172 | −0.0270237653 |
| H | −8.2261238393 | −0.2923356713 | 0.9163877288 |
| C | 5.2290533600 | −0.1353342960 | 0.0124693170 |

| | | | |
|---|---|---|---|
| O | 5.6161149013 | −1.2980232458 | 0.0719523774 |
| H | 5.9465386006 | 0.7083252573 | −0.0318899109 |

| **30** | *x* | *y* | *z* |
|---|---|---|---|
| S | −1.2951042990 | 1.2871968410 | 0.3248649029 |
| C | −2.5042472743 | 0.0389067509 | 0.0842895402 |
| C | 0.0167626284 | 0.1211330440 | 0.1802962884 |
| C | −1.8988375752 | −1.1937427331 | −0.1036241736 |
| C | −0.4915571967 | −1.1469609755 | −0.0494111843 |
| H | −2.4573781336 | −2.1087921252 | −0.2661036269 |
| H | 0.1358108413 | −2.0237127546 | −0.1705227764 |
| C | −3.9066126713 | 0.3649666397 | 0.0951059679 |
| S | −5.1180530323 | −0.8316918001 | −0.3547256033 |
| C | −6.4258265164 | 0.2996997199 | −0.0866466913 |
| C | −5.9348309253 | 1.5155775531 | 0.3189293312 |
| C | −4.5183732482 | 1.5574709463 | 0.4228900382 |
| H | −3.9692411167 | 2.4380905928 | 0.7397108104 |
| H | −6.5795846164 | 2.3592633504 | 0.5410733977 |
| C | 1.3822098309 | 0.5353845628 | 0.2995290407 |
| S | 2.6866801484 | −0.6249121917 | 0.1535016851 |
| C | 3.8961052334 | 0.6357517835 | 0.4084986095 |
| C | 1.8754770209 | 1.8190152098 | 0.5313275146 |
| C | 3.2706537043 | 1.8711355188 | 0.5910176348 |
| H | 1.2362531152 | 2.6861166276 | 0.6520523519 |
| H | 3.8388022951 | 2.7787724664 | 0.7636154753 |
| C | −7.8487951951 | −0.1063057865 | −0.3021182142 |
| H | −8.1333429348 | −0.9468053375 | 0.3424997884 |
| H | −8.0310978546 | −0.4121111054 | −1.3396631049 |
| H | −8.5085221670 | 0.7362999987 | −0.0745448557 |
| C | 5.2965870664 | 0.4603290193 | 0.4312871370 |
| C | 6.0524897734 | −0.6837395096 | 0.2709050184 |
| H | 5.8558829247 | 1.3769288805 | 0.6032199902 |
| C | 7.4740229002 | −0.6101920111 | 0.3333031209 |
| C | 5.4947636134 | −1.9726860486 | 0.0417922877 |
| N | 5.0348548444 | −3.0270109183 | −0.1444800662 |

| | | | |
|---|---|---|---|
| N | 8.6363158169 | −0.5516830621 | 0.3839081923 |

| **29** | *x* | *y* | *z* |
|---|---|---|---|
| S | −1.4155662834 | 1.7956680225 | −0.2891763813 |
| C | −2.2218538997 | 0.2338669144 | −0.2101902169 |
| C | 0.1751121700 | 1.0459566995 | −0.1995669416 |
| C | −1.2893519628 | −0.7824273705 | −0.1390800197 |
| C | 0.0513272729 | −0.3285949847 | −0.1334977749 |
| H | −1.5616087152 | −1.8304867610 | −0.0751221171 |
| H | 0.9039810951 | −0.9960804035 | −0.0663902207 |
| C | −3.6618458917 | 0.1505288004 | −0.2312792162 |
| C | 1.3669498895 | 1.8518555875 | −0.2069332714 |
| C | −4.5954319645 | 1.1576184934 | −0.1135044661 |
| S | −4.4719580781 | −1.4002354111 | −0.4394286416 |
| C | −6.0572705190 | −0.6576740501 | −0.3539698843 |
| C | −5.9410199174 | 0.6974439927 | −0.1841056737 |
| H | −4.3280110558 | 2.1992320450 | 0.0318870571 |
| H | −6.8039056397 | 1.3507823616 | −0.1062934064 |
| C | −7.2989510239 | −1.4847731900 | −0.4612757729 |
| H | −7.3676355796 | −2.2233936043 | 0.3470657077 |
| H | −7.3433407365 | −2.0329619996 | −1.4104318410 |
| H | −8.1778309685 | −0.8354325961 | −0.4030157589 |
| S | 2.9545358186 | 1.1147461541 | −0.3326246035 |
| C | 3.7600349609 | 2.6783741512 | −0.2707020858 |
| C | 1.4872827441 | 3.2305432890 | −0.1296660001 |
| C | 2.8202450103 | 3.6916168300 | −0.1653610380 |
| H | 0.6315638698 | 3.8916352312 | −0.0395434426 |
| H | 3.0924767378 | 4.7396181591 | −0.1104605494 |
| C | 7.3503948274 | 3.9120242226 | −0.3707531692 |
| C | 5.1957089621 | 2.7373479560 | −0.3329404037 |
| C | 8.3033034355 | 2.7541183754 | −0.4638589043 |
| C | 9.6421963865 | 3.4024612745 | −0.9089095817 |
| C | 9.5164091649 | 4.8218057879 | −0.3234845442 |
| N | 8.0195669245 | 5.0549467505 | −0.3193886549 |
| O | 10.1816313775 | 5.7801434842 | −1.0652191679 |

| | | | |
|---|---|---|---|
| C | 5.9275546858 | 3.8841711708 | −0.3033644075 |
| H | 5.7172952239 | 1.7856703040 | −0.4099100748 |
| H | 5.4429327697 | 4.8535239712 | −0.2254507970 |
| C | 10.8662380921 | 2.6790858681 | −0.3466547307 |
| C | 9.7084291055 | 3.4558695796 | −2.4427694665 |
| H | 7.9618648596 | 1.9943696244 | −1.1745223713 |
| H | 8.4071145627 | 2.2624164863 | 0.5131378667 |
| H | 10.8284062080 | 2.6231078679 | 0.7473736298 |
| H | 11.7877296610 | 3.2003134748 | −0.6310128164 |
| H | 10.9223504028 | 1.6567742245 | −0.7377568236 |
| H | 8.8330715760 | 3.9589672311 | −2.8678186340 |
| H | 9.7435478980 | 2.4364196998 | −2.8424619386 |
| H | 10.6012686920 | 3.9901506492 | −2.7779800500 |
| H | 9.8221848483 | 4.8554636861 | 0.7322490096 |
| C | 10.5532349064 | 6.9542536689 | −0.3378748087 |
| H | 11.1093861291 | 7.5833877951 | −1.0372824426 |
| H | 11.2034547334 | 6.6956387875 | 0.5095599424 |
| H | 9.6714517368 | 7.4887071444 | 0.0229630248 |
| O | 7.5525257040 | 6.2377780507 | −0.2165257709 |

| **33** | *x* | *y* | *z* |
|---|---|---|---|
| C | −2.6401853583 | −0.0357429581 | −0.0992987405 |
| C | −1.9309064972 | 1.0863988242 | −0.5627114379 |
| C | −0.5414245703 | 1.1013632148 | −0.5848955095 |
| H | −0.0316752894 | 1.9708221965 | −0.9885155084 |
| C | 0.2120964614 | 0.0011696680 | −0.1329606997 |
| C | −0.5018471190 | −1.1182291138 | 0.3359101944 |
| C | −1.8913097596 | −1.1390156972 | 0.3468104532 |
| H | −2.4025815215 | −2.0105166097 | 0.7447399095 |
| C | −4.1180348413 | −0.0544904207 | −0.0804461399 |
| C | −4.8286580347 | −1.2521941077 | −0.2851159924 |
| C | −6.9372849667 | −0.0851314374 | −0.0374780032 |
| C | −6.2385084211 | 1.1160395637 | 0.1653423040 |
| H | −6.8000994268 | 2.0271102893 | 0.3477976936 |
| C | −4.8525514864 | 1.1291327149 | 0.1450693003 |

| | | | |
|---|---|---|---|
| H | −4.3232272938 | 2.0583272519 | 0.3302573081 |
| C | 1.6878187811 | 0.0204255594 | −0.1491672512 |
| C | 2.4148801501 | 1.2151169626 | −0.0034620787 |
| H | 1.8843310393 | 2.1514491423 | 0.1448635244 |
| C | 3.8022860302 | 1.2434433476 | −0.0175822270 |
| H | 4.2986957815 | 2.1994053029 | 0.0934204893 |
| C | 4.5617379340 | 0.0569958451 | −0.1783962273 |
| C | 3.8295356137 | −1.1482627409 | −0.3252103815 |
| H | 4.3476459962 | −2.0913700187 | −0.4464656115 |
| C | 2.4419315461 | −1.1552944529 | −0.3105584648 |
| H | 1.9323829652 | −2.1047963028 | −0.4482952179 |
| H | 0.0395411437 | −1.9738854128 | 0.7276985566 |
| H | −2.4735815556 | 1.9447141382 | −0.9474943209 |
| C | −6.2180578916 | −1.2672782881 | −0.2621094894 |
| H | −4.2859425261 | −2.1700305755 | −0.4867393521 |
| H | −6.7564196583 | −2.1974082199 | −0.4284944434 |
| C | −8.4074394775 | −0.1192090668 | −0.0152989808 |
| N | 5.9387999131 | 0.0740282073 | −0.1912403552 |
| C | 6.7193874668 | −1.1281804817 | −0.4703206857 |
| C | 6.9907126556 | −1.9774293443 | 0.7735267528 |
| H | 7.6677329799 | −0.8049115598 | −0.9113977783 |
| H | 6.2129633968 | −1.7210136282 | −1.2385198246 |
| H | 7.5382086559 | −1.3977304185 | 1.5256272156 |
| H | 7.5933407717 | −2.8560949881 | 0.5140657900 |
| H | 6.0563211222 | −2.3236328329 | 1.2276924515 |
| C | 6.6952629658 | 1.2941318459 | 0.0763915651 |
| C | 6.9286619182 | 2.1488975927 | −1.1713015607 |
| H | 7.6573534335 | 0.9932274240 | 0.5034791444 |
| H | 6.1868008227 | 1.8755289808 | 0.8519072902 |
| H | 7.4778556778 | 1.5816385276 | −1.9316084244 |
| H | 7.5149661346 | 3.0410989121 | −0.9207828472 |
| H | 5.9798661812 | 2.4736292768 | −1.6112832994 |
| O | −9.1267457704 | 0.8508661605 | 0.1736820009 |
| H | −8.8488671982 | −1.1231976327 | −0.1872570044 |

| **35** | *x* | *y* | *z* |
|---|---|---|---|
| C | −1.4568893029 | −0.4469178337 | −0.0243414603 |
| C | −0.8268216923 | 0.6510440470 | −0.6392383681 |
| C | 0.5571468167 | 0.7478996743 | −0.6942565785 |
| H | 1.0046090838 | 1.5887228028 | −1.2148959200 |
| C | 1.3871136102 | −0.2387826648 | −0.1261189411 |
| C | 0.7526515903 | −1.3333521534 | 0.4936822189 |
| C | −0.6311939008 | −1.4386064368 | 0.5372524049 |
| H | −1.0785655040 | −2.2832032550 | 1.0521026800 |
| C | −2.9254469580 | −0.5547017587 | 0.0326111164 |
| C | −3.5615942063 | −1.8146236158 | 0.0640049856 |
| C | −5.7575834957 | −0.7779731378 | 0.1523581861 |
| C | −5.1203579642 | 0.4839232715 | 0.1187576983 |
| H | −5.7292676995 | 1.3831547033 | 0.1496119056 |
| C | −3.7415723332 | 0.5932610762 | 0.0605407122 |
| H | −3.2879181742 | 1.5784085146 | 0.0652865549 |
| C | 2.8562029720 | −0.1285644865 | −0.1795853123 |
| C | 3.5029677640 | 1.1195832624 | −0.2295644693 |
| H | 2.9125882059 | 2.0310401868 | −0.2052101662 |
| C | 4.8841090803 | 1.2358352928 | −0.2844360298 |
| H | 5.3149918497 | 2.2283506665 | −0.3268524523 |
| C | 5.7202154354 | 0.0900597822 | −0.2923975655 |
| C | 5.0692224064 | −1.1690241566 | −0.2385092474 |
| H | 5.6479937005 | −2.0841932010 | −0.2334630633 |
| C | 3.6863751710 | −1.2640756600 | −0.1849122334 |
| H | 3.2417895218 | −2.2549783556 | −0.1679433820 |
| H | 1.3520747288 | −2.0986341930 | 0.9766402109 |
| H | −1.4262220602 | 1.4195486012 | −1.1174744806 |
| C | −4.9390937356 | −1.9303436697 | 0.1228863537 |
| H | −2.9620778374 | −2.7172932457 | 0.0163856862 |
| H | −5.3771454591 | −2.9198135915 | 0.1373697957 |
| C | −7.1961902605 | −0.7767909189 | 0.2186274141 |
| C | −8.1023411410 | −1.8074974192 | 0.2459742042 |
| H | −7.6445821649 | 0.2129219244 | 0.2528769526 |
| N | 7.0912667500 | 0.1941063657 | −0.3498486025 |

| | | | |
|---|---|---|---|
| C | 7.9472798362 | −0.9836572118 | −0.4681657993 |
| C | 8.3019326549 | −1.6118839896 | 0.8813042642 |
| H | 8.8613914998 | −0.6702575507 | −0.9822527861 |
| H | 7.4674815290 | −1.7189084766 | −1.1217322775 |
| H | 8.8229954599 | −0.8888152467 | 1.5192821322 |
| H | 8.9586488730 | −2.4785626530 | 0.7398118462 |
| H | 7.4031361206 | −1.9446068211 | 1.4110897088 |
| C | 7.7672526916 | 1.4881892246 | −0.3031617623 |
| C | −7.7865549594 | −3.1985099383 | 0.2041939226 |
| C | −9.4979460133 | −1.5057182038 | 0.3206388106 |
| N | −7.5579964968 | −4.3394125650 | 0.1709895372 |
| N | −10.6347071352 | −1.2631105255 | 0.3818265071 |
| H | 8.7609795424 | 1.3201787260 | 0.1240560951 |
| H | 7.2439605159 | 2.1482055136 | 0.3956498625 |
| C | 7.9000706674 | 2.1520419531 | −1.6755788223 |
| H | 8.4662945666 | 1.5115357505 | −2.3615327004 |
| H | 8.4272150414 | 3.1096215819 | −1.5883694447 |
| H | 6.9175358087 | 2.3399263950 | −2.1211081850 |

| **34** | *x* | *y* | *z* |
|---|---|---|---|
| C | 2.4989925816 | −0.7714319201 | 0.8532774358 |
| C | 1.6493241026 | −1.7365041003 | 0.2842602615 |
| C | 0.6696993640 | −1.3825602679 | −0.6365549687 |
| H | 0.0459112075 | −2.1600125631 | −1.0675384934 |
| C | 0.4849620088 | −0.0449433069 | −1.0336339404 |
| C | 1.3354215326 | 0.9192039306 | −0.4609834110 |
| C | 2.3178261873 | 0.5654322289 | 0.4568994578 |
| H | 2.9653312982 | 1.3378037032 | 0.8615384265 |
| C | 3.5430265687 | −1.1475706670 | 1.8299000675 |
| C | 3.9068328619 | −0.2786937359 | 2.8782151034 |
| C | 5.5555346944 | −1.8630176547 | 3.7159172453 |
| C | 5.1916192373 | −2.7297196442 | 2.6669168614 |
| H | 5.6985752098 | −3.6869910803 | 2.5739669271 |
| C | 4.2098425413 | −2.3825825548 | 1.7470463686 |
| H | 3.9743139787 | −3.0671212172 | 0.9378335454 |

| | | | |
|---|---|---|---|
| C | −0.5565798086 | 0.3307736612 | −2.0109118363 |
| C | −1.7674824410 | −0.3761686952 | −2.1117530040 |
| H | −1.9592407352 | −1.2094207298 | −1.4414252156 |
| C | −2.7510560322 | −0.0323945025 | −3.0291952719 |
| H | −3.6575720978 | −0.6243437601 | −3.0566027724 |
| C | −2.5809694656 | 1.0595706934 | −3.9172317656 |
| C | −1.3612663146 | 1.7748346122 | −3.8141977827 |
| H | −1.1691778350 | 2.6251967420 | −4.4567440482 |
| C | −0.3895567981 | 1.4144535295 | −2.8906469842 |
| H | 0.5349375403 | 1.9847439175 | −2.8689633872 |
| H | 1.2158799038 | 1.9651234765 | −0.7272982129 |
| H | 1.7478737690 | −2.7776879336 | 0.5772673248 |
| C | 4.8861386390 | −0.6242323683 | 3.7974866576 |
| H | 3.3945293526 | 0.6726890723 | 2.9848632131 |
| H | 5.1300543428 | 0.0720642349 | 4.5938019929 |
| N | −3.5503971076 | 1.4076720125 | −4.8328605590 |
| C | −3.3267539815 | 2.4507682340 | −5.8297383426 |
| C | −3.6628420943 | 3.8559770536 | −5.3249706502 |
| H | −3.9502957683 | 2.2110343213 | −6.6971855725 |
| H | −2.2902326651 | 2.4050995900 | −6.1786884832 |
| H | −4.7132907674 | 3.9155903319 | −5.0176294384 |
| H | −3.4955840063 | 4.5975979450 | −6.1153134799 |
| H | −3.0427530195 | 4.1259544069 | −4.4636847258 |
| C | −4.8538099280 | 0.7503288887 | −4.8592764503 |
| C | −4.8732824586 | −0.5273540489 | −5.7016404687 |
| H | −5.5733219809 | 1.4698509007 | −5.2633461915 |
| H | −5.1768005344 | 0.5407389789 | −3.8346709134 |
| H | −4.5945363585 | −0.3105360621 | −6.7393020985 |
| H | −5.8763825089 | −0.9705335536 | −5.7028668898 |
| H | −4.1700346672 | −1.2701887733 | −5.3107152512 |
| C | 8.0549821092 | −2.0584730508 | 6.6088517144 |
| C | 6.5899997764 | −2.2811557731 | 4.6518329890 |
| C | 7.0309216037 | −1.5881492382 | 5.7313262518 |
| H | 7.0347915295 | −3.2524764619 | 4.4409981581 |
| H | 6.6166231051 | −0.6174806175 | 5.9885578639 |
| N | 8.4405584035 | −1.3432301668 | 7.6559658780 |

| | | | |
|---|---|---|---|
| C | 9.5937048626 | −1.9805178977 | 8.3787678097 |
| C | 9.5083209029 | −3.4522545373 | 7.9261445514 |
| H | 9.4160732503 | −1.8205335145 | 9.4489082198 |
| O | 10.7931181846 | −1.3940304802 | 7.9737428242 |
| C | 8.8433613210 | −3.3327108553 | 6.5264234212 |
| C | 8.5699646151 | −4.2029953851 | 8.8845860247 |
| C | 10.8712545862 | −4.1412975508 | 7.8716883537 |
| H | 8.2068519991 | −4.1960608353 | 6.3049692684 |
| H | 9.5956966327 | −3.2700781447 | 5.7290714277 |
| H | 11.5577068592 | −3.6178124847 | 7.2017485263 |
| H | 10.7505262457 | −5.1693373313 | 7.5106823392 |
| H | 11.3303263450 | −4.1846957969 | 8.8660675212 |
| H | 7.5835890412 | −3.7281442922 | 8.9389295077 |
| H | 8.9891399579 | −4.2308609256 | 9.8968479952 |
| H | 8.4298799250 | −5.2351597580 | 8.5454425433 |
| C | 11.0287561916 | −0.0825052965 | 8.4878372120 |
| H | 10.3586405716 | 0.6530324357 | 8.0328583214 |
| H | 12.0668461904 | 0.1589670564 | 8.2450549423 |
| H | 10.8942084413 | −0.0602219750 | 9.5781550433 |
| O | 7.9982467981 | −0.2050029965 | 8.0434740395 |

| **39** | *x* | *y* | *z* |
|---|---|---|---|
| C | −5.7699270303 | 0.5283542912 | −0.3087464646 |
| C | −5.0306175214 | 1.6932067417 | −0.5259750067 |
| C | −3.6375263254 | 1.6595815889 | −0.4838828784 |
| C | −2.9519571910 | 0.4598576674 | −0.2258054277 |
| C | −3.7089252428 | −0.7047807595 | −0.0101463772 |
| C | −5.1020707112 | −0.6707272907 | −0.0503159078 |
| C | −1.4706859053 | 0.4237362339 | −0.1828958270 |
| C | −0.6987019581 | 1.2390792126 | −1.0276287845 |
| C | 0.6913426959 | 1.2050833182 | −0.9880204155 |
| C | 1.3730730995 | 0.3542456507 | −0.1017090167 |
| C | 0.6010891894 | −0.4610974656 | 0.7430238090 |
| C | −0.7889554646 | −0.4271015564 | 0.7034154453 |
| H | −3.2012379844 | −1.6484288671 | 0.1662491048 |
| H | −1.1923249986 | 1.8850619124 | −1.7474636619 |

| | | | |
|---|---|---|---|
| H | −5.6663067244 | −1.5850717341 | 0.1122760665 |
| H | −6.8555487843 | 0.5547463419 | −0.3405919834 |
| H | −5.5394958624 | 2.6335404431 | −0.7200412142 |
| H | −3.0751184977 | 2.5770988254 | −0.6297090561 |
| H | 1.0947123040 | −1.1070803978 | 1.4628584536 |
| H | −1.3534785830 | −1.0472112464 | 1.3933717022 |
| H | 1.2558658619 | 1.8251928404 | −1.6779767994 |
| C | 2.8543443799 | 0.3181240007 | −0.0587993974 |
| C | 3.6113125958 | 1.4827625229 | −0.2744573606 |
| C | 5.0044580536 | 1.4487088266 | −0.2342874367 |
| C | 5.6723140500 | 0.2496269552 | 0.0241425451 |
| C | 4.9330043770 | −0.9152255393 | 0.2413701217 |
| C | 3.5399131969 | −0.8816001801 | 0.1992775817 |
| H | 2.9775050517 | −1.7991173107 | 0.3451032397 |
| H | 3.1036254168 | 2.4264107945 | −0.4508522027 |
| H | 5.5686943791 | 2.3630532540 | −0.3968785776 |
| H | 6.7579358568 | 0.2232347378 | 0.0559884489 |
| H | 5.4418826175 | −1.8555593888 | 0.4354359392 |
